# Exploring the Adsorption Properties of Small Molecules on CeZr-Based Nanoclusters

**DOI:** 10.1021/acsomega.5c05036

**Published:** 2025-09-13

**Authors:** Raquel C. Bezerra, Felipe V. Calderan, Priscilla Felício-Sousa, Carina S. T. Peraça, Marcos G. Quiles, Juarez L. F. Da Silva

**Affiliations:** † Secretaria de Estado de Educação e Qualidade do Ensino (SEDUC) do Estado do Amazonas, Escola Áurea Pinheiro Braga Av. Perimentral, s/n, Lot. Cidade do Leste, Gilberto Mestrinho, 69089-340 Manaus, AM, Brazil; ‡ Department of Science and Technology, Federal University of São Paulo, 12247-014 São José dos Campos, SP, Brazil; ¶ São Carlos Institute of Chemistry, University of São Paulo, Av. Trabalhador São-Carlense 400, 13560-970 São Carlos, SP, Brazil

## Abstract

An atomistic understanding of the interactions between the molecules and surfaces is crucial to optimize the catalytic performance in heterogeneous systems. In this investigation, calculations based on density functional theory are used to characterize the adsorption of a set of catalytically motivated molecules (CO, CO_2_, CH_4_, NH_3_, H_2_O, SO_2_) on ceria (Ce_15_O_30_), zirconia (Zr_15_O_30_), and mixed ceria-zirconia (Ce_8_Zr_7_O_30_) nanoclusters, which were selected based on their relevance in a variety of catalytic reactions. To obtain an improved analysis of all optimized adsorbed structures, we developed an automated algorithm to characterize the adsorption modes, covering the orientation and site preferences, based on the combination of Coulomb matrix representations with *k-means* clustering, using Silhouette scores to define the number of representative structures. From our calculations and analysis, we found that the 6 closest substrate atoms to the adsorbed molecule provide an optimal representation for the characterization of orientation and site preference of the selected molecules. The adsorption modes of CO, CO_2_, CH_4_, NH_3_, H_2_O, and SO_2_ were grouped into distinct classes, showing consistent orientation patterns, such as parallel or inclined geometries relative to the substrate. In general, the adsorption process does not induce large deformations in the oxide nanoclusters. In the lowest energy structures, the specific interaction preferences of the molecules with the oxide clusters follow the pattern: CO and NH_3_ form bonds via lone pairs on the C and N atoms, respectively; CH_4_ assumes the umbrella configuration; and H_2_O and SO_2_ interact through their O atoms. In particular, the SO_2_ molecule undergoes large changes in the bond angle, indicating a possible deformation toward the SO_3_ molecule, especially in ceria nanoclusters. With the exception of SO_2_, all remaining molecules contribute electron density to the substrate upon adsorption, whereas SO_2_ functions as an electron acceptor (Lewis acid).

## Graphical abstract

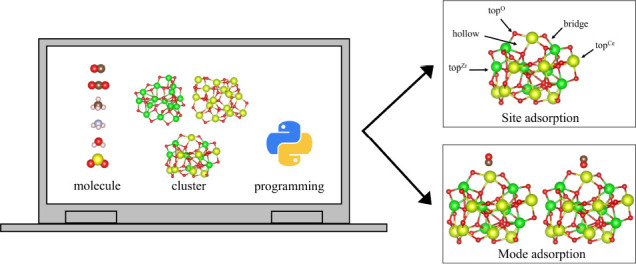

## 1 Introduction

In the field of heterogeneous catalysis, atomistic understanding of the interactions between adsorbate species and substrates (commonly oxides) represents a crucial step in uncovering the reaction mechanisms for several reactions.^1^ Oxide substrates have a wide range of adsorption sites with different coordination along with different chemical environments (acidic and basic sites),^2,3^ which can affect both the activity and selectivity of catalysts. Among a wide range of oxide compounds,^1,4,5^ cerium oxide (CeO_2_) and zirconium oxide (ZrO_2_) have attracted great interest in the last decades due to their roles as catalysts or catalytic supports for various chemical reactions. This interest comes from their unique redox properties^6^ and their oxygen storage capacity,^7^ which is related to the ability of Ce to change its oxidation state from Ce^4+^ (CeO_2_, delocalized *f*-state) to Ce^3+^ (Ce_2_O_3_, localized *f*-state).^8,9^ Furthermore, both compounds show high thermodynamic stability, which can be explained by their high lattice energy and strong cation-oxygen bonds, and hence high melting points.

The synthesis of mixed oxides, exemplified by the combination of CeO_2_ and ZrO_2_ in different proportions (e.g., CeZrO_4_), presents novel avenues for the optimization of their physicochemical attributes for catalytic applications.^4,5,7,9^ Among the characteristics of these materials, the superior thermal stability of mixed oxides stands out compared to the individual ceria and zirconia oxides. This characteristic is crucial for maintaining catalytic performance under high-temperature conditions, which is important for various catalytic applications.^2^ The introduction of zirconium into ceria generates a more robust lattice structure,^10^ thus mitigating the effects of sintering and phase degradation during operation. In addition, the structural and electronic alterations induced by oxide mixing have a pronounced impact on their redox behavior, oxygen storage capacity, and catalytic efficiency.^11^ For example, the incorporation of Zr atoms into the ceria matrix modifies the composition of the crystal phase, commonly stabilizing the cubic fluorite structure and promoting the formation of oxygen vacancies.^4,5,10^ These vacancies enhance oxygen mobility, which is highly advantageous for oxidation and reduction reactions in catalytic processes. Furthermore, electronic interactions between cerium and zirconium modulate the redox behavior of cerium, altering the Ce^4+^/Ce^3+^ ratio and, consequently, its catalytic performance.^4,5^ Thus, CeZrO_4_ has emerged as a promising candidate for applications in automotive catalysts, hydrogen production, and environmental remediation, where proficient redox cycling and high temperature stability are indispensable for optimizing performance.

Considering the importance of these substrates, many adsorbates have been investigated. Carbon monoxide (CO) is a significant molecule involved in numerous vital chemical processes, including Fischer–Tropsch synthesis, which is highly dependent on catalysts. Furthermore, carbon dioxide (CO_2_) is used in a variety of catalytic reactions, such as hydrogenation to yield C_1_ and C_2+_ products,^12^ carbonylation processes,^13^ among others. Another multifaceted compound is CH_4_, which has applications in steam reforming to generate H_2_,^14^ methanol synthesis,^15^ and serving as a gaseous fuel for solid oxide fuel cells. The selective catalytic oxidation of ammonia (NH_3_),^16^ exemplifies another crucial reaction, attracting research interest due to the interaction between the molecule and the substrate, and the environmental concerns associated with its atmospheric emissions. Various reactions involve water on diverse substrates,^17,18^ making the H_2_O-substrate interaction a pivotal step in these processes. Sulfur dioxide (SO_2_), which contributes to the formation of acid rain, can be catalytically reduced to elemental sulfur.

In this work, a theoretical investigation, based on density functional theory (DFT) calculations, was developed to characterize the adsorption behavior of a set of molecules (CO, CO_2_, CH_4_, NH_3_, H_2_O, and SO_2_) on Ce_15_O_30_, Ce_8_Zr_7_O_30_, and Zr_15_O_30_ nanoclusters. These clusters were selected to assess the influence of mixed alloy composition and cluster size on the adsorption properties of the chosen molecular species. The primary results indicate a diverse range of adsorbed configurations, distinguished by variations in adsorption mode, site, and energy. Moreover, the aggregation tendencies of these adsorbed systems was identified. In particular, SO_2_ showed significant alterations in energetic, geometric, and electronic properties, implying enhanced adsorption on the substrates. With the exception of CO, it was observed that the anionic constituent of the molecules primarily interacts with the metal atom of the oxide, suggesting the presence of acid–base interactions.

## 2 Theoretical Approach and Computational Details

We separated this section into two parts, namely (i) a brief overview of the DFT framework, and (ii) a detailed explanation of the algorithms employed to create the adsorption configurations.

### 2.1 Density Functional Theory Calculations

Our calculations were based on the spin-polarized DFT^19,20^ framework within the semilocal Perdew–Burke–Ernzerhof (PBE) formulation for the exchange-correlation energy functional,^21^ which is one of the most used semilocal approximations in computational materials science.^22,23^ An accurate description of the molecules-nanoclusters interactions using plain DFT-PBE faces two challenges: (i) an accurate description of nonlocal weak van der Waals (vdW) interactions,^24^ and (ii) the description of the nature of the Ce *f*-states, which can change from delocalized Ce *f*-states (CeO_2_, Ce^4+^) to localized Ce *f*-states (Ce_2_O_3_, Ce^3+^) under changes in the O-composition,^5,25,26^ transition-metal adsorption on CeO_2_-based substrates,^27,28^ or even the adsorption of molecular species on the CeO_2_ surfaces.^29,30^

Therefore, to improve the accuracy of our DFT-PBE calculations, we used the semiempirical D3 vdW correction proposed by Grimme,^31^ which has been applied in several adsorption studies.^24,29,30,32^ An improved description of the nature of the Ce *f*-states was carried out using the DFT+*U* formulation, as proposed by Dudarev et al.^33^ For that, we used an effective Hubbard parameter (*U*_eff_ = *U* – *J*) of 4.50 eV for the Ce *f*-states, as employed in previous studies.^29,30,34–38^

We employed the all-electron projector augmented wave (PAW) method to describe the interaction between the core and valence electrons,^39,40^ as implemented in the Vienna ab initio simulation package (VASP), version 5.4.4.^41,42^ All calculations were performed using a plane wave cutoff energy of 489 eV, which is 12.5% larger than the highest recommended cutoff energy provided within the selected PAW projectors for the following species: H, C, N, O, S, Zr, and Ce.

Our calculations were carried out using an orthorhombic box with dimensions of 15 × 16 × 17 Å, for molecules in the gas phase and free atoms. For adsorption configurations, we employed a cubic box of 26 Å, in which their periodic images are separated in space by at least 12 Å. The Brillouin zones (BZ) were integrated considering only the Γ-point, since there is no dispersion in the electronic states within the BZ along with a smearing Gaussian parameter of 1 meV to obtain the correct occupation of the electronic states near the highest occupied molecular orbital (HOMO) region. All equilibrium geometries were obtained when the atomic forces in each atom were below 0.025 eVÅ^–1^ using a total energy convergence criterion of 10^–5^ eV. Additional computational details are available in the electronic Supporting Information (SI) file.

### 2.2 Generation of Adsorption Configurations for Molecules on Oxide Nanoclusters

The initial geometries (*x*, *y*, *z* coordinates) for the molecules including carbon monoxide (CO), carbon dioxide (CO_2_), methane (CH_4_), ammonia (NH_3_), water (H_2_O), and sulfur dioxide (SO_2_) were obtained from the National Institute of Standards and Technology (NIST) Computational Chemistry Comparison and Benchmark Database.^43^ The model structures for the Ce_15_O_30_ (Ce^4+^, O^2–^), Ce_8_Zr_7_O_30_ (Ce^4+^, Zr^4+^, O^2–^) and Zr_15_O_30_ (Zr^4+^, O^2–^) oxide nanoclusters were obtained from our previous DFT study,^44,45^ which reported a systematic investigation of the structural, energetic, and electronic properties of the mixed Ce_*n*_Zr_15–*n*_O_30_ oxide nanoclusters. Based on these calculations, mixed oxide Ce_8_Zr_7_O_30_ exhibits the lowest excess energy, indicating its higher relative stability compared to its parent structures. For example, −1.68 eV/nanocluster using the hybrid HSE06 functional^46^ or −1.33 eV/nanocluster using the semilocal PBE functional.^21^ Additional details are discussed elsewhere.^45^

Small oxide nanoclusters are characterized by reduced symmetry. For example, there exist a multitude of nonequivalent cationic top sites (Ce, Zr) and anionic top sites (O), each exhibiting distinct effective coordination environments. In addition, bridge sites are found at the midpoints of the Ce–O and Zr–O bonds, while hollow sites are composed of the Ce, Zr, and O species. Moreover, numerous low-symmetry sites also contribute significantly to the overall structure. Consequently, this assortment of adsorption sites facilitates a broad spectrum of chemical interactions, resulting in a considerable diversity of adsorption properties. For illustrative purposes, certain specified sites are depicted in Figure 1.

**Figure 1:**
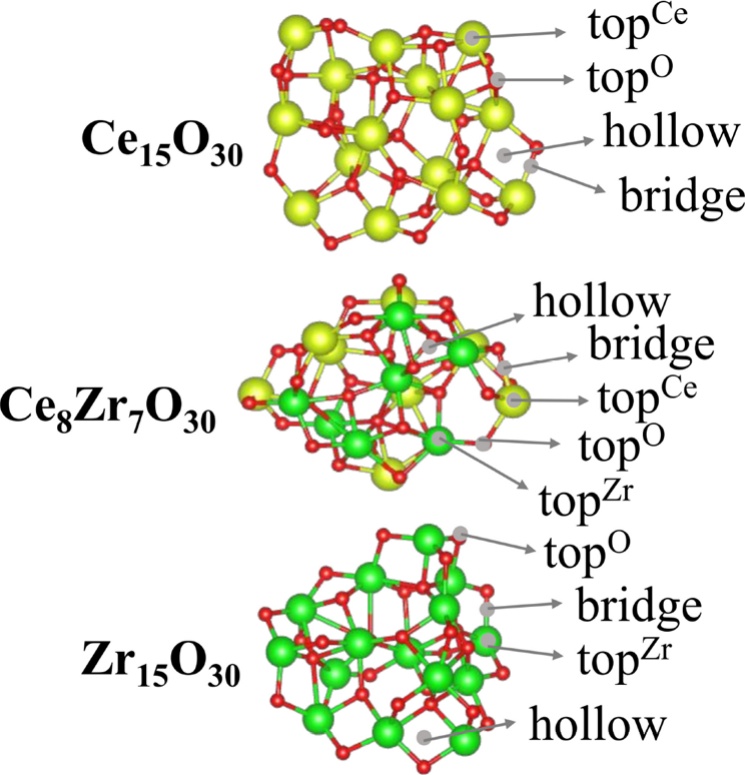
Lowest DFT total energy molecular configurations for the selected oxide nanoclusters (Ce_15_O_30_, Ce_8_Zr_7_O_30_, Zr_15_O_30_), along with the indication of the most important adsorption sites.

A complete exploration of the adsorption sites and the generation of adsorbate structures was carried out using the modified Euclidean similarity distance algorithm, which has been used in several studies within our group.^44,47–51^ With this procedure, we generated from a few thousand up to millions of adsorbate configurations, in which a selected molecule is placed at random positions and orientations at about 2 Å above the nearest atoms of the nanocluster. Thus, our scheme provides a statistical exploration of the nanocluster surface without taking into account chemical principles. In other words, all adsorption sites are treated equally, however, it is not possible to optimize such a large number of configurations (about 10^6^). To solve this problem, we can select only the representative adsorption configurations within the large database of structures. For that, we used the *k-means* ^52^ clustering algorithm combined with the molecular representation of the Coulomb matrix^53^ to reduce the configurations from 10^6^ to about 15 for each system. The reduced set of configurations contains structural features that capture the wide range of adsorption sites.

The *k-means* clustering algorithm is recognized as one of the most widely used algorithms for clustering,^52^ effectively partitioning a data set (molecular structures) into *k*_g_ distinct clusters where each element, or adsorption configuration, is assigned exclusively to a single cluster. The initialization process involves the random placement of seeds *k*_g_, known as centroids, which is succeeded by a series of optimization steps that aim to assign each element to the nearest centroid. This is followed by the centroids being recomputed to the center of mass of their respective clusters.^54^ This process aims to minimize the Within-Cluster Sum of Square (WCSS), which serves as an indicator of the variance in the cluster. The algorithm achieves convergence when the *k-means* no longer produce substantial improvements in the WCSS values.

## 3 Results and Discussion

The results and discussion are organized as follows: examination of the physical-chemical properties of gas-phase systems, configurations of adsorption modes, configurations of structures exhibiting the lowest energy, geometric parameters, interaction and adsorption energies, electron density difference analysis, analysis of charge transfer, and variations in dipole moment consequent to adsorption.

### 3.1 Selected Properties of Molecules and Oxide Nanoclusters

The most important physical-chemical properties of the selected molecules and nanoclusters are outlined in Table 1. As expected, unpaired electrons were not detected for the Zr_15_O_30_, Ce_15_O_30_, and Ce_8_Zr_7_O_30_ nano-oxides, as well as for the molecules CO, CO_2_, CH_4_, NH_3_, H_2_O and SO_2_, which is consistent with our preliminary results^44,45^ and supports the fact that these systems do not have localized electrons.

**Table 1:** Selected Physical–Chemical Properties of Molecules and Nanoclusters in the Gas Phase: Binding Energy Per Atom (*E*_b_), Average Cationic Charge (*Q*_c_), Average Anionic Charge (*Q*_a_), Total Dipole Moment (μ), Average Weighted Bond Length (*d*_av_), the Average Bond Angle (α), and the Average Effective Coordination Number (ECN_av_)

system	theory	*E*_b_(eV)	*Q*_c_ (*e*)	*Q*_a_ (*e*)	μ (D)	*d*_av_ (Å)	α (°)	ECN_av_ (NNN)
CO	PBE+D3	–5.82	0.11 (C)	–0.11 (O)	0.19	1.14		
CO_2_	PBE+D3	–6.00	0.72 (C)	–0.36 (O)	0.00	1.17	180.0	
CH_4_	PBE+D3	–3.64	0.15 (H)	–0.61 (C)	0.00	1.10	109.5	
NH_3_	PBE+D3	–3.27	0.29 (H)	–0.88 (N)	1.50	1.02	106.2	
H_2_O	PBE+D3	–3.38	0.38 (H)	–0.75 (O)	1.81	0.97	104.2	
SO_2_	PBE+D3	–4.11	0.73 (S)	–0.36 (O)	1.54	1.45	119.4	
Zr_15_O_30_	PBE+D3	–7.15	2.19 (Zr)	–1.10 (O)	2.46	2.06		3.35
Ce_15_O_30_	PBE+U+D3	–6.14	2.23 (Ce)	–1.12 (O)	8.96	2.28		4.29
Ce_8_Zr_7_O_30_	PBE+U+D3	–6.64	2.22 (CeZr)	–1.11 (O)	6.76	2.20		4.27

#### 3.1.1 Stability Analysis via Binding Energies

The binding energy (*E*_b_) can be used as a measure of the magnitude to which atoms are bound within a molecular system, such as a molecule or a nanocluster. The calculation is typically performed on a per-atom basis using the following equation,

$$E_{b}=\frac{E_{\mathrm{tot}}^{\mathrm{system}}-\sum_{i=1}^{N_{\mathrm{atoms}}}E_{\mathrm{tot}}^{\mathrm{free}\;\mathrm{atom}\;i}}{N_{\mathrm{atoms}}}$$ 1

where *E*_tot_^system^ denotes the total energy of the molecular system, while *E*_tot_^free atom *i*^ is the total energy of each individual atom *i* in its isolated gas phase form, which requires a spin-polarized calculation. *N*_atoms_ represents the total number of atoms within the molecular system. According to this definition, a more negative *E*_b_ value signifies a more stable system, indicating that a greater amount of energy is required to break the molecular system down into its constituent atoms. The results are summarized in Table 1.

The CO_2_ molecule is recognized as exhibiting the highest per-atom stability (−6.00 eV) among the species examined, as indicated in Table 1. A comparative study of CO and CO_2_ indicates that the incorporation of a second oxygen atom in CO induces only a marginal adjustment in the binding energy per atom, shifting from −5.82 eV for CO to −6.00 eV for CO_2_. The enhanced stability of CO_2_ is attributable to the formation of additional bonds and an extended π-system, which facilitates the enhancement of electron delocalization. Linear CO_2_ geometry with a bond angle of 180.0° further contributes by allowing optimal bond angles, thereby minimizing steric repulsion. In contrast, NH_3_ (−3.27 eV) and H_2_O (−3.38 eV) are classified as the least stable molecules within the selected species, whereas CH_4_ (−3.64 eV) and SO_2_ (−4.11 eV) exhibit intermediate stability levels. Among simple hydrides, methane (CH_4_) is the most stable, followed by water (H_2_O), and ammonia (NH_3_) is the least stable. Consequently, the stability hierarchy for these molecules, ranked from the most to the least stable per atom, is summarized as follows: CO_2_ > CO > SO_2_ > CH_4_ > H_2_O > NH_3_.

These observed trends are subject to several influencing factors: (i) The disparity in electronegativity between C and H is less pronounced than between O and H, or between the N and H atoms. Consequently, this generally results in more covalent C–H bonds within CH_4_, resulting in stronger bonds. As the electronegativity disparity increases (N–H, O–H), the character of the bonds shifts to greater ionic attributes, which can, in turn, influence both the bond strength and the overall stability of the molecule. (ii) CH_4_ exhibits hybridization *sp* ^3^ characterized by a perfect tetrahedral geometry, which acts to minimize electron pair repulsion. In contrast, NH_3_ manifests a trigonal pyramidal geometry accompanied by a lone pair, while H_2_O displays a bent geometry with an angle of 104.2° and two lone pairs. The presence of lone pairs tends to result in increased electron–electron repulsion, which may slightly destabilize the molecule in comparison to configurations with only bonding pairs, thereby reducing their binding energy per atom. SO_2_ presents a significantly more negative binding energy compared to H_2_O and NH_3_, indicative of superior stability, which can be attributed to the ability of sulfur to establish double bonds with oxygen and participate in resonance structures that serve to delocalize electrons and increase stability. The angle of bond of 119.4° implies a bent structure that facilitates effective orbital overlap.

With respect to oxide nanoclusters, computational analyses exhibit substantially elevated binding energies per atom compared to those of the selected molecules. This phenomenon is anticipated because of the robust ionic and covalent interactions intrinsic to the metal–oxygen interactions, and generally, nanoclusters, especially those with many atoms, start to display characteristics closer to those of bulk materials, which helps to justify their use for adsorption studies. The increased number of bonds and the formation of extended networks lead to greater overall stability per atom compared to that of small molecules, where surface effects are more dominant and the number of bonds per atom is limited. Larger clusters typically have higher average coordination numbers, meaning that each atom is bonded to more neighbors, which contributes significantly to the higher binding energy per atom.

The binding energies are measured at −7.15 eV/atom for Zr_15_O_30_, −6.14 eV/atom for Ce_15_O_30_, and −6.64 eV/atom for the mixed Ce_8_Zr_7_O_30_ nanocluster. Among these entities, the pure zirconium oxide nanocluster demonstrates the highest stability. This is because zirconium, as a transition metal, can form strong covalent and ionic bonds with oxygen, leading to a highly stable framework. In addition to that, the relatively high average effective coordination number suggests a compact and well-coordinated structure, contributing to its stability. The pure cerium oxide nanocluster exhibits the lowest stability. Cerium is a lanthanide, and although it forms strong bonds with oxygen, its electronic configuration and bonding characteristics might lead to slightly weaker overall interactions compared to zirconium. The mixed nanocluster has an intermediate stability level, located between the two pure forms. This suggests a synergistic effect in which the introduction of zirconium into the cerium oxide framework enhances its stability compared to that of pure cerium oxide but does not exceed the stability of pure zirconium oxide.

Regarding the stability of mixed nanoclusters, we observed that their calculated binding energy (−6.64 eV/atom) can be close to a weighted average of the binding energies of pure oxide nanoclusters. This weighted average is calculated based on the proportion of cerium (8 atoms) and zirconium (7 atoms) in the mixed oxide nanocluster, using the binding energies of Ce_15_O_30_ and Zr_15_O_30_. The weighted average value (−6.61 eV/atom) deviates from the directly calculated DFT binding energy for Ce_8_Zr_7_O_30_ (−6.64 eV/atom) by merely 0.03 eV/atom, which implies that the stability of the mixed oxide nanocluster, per atom, is largely governed by the relative proportions of its constituent pure oxide characteristics, suggesting a near ideal mixing behavior in terms of the average atomic stabilization energy.

#### 3.1.2 Identification of Cationic and Anionic Sites via Effective Local Charges

To improve our understanding of the interactions between molecules and nanoclusters, we used the density-derived electrostatic and chemical (DDEC) method^55,56^ to evaluate the effective charges in all atoms *Q* ^*i*^. The DDEC method is a charge partitioning approach that assigns atomic charges by distributing the electron density of the system in a way that balances chemical interpretability and electrostatic precision. In contrast to conventional methodologies such as Mulliken, Bader, or Hirshfeld, DDEC is designed to optimize the replication of the electrostatic potential, ensure chemically intuitive trends (e.g., reflecting electronegativity and oxidation states) and preserve the spherical symmetry of atomic densities to enhance transferability.

We separated the effective DDEC charges into cationic (*Q*_c_^*i*^) and anionic (*Q*_a_^*i*^), helping to rationalize the Coulomb contributions to the binding energy. Furthermore, it can also be used to calculate dipole moments (μ) by combining effective charges with the equilibrium DFT bond lengths.^57^ As expected, the presence of polar bonds in molecules is attributed to the difference in electronegativity between atoms, giving rise to effective anionic and cationic charges, which is evident from the calculated effective charges.

We identified a slightly positive (cationic) charge on the C atom in CO, which increases substantially in the C atom of the CO_2_ molecule, as expected from the higher electronegativity of the O atom. As a result, O atoms carry partial negative (anionic) charges, reflecting the electron-withdrawing character of O in these polar covalent bonds. In contrast, in the case of CH_4_, the C atom bears a substantial negative charge, whereas the hydrogen atoms are partially positive. This inversion arises from the relatively higher electronegativity of carbon compared to that of hydrogen, leading to the accumulation of electron density around the central carbon. These variations in the charge distribution across the carbon atom in different molecular environments illustrate how molecular structure and electronegativity differences govern the electronic character of each atom, which in turn will define the nature of molecule–substrate interactions. For example, the presence of exposed cationic or anionic regions in these molecules can direct specific electrostatic or donor–acceptor binding modes on the oxide nanocluster surface.

Similarly, in polar molecules such as NH_3_ and H_2_O, we found partial positive charges on the H atoms and corresponding negative charges on the more electronegative N and O atoms, respectively. For SO_2_, the S atom acquires a partial positive charge, while the O atoms remain anionic, consistent with the asymmetric charge distribution in this bent molecule. Regarding oxide clusters, the metal cations exhibit significant positive charges, ranging from +2.19 *e* for Zr to +2.23 *e* to Ce, reflecting their oxidation states and ionic character. In contrast, oxygen anions show relatively stable charges near −1.11 *e*, largely unaffected by variations in the metal center, indicating a consistent ionic contribution of oxygen between different nanoclusters.

Furthermore, we also evaluated the permanent dipole moments, which reveal important insights into their polarity and potential interaction mechanisms. Molecules such as NH_3_, H_2_O, and particularly SO_2_ display significant dipole moments due to their bent geometries and irregular charge distributions. In contrast, CO_2_ and CH_4_ exhibit zero net dipole moments because of their high symmetry, leading to complete cancellation of the individual bond dipoles. These findings are in agreement with the experimental values reported in the literature.^58^ Oxide nanoclusters also possess sizable dipole moments, especially in the case of Ce_15_O_30_, where asymmetric atomic arrangements and charge separations contribute to strong polarization. These nanocluster dipoles can significantly influence molecule–surface interactions through dipole–dipole or dipole–induced dipole mechanisms, thereby affecting adsorption geometries and binding energies.

#### 3.1.3 Geometric Trends via Effective Coordination Numbers and Bond Lengths

The structural characteristics of the molecules were evaluated using the average bond lengths (*d*_av_) and bond angles (α). The maximum deviation observed was 1.1% for the lengths of the bonds in the molecule CO^59^ and 1.1° for the angles of the bonds in the molecule NH_3_.^60^ For the oxide nanoclusters, the average bond lengths follow the binding energy trends, e.g., the lowest *d*_av_ (2.06 Å for Zr_15_O_30_), correspond to the lowest *E*_b_ (−7.15 eV/per atom).

Regarding the average effective coordination number (ECN_av_), we observed that the mixed oxide is more coordinated; at the same time, its coordination is slightly higher than that of the cerium oxide and with a more pronounced difference in relation to the zirconium oxide. Looking particularly at metals, in mixed oxides, the coordination per atom of Ce is lower than Zr. Then, for the O atom, its ECN_av_ decreases from 3.54 NNN to 2.58 NNN, from cerium oxide to zirconium oxide, respectively, explaining the difference of coordination between both mixed oxides. The weighted average bond distances (*d*_av_) of the mixed oxide are lower than those of cerium oxide and higher than those of zirconium oxide, as expected. This is because the atomic radius and *d*_av_ for each atomic species exhibit the same trend of ECN_av_ in mixed oxide, compared to unmixed oxides.

### 3.2 Characterization of All-Optimized Adsorbed Configurations

According to the methodologies defined in Section 3, an extensive set of calculations was carried out to characterize the adsorption properties of the molecules specified along with the oxide nanoclusters. The following sections will elucidate the predominant trends and conclusions drawn from this analysis.

#### 3.2.1 Adsorption Configuration Energy Profile

We optimized multiple representative configurations for each molecule/nanocluster system, resulting in the identification of both the lowest- and higher-energy configurations, which we refer to as the configuration sets. Figure 2 displays the relative total energies (Δ*E*_tot_) for all systems in their configuration sets, where Δ*E*_tot_ = *E*_tot_^*i*^ – *E*_tot_^lowest^. *E*_tot_^*i*^ is the total energy of a given optimized configuration *i* and *E*_tot_^lowest^ is the lowest energy configuration for each respective set.

**Figure 2:**
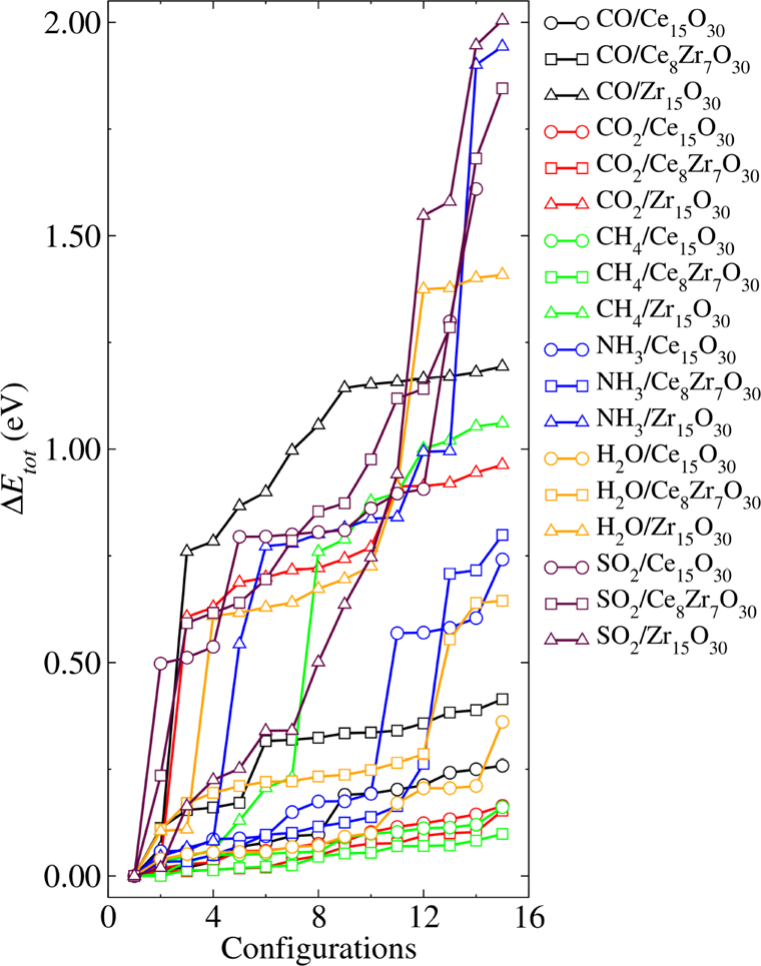
Relative total energies (Δ*E*_tot_) for all optimized molecule/nanocluster configurations, where Δ*E*_tot_ = *E*_tot_^*i*^ – *E*_tot_^lowest ^.

Several interesting trends can be drawn upon analysis of the energy distribution profiles, as shown in Figure 2. First, we observe that the spread in Δ*E*_tot_ varies significantly depending on both the type of molecule and the nanocluster. Generally, configurations involving Zr_15_O_30_ exhibit a much larger energy dispersion compared to those involving Ce_15_O_30_ and Ce_8_Zr_7_O_30_. For most systems, the energy difference between the most and least favorable adsorption configurations in Zr_15_O_30_ ranges from approximately 1.0–2.0 eV. This behavior suggests a strong site sensitivity, due to more pronounced structural rearrangements upon the adsorption of the molecular species, which are energetically costly for certain adsorption geometries.

In contrast, the Ce_15_O_30_ and Ce_8_Zr_7_O_30_ nanoclusters demonstrate a much narrower spread in Δ*E*_tot_, usually within 0.5 eV. This indicates a relatively less sensitive energy landscape with respect to the adsorption site, suggesting a more uniform and possibly chemically saturated surface, where adsorbates experience similar local environments. Furthermore, upon examination of the nature of the adsorbed species, molecules such as CH_4_, CO_2_, and H_2_O generally show a minor dependence on the adsorption site, particularly for nanoclusters containing Ce. This is consistent with their weak physisorption character. In these cases, the interaction is largely driven by van der Waals forces or minor charge redistribution (polarization effects), rather than strong chemisorption, resulting in small energy differences between configurations.

An interesting exception is observed for SO_2_, where a significant energy spread is found not only in Zr_15_O_30_ but also in Ce_15_O_30_ and Ce_8_Zr_7_O_30_. This suggests that SO_2_ interacts more strongly with nanoclusters, possibly involving specific bond formation or notable structural distortion, increasing configurational sensitivity. Thus, this configurational sampling highlights that both the substrate composition and the chemical nature of the adsorbate critically dictate the adsorption energy landscape.

### 3.3 Clustering Algorithm for the Adsorption Mode Characterization

The adsorption process of molecules on substrates involves the creation of either physisorption or chemical bonds between molecules and substrate atoms, determining the dependence of adsorption energy on the adsorption site and molecular orientation. Among several possible configurations, molecules will align in a specific orientation with respect to certain sites on the surface of the nanocluster to achieve the lowest energy state, see Figure 1. Consequently, there exist multiple local minima configurations for the arrangement of chemical species, and a direct classification is straightforward for simple molecules on high-symmetry sites on compact metal surfaces. However, this is not the case on low-symmetry substrates because of the large number and different chemical environments (sites).

Therefore, to overcome these challenges, we developed an in-house computational procedure that categorizes adsorption-mode features to uncover general trends. Our algorithm combines a few steps, which are schematically shown in Figure 3, and discussed below:

1. First, we select the molecular systems to perform the adsorption mode feature analysis. The adsorption process occurs in a local region and, therefore, we select only the molecule and a few atoms on the substrate near the molecule (*N*_c_) for the analysis.
2. Once the region of interest is defined, the Cartesian coordinates of the selected atoms (molecules and substrate) are represented in a vector using the Coulomb matrix representation, which will be inserted into the *k-means* clustering algorithm to identify groups of similar configurations.
3. The *k-means* algorithm cannot determine the number of clustered groups automatically, and therefore alternative strategies are required for that. Here, we used the Silhouette maximum value score to define the number of groups.
4. Due to the statistical nature of the process, for example, initial selection of the centroids, etc., the present process can be performed 100 times, which can improve the quality of the results and insights.

**Figure 3:**
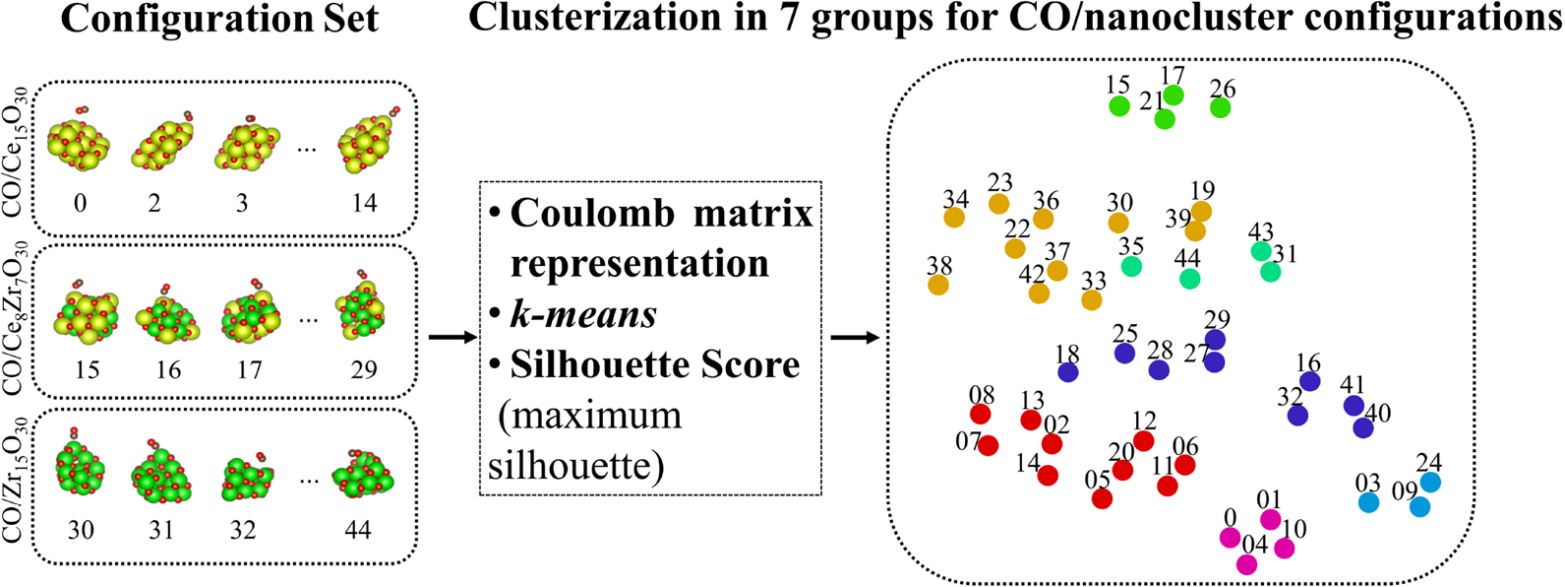
Schematic representation of the adsorption mode analysis using the CO/nanocluster system as an example. The different colors of the balls indicate the different groups resulting from the clusterization.

#### 3.3.1 Adsorption Modes: Orientation and Sites

Here, we present our results obtained with the automatic characterization of the adsorption modes (orientation and site preference) performed for all molecules adsorbed on the nanoclusters. Specifically, approximately 45 configurations were analyzed for CO adsorption on the chosen nanoclusters. The results are presented in Figure 4, in which the number of atoms selected in the group (*N*_c_) was designated as 6 or 8 for comparative analysis. The results for the remaining systems are summarized in the electronic Supporting Information.

**Figure 4:**
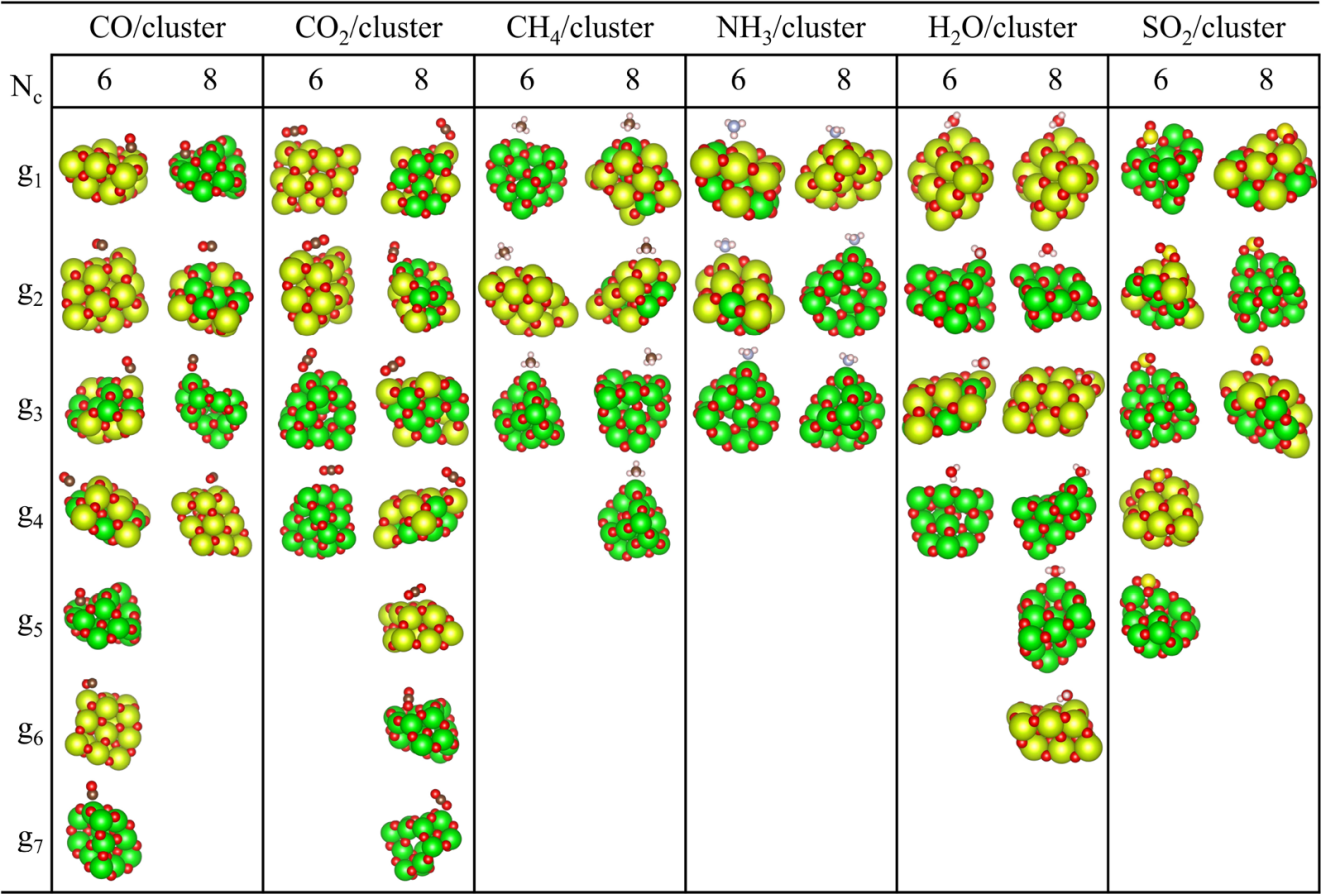
Representative adsorption mode features for the molecule/nanoclusters using two difference values for the number of nanoclusters atoms (*N*_*c*_) considering in the clustering process.

The general findings are the following: (i) The combination of the maximum Silhouette score with the *k-means* algorithm yields excellent results, that is, the most important features of the adsorption mode can be identified without user interference. (ii) The low-symmetry hollow sites on the surfaces of nanoclusters are composed of at least 4 atoms on the surface and one or two atoms below those atoms. Therefore, from our analysis and computational tests, we should consider the *N*_*c*_ values from 4 to 8 substrate atoms, which also depend on the size of the molecules. (iii) Larger values of *N*_c_ increase the number of features of the substrate in the representation vector, and therefore it can create an imbalance toward the features of the substrate. However, it does not increase the number of groups, as shown in Figure 4. (iv) From our computational tests, *N*_c_ = 6 yields the best results, at least for the selected systems, which will be discussed in the following.

From the clustering analysis of the adsorption configurations of CO, we obtained seven groups for *N*_c_ = 6 and four groups for *N*_c_ = 8. These groups revealed that the C–O bond axis is oriented either parallel or perpendicular to the adsorption site within the nanoclusters, with the molecule inclined toward the site. The interaction of CO with the substrate sites occurs by C or O. From the categorization of the adsorption modes, we have seen that CO interacts with a metal atom in oxide through the C atom (M–CO) or the O atom (CO–M) and with a O atom from the substrate by the C atom (O–CO), which is an adsorption structure similar to carbonate or CO_2_, found in literature.^61,62^

For the adsorption configurations of CO_2_, we obtained four groups for *N*_c_ = 6 and seven groups for *N*_c_ = 8. From these findings, we observed that the linear CO_2_ molecule is oriented either in parallel or inclined relative to the sites on the substrate. However, in contrast to CO, perpendicular adsorption was not observed in the cluster. This can be related to the size of CO_2_, which has one more O atom and therefore can have greater contact with the substrate. The adsorption modes are in agreement with previous work for CO_2_ on oxides,^63^ which are the adsorbed structures formed by carbonate species (bridged, bidentate, monodentate and polydentate). Most adsorption modes involve two simultaneous interactions: one between the C atom and the O sites on the oxide, and another between an O atom of CO_2_ and an M site on the substrate.

Considering methane adsorption, the adsorption modes of CH_4_ include umbrella (three H atoms pointed in the direction of the adsorption site), scissoring (interaction of two H atoms), antiumbrella (one H atom interacting with the substrate while keeping the other away).^29,64,65^ In configuration sets for *N*_c_ = 6 and 8 we identified three and four groups, respectively. Among the representative configurations, only the first two modes are observed: (i) the scissor mode in *g*_1_ and *g*_2_ for *N*_c_ = 6 and in *g*_3_ for *N*_c_ = 8, (ii) the umbrella mode in *g*_3_ for *N*_c_ = 6 and in *g*_1_, *g*_2_ and *g*_4_ for *N*_c_ = 8. Such adsorption modes favor O–H interactions, improve system stability, and, in many cases, facilitate molecular activation.^29^

The NH_3_ molecule interacts with the substrate through the N, H, or both atoms. For each value of *N*_c_, we obtained three groups. In the case of *N*_c_ = 6, the two groups exhibited different adsorption modes: (i) in *g*_1_, the H atom is located on an oxygen atom from the substrate, and the twoother H atoms and N atom point away from the substrate, (ii) in *g*_2_ and *g*_3_, the N atom is also on a metal atom, but one of the H atoms points toward an O atom on the substrate. While for *N*_c_ = 8 all groups show an only representative adsorption mode, the interaction between the N atom and the M atom, while the H atoms of the molecule point away from the substrate. Among the modes of ammonia adsorption on the oxide surfaces, a chemical bond tends to be established between the H atoms of the ammonia molecule and the O atoms of the oxide, as well as the coordination of the molecule (through its N atom) with a metal site, acting as a Lewis acid site.^66^ In our observations, these two modes can occur separately or together in the same substrate.

The optimized H_2_O/nanocluster structures showed that interactions are established between the O and H atoms (one or both) of the molecule and the substrate sites. For *N*_c_ = 6, we have a greater variation of the adsorption modes, as we can see in *g*_1_ and *g*_2_ the molecule adsorbs through the axis O–H with the other atom H away from the substrate. In *g*_3_, the molecule adsorbs through the O atom on a metal atom, and the two H atoms point toward O from the nanocluster. In *g*_3_, only the O atom of the molecule is pointed toward the adsorption site, while the H atoms are pointed away from the substrate. Finally, in *g*_4_, the molecule adsorbs through a H atom, keeping the rest away. For *N*_c_ = 8, most of the groups were obtained; however, the adsorption mode is similar, except for *g*_2_, where the two H atoms of the molecule adsorb on the O atoms of oxide, instead of the only one H in g_4_ for *N*_c_ = 6. Our data agree with previous work, where the adsorption modes show the hydrogen bond between the H atom from water and the O atom from oxide^67^ or the interaction between the O atom from water and the metal site.^68^

Lastly, the adsorbed configurations of SO_2_ were separated into five groups using *N*_c_ = 6 and three groups with *N*_c_ = 8. From these results, we can see that the adsorption of SO_2_ occurs through the S and O atoms (by one or both O atoms). In *N*_c_ = 6, two adsorption modes are identified. In the first mode, one of the axes of S–O bonds adsorbs on the substrate in *g*_1_, *g*_2_ and *g*_3_. In the second mode, the two O atoms of the molecule interact with the metals, and the S atom with a O from the nanocluster, in *g*_4_ and *g*_5_. For *N*_c_ = 8, the three highlighted modes are (i) two O and S atoms bind to oxide; (ii) the interaction of the O and S molecule with metal and O atoms from the nanocluster, in *g*_2_; and (iii) the interaction of the two O atoms of SO_2_ with a metal site in the nanocluster; however, S is pointed away from oxide. The preference for these modes of adsorption can be explained by the trends of SO_2_ to adsorb at the basic sites of the oxides.^69,70^

### 3.4 Adsorption Properties of the Lowest Energy Configurations

From this point onward, our analysis focused exclusively on the lowest-energy configurations. Therefore, several properties were evaluated to assess the preferential interactions between the molecules and the substrates. These properties included adsorption and interaction energies, geometric characteristics, electron density variations, atomic charges, and dipole moments.

#### 3.4.1 Adsorption Site Preference

To verify the energetic preference of the adsorbed structure for each nanocluster of molecule/nanocluster, we presented in Figure 5 the lowest energy configurations along their respective adsorption energies. From these configurations, we observed that the molecular positions differ in their inclinations on each substrate. However, the interaction of the adsorbate occurs mainly through the C (CO), O (CO_2_), H (CH_4_), N (NH_3_), O (H_2_O) and O (SO_2_) atoms for all substrate.

**Figure 5:**
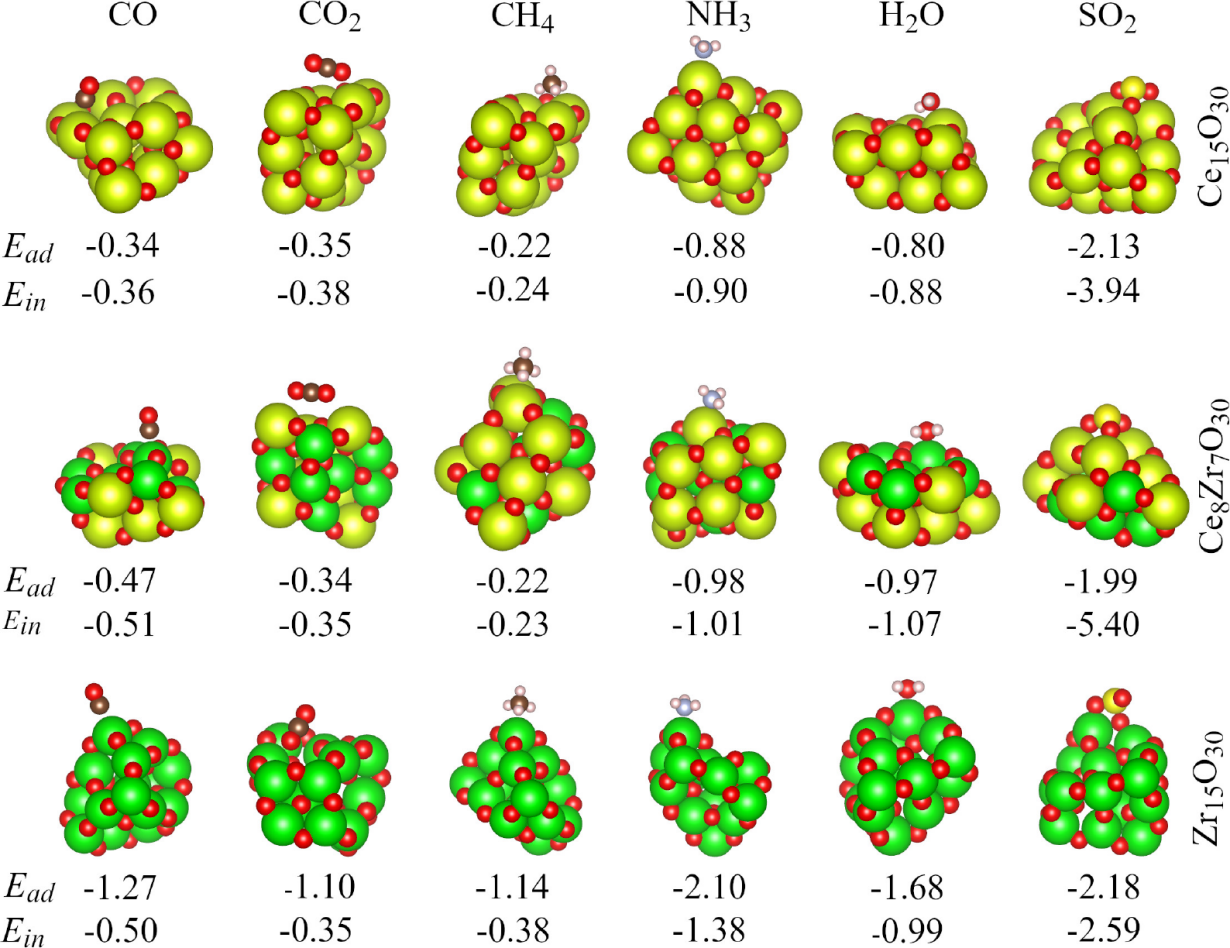
Lowest energy configurations of the molecule/nanocluster systems and their adsorption energies in eV. The *E*_ad_ is taken as the difference between the total energy of the adsorbed system and the sum of the total energy from isolated gas-phase systems.

As we can observe, among the different modes of adsorption, the preferential interaction of CO with the three substrates occurs via the C atom directed to the metal atom, which can be related to interaction by the pair of electrons of the atom alone from the C with the cationic metal, while the CO_2_ interacts mainly by its anionic O atoms with the cationic metal. CH_4_ adopts the mode umbrella as the preferential configuration, establishing an electrostatic interaction between carbon and metal with the H atoms pointed toward the O from the nanocluster. In the case of NH_3_, there is a preference for interaction between the N atom, which also has an electron pair alone, and the metal of the nanocluster. For H_2_O, the interaction is established preferentially by the O atom of the molecule and the metal atom (cationic site) in the nanocluster. And for SO_2_, a chemisorption process results in the formation of SO_3_ species in the three nanoclusters.

#### 3.4.2 Geometric Parameters

To verify changes in the structural properties of the systems, we calculated the *d*_av_, the average ECN_av_, the bond angle (α) and the minimum distance (*d*_min_) of the atom from the molecule to the atom from the nanocluster. For atomic species within a system, *d*_av_ is the average between all bond lengths of this species obtained by a self-consistent procedure, and for the system, it is the average of each *d*_av_. The ECN_av_ is also an average of the coordination number obtained for both each individual atom and the entire system. α was taken as the angle, in degrees, between three atoms in the molecular system and *d*_min_, in angstroms, as the smallest distance of an atom from the molecule of an atom in the nanocluster, which we have expressed as *d*_min_^*c*^ and *d*_min_^*a*^, where the subscripts *c* and *a* refer to the cationic and anionic species of the molecule.

For more details on the structural properties, we presented in Figure 6 the minimum distances of the cationic species (*i* ^*c*^) and the anionic species (*i* ^*a*^) in the molecule of an atom (*j*) in the nanocluster. As expected, most minimum distances are close to 3.0 Å, indicating the possibility of a weak interaction such as physisorption.^24,71^ These interactions do not induce significant changes in the structure of the nanocluster, except for Zr_15_O_30_. For two molecules, the minimum distance is about 2.0 Å, which indicates a change in the behavior of the interactions. From the minimum distances, we still observe that the molecule interacts by the cationic species with an O atom of the substrate and the anionic species with a metal atom of the cluster. The angle of the molecules also does not undergo large variations compared to their respective values in the gas phase in Table 1 (column 8), where the only for the SO_2_ mainly on Ce_15_O_30_ and Ce_8_Zr_7_O_30_ occurs a change of almost 13° in α, which can be explained by the trends of SO_2_ to adopt a structure similar to SO_3_ during the interaction with the oxide.

**Figure 6:**
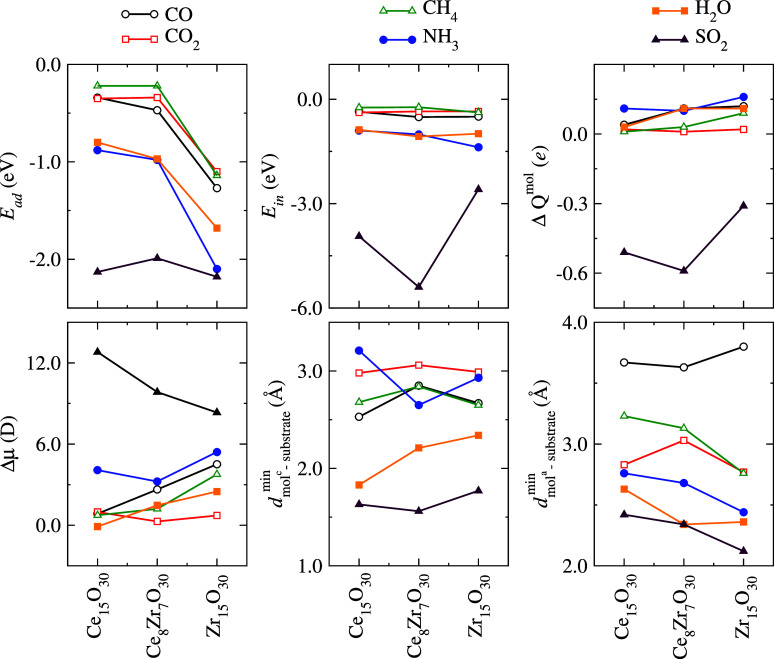
Properties of the lowest energy configurations for adsorption systems: the chemical species, the adsorption energy (*E*_ad_), the binding energy per atom (*E*_b_), the change in the charge of the molecule (Δ*Q* ^mol^), the change in the dipole moment of the molecule (Δμ^mol^), the minimum distance of the cationic and anionic specie (of the molecule) from the nanocluster, and the bond angle (α) of the molecule.

#### 3.4.3 Adsorption and Interaction Energies

The adsorption energy (*E*_ad_) and the interaction energy (*E*_int_) are two important properties that allow understanding of the stability and strength of the interactions between molecules and nanoclusters. Then, we can define the adsorption energy as follows:

$$E_{\mathrm{ad}}=E_{\mathrm{tot}}^{\mathrm{mol}/\mathrm{sub}}-E_{\mathrm{tot}}^{\mathrm{mol}}-E_{\mathrm{tot}}^{\mathrm{sub}}$$ 2

where *E*_tot_^mol/sub^ is the total energy of the system with the adsorbed molecule, and *E*_tot_^mol^ and *E*_tot_^sub^ are the total energies of the isolated molecule and the nanocluster, respectively. This quantity reflects the overall energy change after adsorption, including possible structural relaxations of both the molecule and the substrate. Furthermore, we also calculated the interaction energy using a similar equation.

$$E_{int}=E_{\mathrm{tot}}^{\mathrm{mol}/\mathrm{sub}}-E_{\mathrm{tot}}^{\mathrm{mol}(f)}-E_{\mathrm{tot}}^{\mathrm{sub}(f)}$$ 3

where *E*_tot_^mol(*f*)^ and *E*_tot_^sub(*f*)^ are the total energies of the molecule and substrate in their fixed geometries from the adsorbed configuration.

As shown in Figure 6, the *E*_ad_ values indicate that molecules generally adsorb more strongly on the Zr_15_O_30_ substrate. The difference in adsorption energy compared to the other substrates is significant: up to 1.22 eV for NH_3_, 0.93 eV for CO, 0.92 eV for CH_4_, and similar trends for CO_2_ and H_2_O. This stronger adsorption could be related to more reactive or flexible sites on the zirconia-rich surface. An exception is observed for SO_2_, where the difference in *E*_ad_ across the three substrates is only 0.19 eV, suggesting that this molecule interacts strongly with all three nanoclusters in a similar way.

However, because *E*_ad_ also includes the effect of structural rearrangement, we also examine the values of *E*_int_. The interaction energies show smaller differences across the substrates for most molecules, suggesting that the chemical nature of the oxides does not drastically affect the interaction strength, at least for nonpolar or weakly interacting molecules. A clear exception is observed for the SO_2_ molecule, which shows a variation of 2.81 eV in *E*_int_ between the substrates. This highlights that for certain molecules, the interaction strength can be highly sensitive to the surface composition and electronic structure. In terms of interaction strength, the values *E*_int_ indicate that CO, CO_2_, and CH_4_ exhibit weaker interactions, a characteristic of physisorption. On the other hand, molecules such as NH_3_, H_2_O, and especially SO_2_ show stronger interaction energies, suggesting more chemisorptive behavior. These results point to a higher degree of charge redistribution and orbital overlap in these systems.

#### 3.4.4 Mapping Electron Density Redistribution

The interaction between adsorbates and oxide surfaces is primarily driven by a redistribution of electron density, which plays a crucial role in defining the nature and strength of the resulting chemical bonds. Thus, we used the electron density difference (Δρ­(**r**)) analysis to quantify the redistribution of the electron density after the formation of the adsorbate–substrate bond. It is rigorously defined as the electron density of the fully relaxed system subtracted by the densities of the noninteracting, “frozen” fragments in an identical geometric configuration, which is given by the following equation:

$$\Delta \rho (r)=\rho^{\mathrm{mol}/\mathrm{sub}}(r)-\rho^{\mathrm{mol}(f)}(r)-\rho^{\mathrm{sub}(f)}(r)$$

In this context, ρ^mol/sub^ is the density of the final adsorbed complex, while ρ^mol(f)^ and ρ^sub(f)^ are the densities of the isolated molecule and the substrate, respectively. The results are shown in Figure 7, where the cyan isosurfaces represent regions of *electron depletion*, while the yellow isosurfaces denote regions of *electron accumulation*. The use of a constant isovalue (0.0014 *e*Å^–3^) allows a rigorous and quantitative comparison of the interaction strengths across all systems.

**Figure 7:**
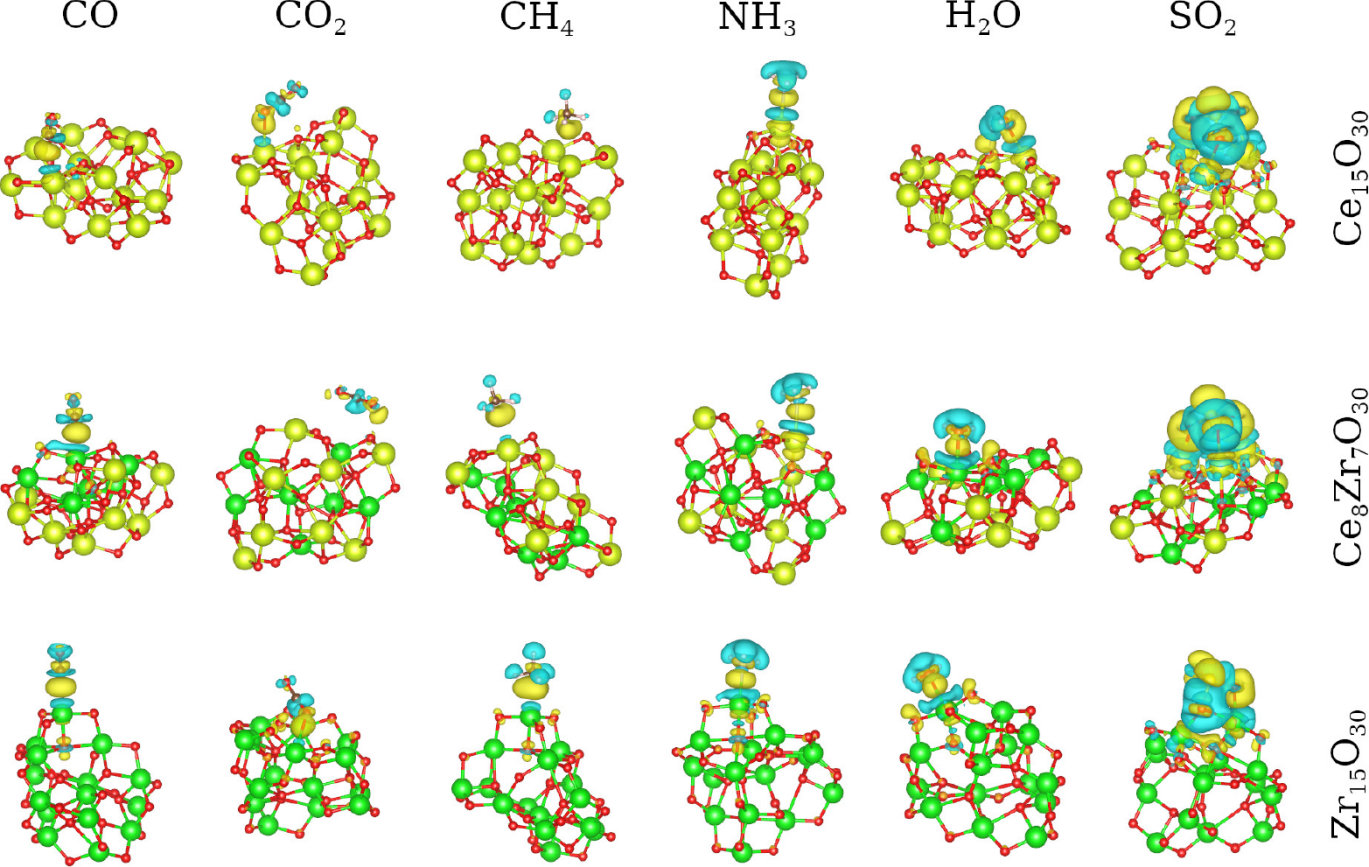
Electron density difference for lowest energy configurations: the isosurfaces were taken within the same level (0.0014 *e*Å^–3^) to provide a fair comparison among the different systems. Yellow and cyan isosurfaces represent regions of electron accumulation and depletion, respectively.

Molecules such as NH_3_ and H_2_O act as classical Lewis bases; that is, the interaction is mainly governed by charge depletion in the N–H or O–H bonds of the molecules and by electron donation from the lone pairs of nitrogen and oxygen to the lowest unoccupied molecular orbitals of the surface, typically associated with the *d* orbitals of the metal cations (Ce^4+^ or Zr^4+^). In contrast, SO_2_ acts as a potent Lewis acid, leveraging its low-lying LUMO (a π* orbital) to accept a significant electron density from surface oxygen anions, which serve as Lewis base sites. This results in a strong charge-transfer interaction, as evidenced by the extensive electron depletion on surface oxygens and accumulation on the SO_2_ molecule (yellow region).

The adsorption of CO is a canonical example of synergistic bonding, which involves primarily two components: (i) A σ donation from the HOMO of CO (localized on the carbon atom) to an empty *d*-orbital on a surface metal cation, and (ii) a π donation from a filled metal *d*-orbital back into the empty π* antibonding orbitals of CO. The interactions of CH_4_ and CO_2_ are fundamentally different. As a nonpolar CH_4_ and saturated molecule, it lacks accessible lone pairs or low-energy unoccupied orbitals for chemical bonding. Its adsorption is governed by weak, long-range London dispersion forces, which arise from transient, correlated fluctuations in the electron clouds of the molecule and the surface. This is confirmed by the minimal charge redistribution observed in the electron density difference plots, characteristic of physisorption and minimum charge transfers, as indicated also by the results in Table 2. In addition, we can observe a higher interaction of CO and CH_4_ with the Zr atoms, while the interaction of CO_2_ with all oxides causes similar changes in charge redistribution.

**Table 2:** Effective Charges in the Molecules upon Adsorption on Oxide Nanoclusters^a^

mol/sub	Zr_15_O_30_	Ce_8_Zr_7_O_30_	Ce_15_O_30_
CO	0.12	0.11	0.04
CO_2_	0.02	0.01	0.02
CH_4_	0.09	0.03	0.01
NH_3_	0.15	0.10	0.11
H_2_O	0.11	0.11	0.03
SO_2_	–0.31	–0.59	–0.51

a Positive values indicate net electron loss (donation to the substrate), and negative values represent net electron gain (acceptance from the substrate).

The outstanding reactivity of ceria, particularly with acceptor molecules such as SO_2_, can be attributed to its facile Ce^4+^/Ce^3+^ redox couple. This flexibility in redox behavior offers a low-energy pathway for electron transfer to SO_2_ from the substrate, thus stabilizing the charge-transfer complex and improving the ionic character of the bond (refer to Table 2). Zirconia is distinguished by the substantial redox stability of the Zr^4+^ cation, which functions as a hard Lewis acid. Consequently, although the charge transfer to SO_2_ is reduced, the unexpected result is a greater redistribution of the electron density for the mixed nanoclusters, as indicated by the increased magnitude of the charge transfer to SO_2_.

#### 3.4.5 Charge Transfer upon Adsorption

To obtain further insights into the nature of electronic interactions at the molecule/substrate interface, we calculated the effective charges on each atom within both the gas-phase and adsorbed molecules using the DDEC method.^55,56^ The effective charge on a molecule upon adsorption, denoted as Δ*Q* ^mol^, is computed as follows:

$$\Delta Q^{\mathrm{mol}}=\sum_{i=1}^{N_{\mathrm{atoms}}\mathrm{mol}}Q_{i}^{\mathrm{mol}}$$ 4

where *Q*_*i*_^mol^ is the effective charge on the atom *i* in the molecule. By construction, Δ*Q* ^mol^ = 0 *e* for isolated gas-phase molecules, while for adsorbed species, Δ*Q* ^mol^ reflects the net charge transfer between the molecule and the substrate. A positive (negative) value of Δ*Q* ^mol^ implies that the molecule loses (gains) electron density upon adsorption, indicating electron donation to (from) the substrate.

As shown in Figure 6 (top-right panel) and Table 2, except the SO_2_ molecule, all remaining adsorbate molecules exhibit small positive Δ*Q* ^mol^ values, generally below +0.15 *e*, indicative of electron transfer from the molecule (Lewis base) toward the substrate. Thus, for these molecules, the substrate functions as an electron acceptor or, more precisely, a Lewis acid.

In contrast, SO_2_ consistently displays negative Δ*Q* ^mol^ values of a large magnitude across all substrates, suggesting a significant charge gain from the substrate. For example, on Zr_15_O_30_, SO_2_ exhibits Δ*Q* ^mol^ = – 0.31*e*, and this charge gain becomes even more pronounced on cerium-based nanoclusters, reaching −0.59 *e* on Ce_8_Zr_7_O_30_ and −0.51 *e* on Ce_15_O_30_. This substantial charge transfer is consistent with a chemisorption mechanism involving sulfur lone-pair interactions with the substrate, corroborated by high interaction energies and pronounced polarization effects. Transfer of electron density from adsorbates induces several significant effects on the substrate: (i) The substrate gains or loses electron density, which can modify its electronic structure and surface potential; (ii) The redistribution of charge across the molecule–substrate interface results in the formation of an interfacial dipole, influencing subsequent adsorption events and chemical reactivity.

#### 3.4.6 Dipole Moment and Polarization Effects

The effects of polarization as a result of molecular adsorption in mixed-oxide nanoclusters are of significant importance. Consequently, we have quantified the alteration in the dipole moment (Δμ^mol^) of the molecules within their most energetically favorable configurations. The modification in the dipole moment was determined by comparing the dipole moment of the molecule in both the adsorbed and gas phases. These findings are illustrated in Figure 6 (bottom-left panel).

The most significant increase in Δμ^mol^ occurs for the SO_2_ molecule on all substrates. These values reach magnitudes above 10 D, particularly in Ce_15_O_30_. This observation suggests a strong charge transfer and polarization effect as a result of substantial charge redistribution during adsorption. These results are consistent with the higher values of the adsorption energy (*E*_ad_) and interaction energy (*E*_int_) obtained for SO_2_, as well as the significant charge transfer (Δ*Q* ^mol^) for this system. On the other hand, molecules like CO_2_ and H_2_O show lower Δμ^mol^ values, generally below 2 D, indicating weaker charge transfer and polarization effects. The CH_4_ molecule, being nonpolar and weakly interacting with the oxide surfaces, exhibits a minimal dipole change, consistent with its low adsorption energy and negligible charge transfer.

Remarkably, the observed trend across the substrates illustrates the influence of composition on the modulation of polarization. For most molecules, with the exception of SO_2_, Δμ^mol^ exhibits a slight increase when transitioning from Ce_15_O_30_ to Zr_15_O_30_. This suggests that zirconium-rich surfaces might induce marginally elevated molecular polarization under specific circumstances. This phenomenon could be attributed to the distinct local electronic structures and acid–base characteristics of the Zr sites compared to Ce.

## 4 Conclusions

In this work, we used spin-polarized DFT-PBE+*U* calculations with D3 dispersion corrections to investigate the adsorption of catalytically relevant small molecules (CO, CO_2_, CH_4_, NH_3_, H_2_O, SO_2_) in ceria (Ce_15_O_30_), zirconia (Zr_15_O_30_) and mixed ceria-zirconia (Ce_8_Zr_7_O_30_) nanoclusters. We developed an automated clustering algorithm that classifies the characteristics of the adsorption mode (adsorbed orientation and site preference) based on Coulomb matrix representations and *k*-means clustering, optimized by Silhouette scoring. The algorithm implemented allowed us to identify representative adsorption configurations that vary in orientation and binding site, while showing that the six atoms closest to the adsorbate (*N*_c_ = 6) optimally describe the interaction environment.

In all systems, adsorption generally occurred with limited geometric distortion of the oxide nanoclusters, although SO_2_ exhibited the strongest interactions, inducing significant structural and electronic changes, including deformation of the bond angle suggestive of the behavior like SO_3_ in ceria. Energetically, SO_2_, NH_3_, and H_2_O showed the most favorable adsorption, consistent with charge transfer analyses indicating substantial electron redistribution. In particular, we observed charge transfers from the nanocluster to the molecules, except for SO_2_, which locally modulates the electron density and the formation of the dipole moment at the molecule–substrate interface. This reinforces the site-specific and directional nature of the interactions.

Structural descriptors, such as ECN and *d*_av_, varied with both the adsorbate and the substrate, especially in the presence of SO_2_. The centroid structures from clustering analysis effectively captured the diversity of adsorption modes observed in different oxides. Moreover, mixed oxide (Ce_8_Zr_7_O_30_) subtly altered adsorption behavior compared to pure systems. Altogether, our findings provide an improved understanding for understanding the interplay between geometry, energetics, and electronic structure in molecule–oxide interactions, supporting the development of automated tools for surface chemistry characterization and catalyst design.

## Supplementary Material



## Data Availability

All DFT calculations were performed using VASP, which can be used under a nonfree academic license. The optimized equilibrium structures for all calculations are provided within a compressed folder using the VASP format (POSCAR files), which is widely used in the literature and can be converted to different formats using available computational tools. Furthermore, we also provided the Python implementation (https://github.com/CIDAG/Adsorption-Analysis/) used to characterize the features of the adsorption mode along with a short user guide to use it (https://github.com/CIDAG/Adsorption-Analysis/blob/main/userguide.md).

## Notes

Data used for the figures, complementary analyses, and additional technical details (PDF)

## Abbreviations

DFT: density functional theory
VASP: Vienna ab initio simulation package
vdW: van der Waals
PAW: projected augmented wave
