# Supplementary material for: Exploring the Adsorption Properties of Small Molecules on CeZr-Based Nanoclusters
Source: ACS Omega. 2025 Sep 13;10(37):42746–59. doi: 10.1021/acsomega.5c05036 (PMC12461434; doi:10.1021/acsomega.5c05036)
Supplement: Supplementary file 1 [file ao5c05036_si_001.pdf]

Electronic Supporting Information File: Exploring the Adsorption Properties of Small Molecules on CeZr-Based Nanoclusters

Raquel C. Bezerra,[*,†] Felipe V. Calderan,[*,‡] Priscilla Felício-Sousa,[*,¶] Carina S. T. Peraça,[*,¶] Marcos G. Quiles,[*,‡] and Juarez L. F. Da Silva[*,¶]

†*Secretaria de Estado de Educação e Qualidade do Ensino (SEDUC) do Estado do Amazonas, Escola Áurea Pinheiro Braga Av. Perimentral, s/n, Lot. Cidade do Leste, Gilberto Mestrinho,* 69089-340, *Manaus, AM, Brazil*

‡*Department of Science and Technology Federal University of São Paulo, São José dos Campos, SP, Brazil*

¶*São Carlos Institute of Chemistry, University of São Paulo, Av. Trabalhador São-Carlense* 400, 13560-970, *São Carlos, SP, Brazil*

E-mail: racosbez@gmail.com; fvcalderan@gmail.com; priscillafelicio@me.com; carinastperaca@gmail.com; quiles@unifesp.br; juarez_dasilva@iqsc.usp.br

Contents

S-1 Introduction **S-4**

S-2 Additional Computational Details **S-4**

S-3 Computational Convergence Tests **S-4**

S-4 Automated Adsorption Site Tool **S-5**

S-4.1 Clustering Algorithm: *k-Means* S-5

S-4.2 Coulomb Matrix S-6

S-4.3 Silhouette Criteria S-6

S-4.4 Finding Adsorption Site S-7

S-4.5 Full clustering process S-8

S-4.6 Output Data S-8

S-4.7 Dependencies and Execution S-9

S-5 Average Bond Length and Effective Coordination Number **S-10**

S-6 Gas-phase Molecules **S-12**

S-7 Gas-phase Nanoclusters **S-13**

S-8 Adsorption Configurations **S-13**

S-8.1 CO on $Ce_{15}O_{30}$ Nanoclusters S-15

S-8.2 CO on $Ce_8Zr_7O_{30}$ Nanoclusters S-16

S-8.3 CO on $Zr_{15}O_{30}$ Nanoclusters S-17

S-8.4 CO_2 on $Ce_{15}O_{30}$ Nanoclusters S-18

S-8.5 CO_2 on $Ce_8Zr_7O_{30}$ Nanoclusters S-19

S-8.6 CO_2 on $Zr_{15}O_{30}$ Nanoclusters S-20

S-8.7 CH_4 on $Ce_{15}O_{30}$ Nanoclusters S-21

S-8.8 CH_4 on $Ce_8Zr_7O_{30}$ Nanoclusters S-22

S-8.9 CH_4 on $Zr_{15}O_{30}$ Nanoclusters S-23

S-8.10 NH_3 on $Ce_{15}O_{30}$ Nanoclusters S-24
S-8.11 NH_3 on $Ce_8Zr_7O_{30}$ Nanoclusters S-25
S-8.12 NH_3 on $Zr_{15}O_{30}$ Nanoclusters S-26
S-8.13 H_2O on $Ce_{15}O_{30}$ Nanoclusters S-27
S-8.14 H_2O on $Ce_8Zr_7O_{30}$ Nanoclusters S-28
S-8.15 H_2O on $Zr_{15}O_{30}$ Nanoclusters S-29
S-8.16 SO_2 on $Ce_{15}O_{30}$ Nanoclusters S-30
S-8.17 SO_2 on $Ce_8Zr_7O_{30}$ Nanoclusters S-31
S-8.18 SO_2 on $Zr_{15}O_{30}$ Nanoclusters S-32

S-9 Adsorption Modes and Sites Preference **S-34**

References **S-40**

S-1 INTRODUCTION

This document provides supplementary technical details and data derived from calculations employed in the adsorption analysis of probe molecules (CO, CO_2, CH_4, NH_3, H_2O, and SO_2) within mixed CeO_2-ZrO_2 nanoclusters.

S-2 ADDITIONAL COMPUTATIONAL DETAILS

Table S-1. Technical details of the PAW-PBE projectors selected for this study. Minimum cutoff energy (`ENMIN`), recommended cutoff energy for the plane-wave basis set (`ENMAX`), number of valence electrons (`ZVAL`) and valence electronic configuration.

Element	Projector name	`ENMIN` (eV)	`ENMAX` (eV)	`ZVAL`	Valence
H	H_GW 21Apr2008	250.000	300.000	1	$1s^1$
C	C_GW_new 19Mar2012	310.494	413.992	4	$2s^2 2p^2$
N	N_GW_new 19Mar2012	315.677	420.902	5	$2s^2 2p^3$
O	O_GW_new 19Mar2012	325.824	434.431	6	$2s^2 2p^4$
S	S_GW 19Mar2012	194.016	258.689	6	$3s^2 3p^4$
Zr	Zr_sv_GW 05Dec2013	259.773	346.364	12	$4s^2 4p^6 5s^0 4d^4$
Ce	Ce_GW 26Mar2009	228.468	304.625	12	$4f^1 5d^1 5s^2 5p^6 6s^2$

S-3 COMPUTATIONAL CONVERGENCE TESTS

In pursuit of achieving convergence for gas-phase nanoclusters, particularly those comprising Ce atoms, we investigated the influence of electron density mixing types. Our empirical analyses suggest that the Kerker mixing method[1] is suitable to achieve electronic convergence. Using the CH_4/nanoclusters systems as a model, we assessed the convergence criteria related to total energy (`EDIFF`) and atomic force (`EDIFFG`). The findings presented in Table S-2 indicate a marginal variation in the total energy of the systems, attributable to minor structural alterations (up to 29.0 meV). Consequently, we incorporated `EDIFF` $= 10^{-5}$ eV and `EDIFFG` $= -0.025$ eV/Å into all computational evaluations.

Table S-2. Evaluation of the convergence criteria for total energy (`EDIFF`) and atomic forces (`EDIFFG`) considering the adsorption systems of CH_4/nanoclusters. For the $Ce_8Zr_7O_{30}$ nanocluster, two cases were considered: one in which the CH_4 molecule adsorbs at a Ce-containing site and the other in which it adsorbs at a Zr-containing site. The difference in total energy and average weighted bond distances as a result of using these different convergence criteria (indicated by Δ) is shown in the third row for each system.

System	`EDIFF` (eV)	`EDIFFG` (eV Å^{-1})	E_{tot} (eV)	d_{min}^{H-Ce} (Å)	d_{min}^{C-Ce} (Å)	d_{min}^{H-Zr} (Å)	d_{min}^{C-Zr} (Å)	d_{min}^{H-O} (Å)	d_{min}^{C-O} (Å)
$CH_4/Ce_{15}O_{30}$	10^{-5}	−0.025	−371.245 477	3.05	3.23			2.68	3.44
	10^{-6}	−0.005	−371.274 695	3.00	3.35			2.69	3.42
	Δ	Δ	0.029 218	0.05	−0.12			−0.01	0.02
$CH_4/Ce_8Zr_7O_{30}$	10^{-5}	−0.025	−414.928 462	2.96	3.13	4.66	5.54	2.84	3.56
	10^{-6}	−0.005	−414.932 416	2.94	3.16	4.76	5.59	2.76	3.58
	Δ	Δ	0.003 954	0.02	−0.03	−0.10	−0.05	0.08	−0.02
$CH_4/Ce_8Zr_7O_{30}$	10^{-5}	−0.025	−414.914 978	4.45	5.37	2.79	3.10	2.58	3.28
	10^{-6}	−0.005	−414.924 388	4.53	5.36	2.77	3.16	2.46	3.36
	Δ	Δ	0.009 410	−0.08	0.01	0.02	−0.06	0.12	−0.08
$CH_4/Zr_{15}O_{30}$	10^{-5}	−0.025	−463.208 507			2.65	2.76	2.83	3.57
	10^{-6}	−0.005	−463.210 557			2.65	2.76	2.84	3.59
	Δ	Δ	0.002 049			0.00	0.00	−0.01	−0.02

S-4 AUTOMATED ADSORPTION SITE TOOL

S-4.1 Clustering Algorithm: k-Means

Clustering algorithms represent significant tools in the domain of machine learning, facilitating the examination of extensive datasets and enabling the extraction of insightful information from data attributes. This process involves the identification of groups within the data in which elements within the same group exhibit greater similarity to each other than to elements in other groups. This similarity between elements can be quantified using a distance metric, such as the Euclidean distance.[2] Of the various clustering techniques available, the *k-means*[3] algorithm was chosen for this study due to its simplicity and the favorable results reported in previous investigations by our research group.[4] *k-means* follows four fundamental steps:

1. Choose k centroids that match k random elements in the dataset;

2. Assign each element to the nearest centroid using Euclidean Distance;
3. Recalculate each centroid of the groups as the center of mass of its members;
4. While the convergence criterion is not achieved, repeat from step 2.[5]

S-4.2 Coulomb Matrix

The Coulomb matrix[6] describes the molecules by their electrostatic interaction between the nuclei, which is given by the following equation,

$$CM_{ij} = \begin{cases} 0.5Z_i^{z_{exp}} & \text{for } i = j; \\ \frac{Z_i Z_j}{D_{ij}^{d_{exp}}} & \text{for } i \neq j. \end{cases} , \quad (1)$$

where Z_i is the charge of the atom i (the same for Z_j in relation to j) and D_{ij} is the Euclidean distance between the atoms i and j. Typically, $z_{exp} = 2.4$ and $d_{exp} = 1$, but these are parameters in this tool, so they can be adapted, depending on the data set and the purpose of the analysis. Despite the inherent ability of the Coulomb matrix to encode the structural properties of chemical systems, it requires transformation before being utilized by *k-means*, as the system necessitates a one-dimensional feature array for each element to be effectively clustered. Consequently, we derive and employ the eigenvalues of the Coulomb matrices to address this requisite.

S-4.3 Silhouette Criteria

A principal parameter within a *k-means* clustering procedure is the initial quantity k, which dictates the number of clusters to be formed. In order to assist specialists in selecting an optimal value k, the tool integrates a system based on the Silhouette criterion[7] to provide recommendations. This criterion helps us to discern which elements are situated within their optimal group and which are not. The silhouette coefficient for a sample i is defined by Equation 2.

$$S_i = \frac{b_i - a_i}{max(a_i, b_i)} , \quad (2)$$

where a denotes the average distance between sample i and the other members of its own group, and b denotes the average distance between i and the samples in the closest neighboring group. S_i can be as low as -1 or as high as 1, where a larger value represents a better separation.

The above formula is valid for individual samples. A common value that can be computed for the whole system, given the silhouettes of the individual samples, is the Silhouette Score or Global Silhouette, shown in Equation 3, i.e.,

$$\mathbb{S} = \frac{\sum_{i=1}^{|\mathbb{X}|} S_i}{|\mathbb{X}|} . \tag{3}$$

In which $\mathbb{S}$ is the global Silhouette and $|\mathbb{X}|$ represents the size of the data set. If the user chooses to have the program automatically recommend a value for k instead of manually specifying it, the following steps are performed to determine this value:

1. For each $k \in \{a + 0s,\ a + 1s,\ a + 2s,\ \ldots,\ b\}$ do:
   (a) Run *k-means* to form k clusters
   (b) Compute $\gamma_k = \sum_{x \in \mathbb{X}} \mathbf{1}_{\{\mathbb{S}_x > \mathbb{S}\}}$
2. Return k corresponding to the largest γ_k computed.

where a is the starting value, b is the ending value, and s is the search space step for K.

S-4.4 Finding Adsorption Site

Determining the adsorption site on the substrate constitutes a critical preliminary step prior to initiating the clustering process. The user is required to specify the number of atoms that the site must encompass. Upon provision of this parameter and subsequent loading of the dataset, the program proceeds to execute the ensuing procedure:

1. Generate two groups of atoms: M (molecule) and N (substrate);
2. Calculate the Euclidean distance between M and N atoms;
3. Sort by ascending order;

4. Filter out repeated atoms belonging to N;
5. Pick the `site_size` first candidates.

The chosen candidates will be the substrate atoms that are considered to be part of the adsorption site.

S-4.5 Full clustering process

Combining all of the methods discussed in this section, we build the core pipeline of the adsorption analysis tool.

1. For each system $x \in \mathbb{X}$, do:
   (a) Calculate the hypothetical adsorption site of x
   (b) Create a new partial system composed of the molecule and adsorption site of x
   (c) Calculate the eigenvalues of the Coulomb Matrix of this partial system
2. Create a table $\mathbb{X}_v$, where each row is composed of the eigenvalues of an $x \in \mathbb{X}$.
3. Cluster elements from $\mathbb{X}_v$ using one of the following methods:
   (a) Simple *k-means* with k manually set by the user
   (b) Silhouette Analysis with k automatically recommended by the program
4. Save the clustering information and proceed to the output stage.

S-4.6 Output Data

As the program searches for the recommended k, it generates Principal Component Analysis (PCA)[8] visualizations, displaying the centroids or the lowest-energy elements of each cluster, depending on the user's setup, for each value of k. Additionally, it provides the silhouette scores for each k and, if requested, also includes t-distributed Stochastic Neighbor Embedding (t-SNE)[9]. After the clustering is done, the program

outputs the final PCA, Silhouette chart, and optionally, t-SNE. It also outputs a text file containing the following information:

1. The number k of clusters;
2. Silhouette score for k;
3. Number of atoms in the site;
4. Fixed Z value (`fixed_z`);
5. Coulomb Matrix exponents (`z_exp` and `d_exp`);
6. If the dataset was scaled (`scale_dataset`);
7. Labels and substrate Z averages;
8. Centroids or Lowest-energy elements;
9. Substrate information;
10. PCA explained variance by each component.

Finally, a directory structure containing the XYZ files separated by cluster identification number is created.

S-4.7 Dependencies and Execution

The tool uses Anaconda[1] to manage its dependencies. The most important packages used are NumPy,[2] SciPy,[3] Scikit-learn,[4] Matplotlib,[5] and tqdm.[6] Then, having the appropriate Anaconda (or equivalent) virtual environment with the correct dependencies enabled, call the program from the command line:

```
python3 adsorption_analysis.py input.json
```

[1]https://www.anaconda.com/
[2]https://numpy.org/
[3]https://scipy.org/
[4]https://scikit-learn.org/
[5]https://matplotlib.org/
[6]https://tqdm.github.io/

where `input.json` is the configuration file, which allows the user to change different execution parameters, making the tool flexible enough to be used with different datasets. Table S-3 shows all the different configuration parameters that can be set, along with their types and brief descriptions.

Table S-3. Configuration file execution parameters descriptions.

Field	Type	Description
system_name	string	Name used in charts
input_folder_path	string	XYZ folder path
output_folder_path	string	Output folder path
method_of_k_selection	string	Method to select number K of clusters ("user" or "silhouette")
number_k_of_clusters	int	If method_of_k_selection = "user", manually set K
silhouette_range	int[]	If method_of_k_selection = "silhouette", look for K in range (from, to, step)
number_of_random_runs	int	Number of executions with random seed for statistical significance
molecule_indices	int[]	Indices of the adsorbed molecule's atoms
site_size	int	Refer to subsection S-4.4
fixed_substrate_atomic_number	int	Replace the atoms of the substrate by the specified Z
z_exp	float	Z exponent for Coulomb Matrix (subsection S-4.2)
d_exp	float	D exponent for Coulomb Matrix (subsection S-4.2)
use_energy	bool	Use energy from XYZ to find representatives
scale_dataset	bool	Remove mean and scale to unit variance
projection_numbers	bool	Systems ID number in the PCA and t-SNE
projection_centroids	bool	Centroids/lowest energy representatives in PCA
projection_tsne	bool	Generate t-SNE plots

S-5 AVERAGE BOND LENGTH AND EFFECTIVE COORDINATION NUMBER

The average bond lengths (d_{av}) and effective coordination number (ECN) constitute essential structural parameters that facilitate the analysis of atomic coordination within symmetric or distorted systems, thereby enabling the detection of potential structural

distortions. According to the concept of an effective coordination number,[10] it is possible to determine the ECN^i and d_{av}^i of an atom i by:

$$ECN^i = \sum_{\substack{j=1 \\ i \neq j}} \exp\left[1 - \left(\frac{2d_{ij}}{d_{av}^i + d_{av}^j}\right)^6\right] , \tag{4}$$

with

$$d_{av}^i = \frac{\sum\limits_{\substack{j=1 \\ i \neq j}} d_{ij} \exp\left[1 - \left(\frac{2d_{ij}}{d_{av}^{i,old} + d_{av}^{j,old}}\right)^6\right]}{\sum\limits_{\substack{j=1 \\ i \neq j}} \exp\left[1 - \left(\frac{2d_{ij}}{d_{av}^{i,old} + d_{av}^{j,old}}\right)^6\right]} . \tag{5}$$

The initial values for $d_{av}^{i,old}$ and $d_{av}^{j,old}$ correspond to the minimum distances between the i and j atoms. These values are subsequently refined during the self-consistent procedure. Ultimately, the average bond length and the effective coordination number for a given system are determined, respectively, by:

$$d_{av} = \frac{1}{N} \sum_{i=1}^{N} d_{av}^i , \tag{6}$$

and

$$ECN = \frac{1}{N} \sum_{i=1}^{N} ECN^i . \tag{7}$$

To understand the effects of molecular adsorption on nanoclusters, we calculated the variations in ECN and d_{av} after adsorption, using the gas-phase nanocluster as a reference, as follows:

$$\Delta d_{av} = (d_{av} - d_{av}^{gp}) \times 100/d_{av}^{gp} , \tag{8}$$

and

$$\Delta ECN = (ECN - ECN^{gp}) \times 100/d_{av}^{gp} , \tag{9}$$

where the superscript gp indicates the property of the structure in gas-phase.

S-6 GAS-PHASE MOLECULES

Table S-4. Energetic properties of gas-phase molecules. Total energy (E_{tot}), binding energy per atom (E_b), enthalpy of formation, standard ($\Delta_f H^o$ exp.) at 298.15 K, energy of the highest occupied molecular orbital (HOMO, ϵ_H), energy of the lowest occupied molecular orbital (LUMO, ϵ_L) and HOMO–LUMO energy gap (E_g). The E_b was determined using the following free-atom energies: −1.115 599 48 eV (H), −1.375 230 77 eV (C), −3.127 775 37 eV (N), −1.899 547 50 eV (O) and −0.869 571 90 eV (S).

Molecule	E_{tot} (eV)	E_b (eV)	$\Delta_f H^o$ exp. (eV)[11]	ϵ_H (eV)	ϵ_L (eV)	E_g (eV)
CO	−14.908 885 00	−5.82	−1.14	−9.02	−2.06	6.96
CO_2	−23.173 044 52	−6.00	−4.08	−9.01	−0.93	8.08
CH_4	−24.033 238 76	−3.64	−0.77	−9.38	−0.42	8.96
NH_3	−19.549 171 79	−3.27	−0.48	−6.19	−0.75	5.43
H_2O	−14.265 491 96	−3.38	−2.50	−7.19	−0.96	6.23
SO_2	−17.010 564 81	−4.11	−3.08	−7.99	−4.48	3.51

Table S-5. Structural and electronic properties of gas-phase molecules. Average bond lengths (d), average bond angles (α), experimental values of bond lengths (d^{exp}) and angles (α^{exp}), percentage change in bond lengths and angles compared to experimental values ($|\Delta d|$ and $|\Delta\alpha|$), average cationic and anionic charges calculated with the DDEC6 approach (Q_c and Q_a), calculated and experimental dipole moments (μ and μ^{exp}).

Molecule	d (Å)	α (°)	d^{exp} (Å)	α^{exp} (°)	$\|\Delta d\|$ (%)	$\|\Delta\alpha\|$ (°)	Q_c (e)	Q_a (e)	μ (D)	μ^{exp} (D)[11]
CO	1.14	-	1.128[12]	-	1.1	-	0.11	−0.11	0.19	0.00
CO_2	1.17	180.0	1.160[13]	180.0[14]	0.9	0.0	0.72	−0.36	0.00	0.00
CH_4	1.10	109.5	1.092[13]	109.5	0.7	0.0	0.15	−0.61	0.00	0.00
NH_3	1.02	106.2	1.024[13]	107.3[13]	0.4	1.1	0.29	−0.88	1.50	1.47
H_2O	0.97	104.2	0.972[13]	104.5[13]	0.2	0.3	0.38	−0.75	1.81	1.85
SO_2	1.45	119.4	1.435[13]	119.4[13]	1.0	0.0	0.73	−0.36	1.54	1.63

S-7 GAS-PHASE NANOCLUSTERS

Table S-6. Energetic properties of gas-phase nanoclusters. Total energy (E_{tot}), relative energy with respect to the PBE framework (ΔE_{tot}), binding energy (E_b), energy of the highest occupied molecular orbital (HOMO, ϵ_H), energy of the lowest occupied molecular orbital (LUMO, ϵ_L) and HOMO–LUMO energy gap (E_g). The E_b was determined using the following free-atom energies: −1.576 359 21 eV (Ce with PBE), −0.914 549 06 eV (Ce with PBE+U), −3.964 441 05 eV (Zr) and −1.899 547 50 eV (O).

Nanocluster	Framework	E_{tot} (eV)	ΔE_{tot} (eV)	E_b (eV)	ϵ_H (eV)	ϵ_L (eV)	E_g (eV)
$Ce_{15}O_{30}$	PBE	−371.067 034 44	0	−6.45	−5.12	−3.43	1.69
$Ce_{15}O_{30}$	PBE+D3	−373.112 578 86	−2	−6.50	−5.11	−3.42	1.69
$Ce_{15}O_{30}$	PBE+U	−344.974 022 86	26	−6.09	−5.09	−3.09	2.00
$Ce_{15}O_{30}$	PBE+D3+U	−347.004 670 15	24	−6.14	−5.09	−3.09	2.00
$Ce_8Zr_7O_{30}$	PBE	−402.631 838 13	0	−6.78	−5.53	−3.78	1.75
$Ce_8Zr_7O_{30}$	PBE+D3	−404.903 299 66	−2	−6.83	−5.52	−3.77	1.75
$Ce_8Zr_7O_{30}$	PBE+U	−388.425 817 33	14	−6.59	−5.51	−3.23	2.28
$Ce_8Zr_7O_{30}$	PBE+D3+U	−390.688 533 98	12	−6.64	−5.50	−3.23	2.27
$Zr_{15}O_{30}$	PBE	−435.851 646 55	0	−7.10	−6.15	−2.27	3.87
$Zr_{15}O_{30}$	PBE+D3	−438.047 630 95	−2	−7.15	−6.16	−2.27	3.89

Table S-7. Geometric properties of gas-phase nanoclusters. Average effective coordination number (ECN) and average weighted bond length distance (d_{av}).

Nanocluster	Theory	$ECN^{\text{nanocluster}}$ (NNN)	$d_{av}^{\text{nanocluster}}$ (Å)	ECN^{Ce} (NNN)	d_{av}^{Ce} (Å)	ECN^{Zr} (NNN)	d_{av}^{Zr} (Å)	ECN^{O} (NNN)	d_{av}^{O} (Å)
$Ce_{15}O_{30}$	PBE	4.27	2.26	5.70	2.22	-	-	3.56	2.29
$Ce_{15}O_{30}$	PBE+D3	4.27	2.26	5.70	2.22	-	-	3.55	2.28
$Ce_{15}O_{30}$	PBE+U	4.27	2.28	5.71	2.23	-	-	3.55	2.30
$Ce_{15}O_{30}$	PBE+D3+U	4.27	2.28	5.71	2.23	-	-	3.54	2.30
$Ce_8Zr_7O_{30}$	PBE	4.26	2.19	4.93	2.20	6.63	2.11	3.53	2.21
$Ce_8Zr_7O_{30}$	PBE+D3	4.26	2.19	4.93	2.20	6.64	2.11	3.53	2.21
$Ce_8Zr_7O_{30}$	PBE+U	4.28	2.20	4.97	2.22	6.63	2.11	3.54	2.22
$Ce_8Zr_7O_{30}$	PBE+D3+U	4.29	2.20	4.97	2.22	6.65	2.11	3.55	2.22
$Zr_{15}O_{30}$	PBE	3.34	2.06	-	-	4.86	2.05	2.58	2.06
$Zr_{15}O_{30}$	PBE+D3	3.35	2.06	-	-	4.87	2.05	2.58	2.06

S-8 ADSORPTION CONFIGURATIONS

In this section, the configurations employed to assess the adsorption of probe molecules (CO, CO_2, CH_4, NH_3, H_2O, and SO_2) on mixed nanoclusters CeO_2-ZrO_2 ($Ce_{15}O_{30}$, $Ce_8Zr_7O_{30}$, and $Zr_{15}O_{30}$) are presented. The optimized structures alongside their investigated properties are depicted in Figures S-1–S-36. For each adsorption system,

the structures are ranked according to their relative total energy, with reference to the lowest-energy configuration. Substrate atoms are portrayed as lime yellow, green, and red spheres to signify Ce, Zr, and O, respectively. The atoms within molecules are illustrated as brown, red, white, light blue, and yellow spheres to signify C, O, H, N, and S, respectively.

The adsorption energy (E_{ad}) was quantified as the energy differential between systems in which molecules or nanoclusters are adsorbed or in a gas phase. The energy gap HOMO-LUMO (E_g) represents the energy disparity between the highest occupied molecular orbital (HOMO, ϵ_H) and the lowest unoccupied molecular orbital (LUMO, ϵ_L). The structural attributes of the substrate were assessed by examining variations in the average effective coordination number (ΔECN^{X}) and the average weighted bond length distance (Δd_{av}^{X}) for each atomic species in the substrate (X = Ce, Zr, and O). For the nanocluster, the parameters $\Delta ECN^{nanocluster}$ and $\Delta d_{av}^{nanocluster}$ are utilized. In adsorbate molecules, the shortest distance ($d_{i-\mathrm{X}}$) between molecular atoms (i = C, $\mathrm{O^m}$, H, N, and S) and atomic species in the substrate (X = Ce, Zr, and O) was assessed. To distinguish between O atoms within molecules, the label $\mathrm{O^m}$ was used. The bond angles of all adsorbates, excluding the CO molecule, were also measured (α).

S-8.1 CO on $Ce_{15}O_{30}$ Nanoclusters

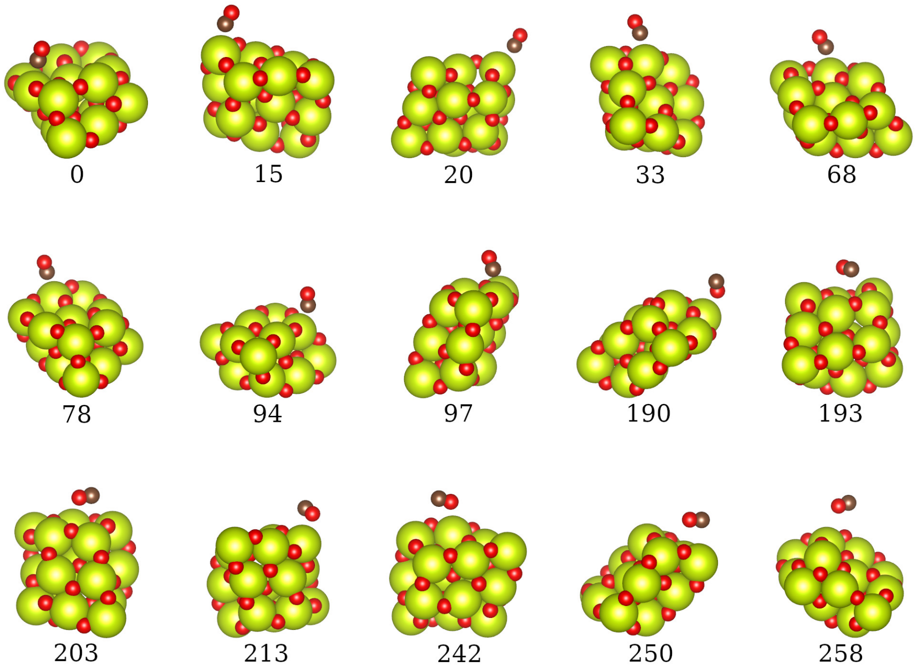

Figure S-1. Optimized $CO/Ce_{15}O_{30}$ structures. The relative total energy with respect to the lowest-energy configuration is displayed below each structure. All values are in meV.

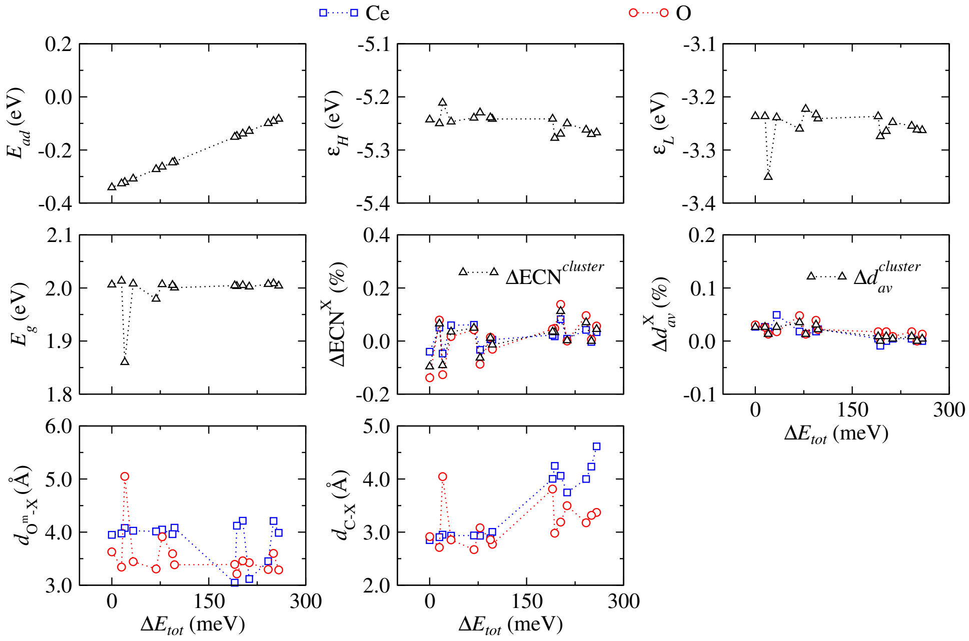

Figure S-2. Energetic, electronic, and structural properties for the $CO/Ce_{15}O_{30}$ systems. The description of each property is provided at the start of Section S-8.

S-8.2 CO on $Ce_8Zr_7O_{30}$ Nanoclusters

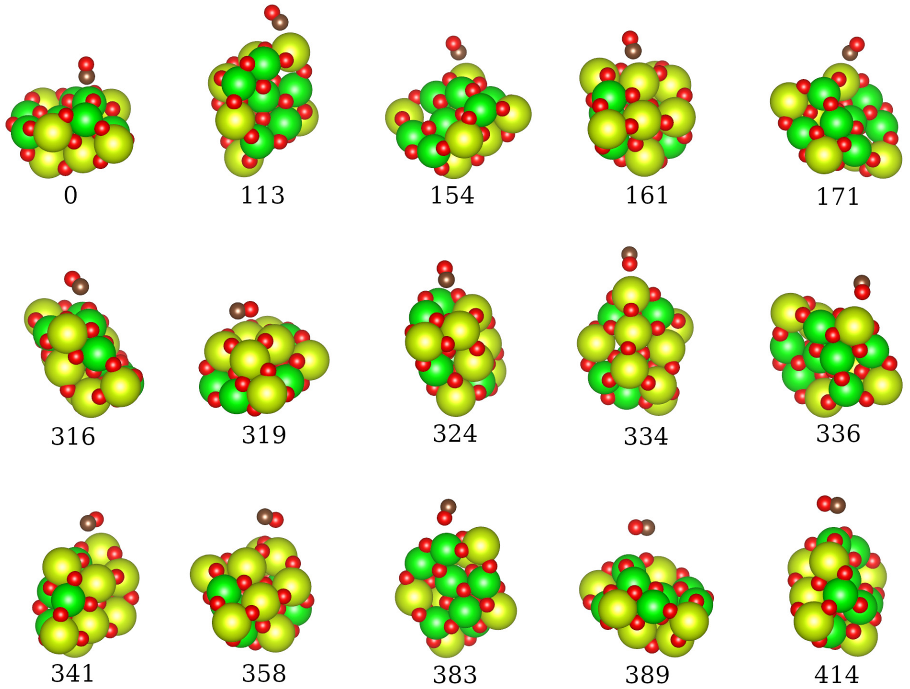

Figure S-3. Optimized $CO/Ce_8Zr_7O_{30}$ structures. The relative total energy with respect to the lowest-energy configuration is displayed below each structure. All values are in meV.

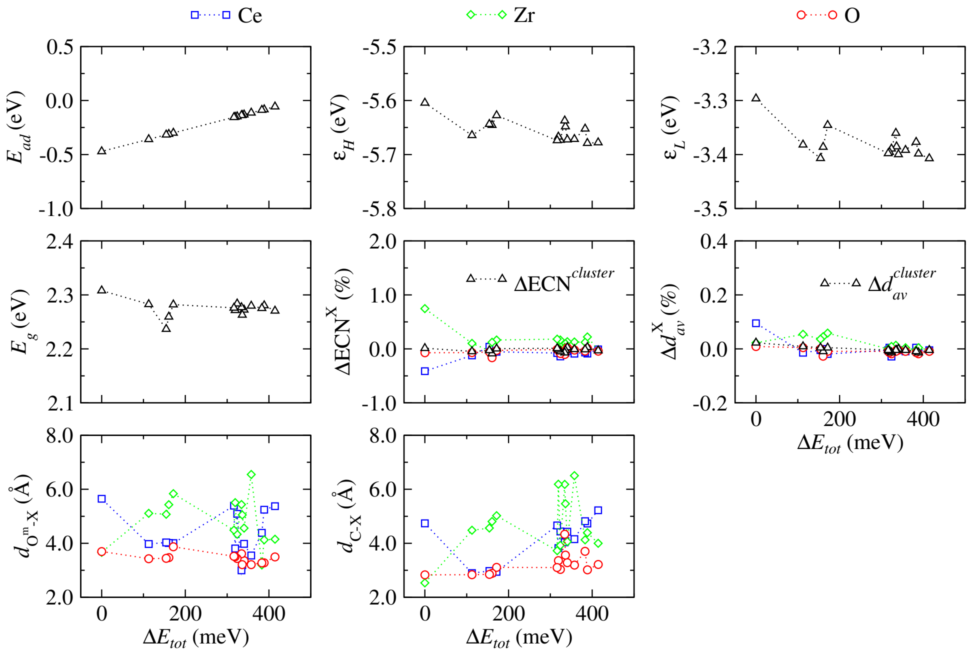

Figure S-4. Energetic, electronic, and structural properties for the $CO/Ce_8Zr_7O_{30}$ systems. The description of each property is provided at the start of Section S-8.

S-8.3 CO on $Zr_{15}O_{30}$ Nanoclusters

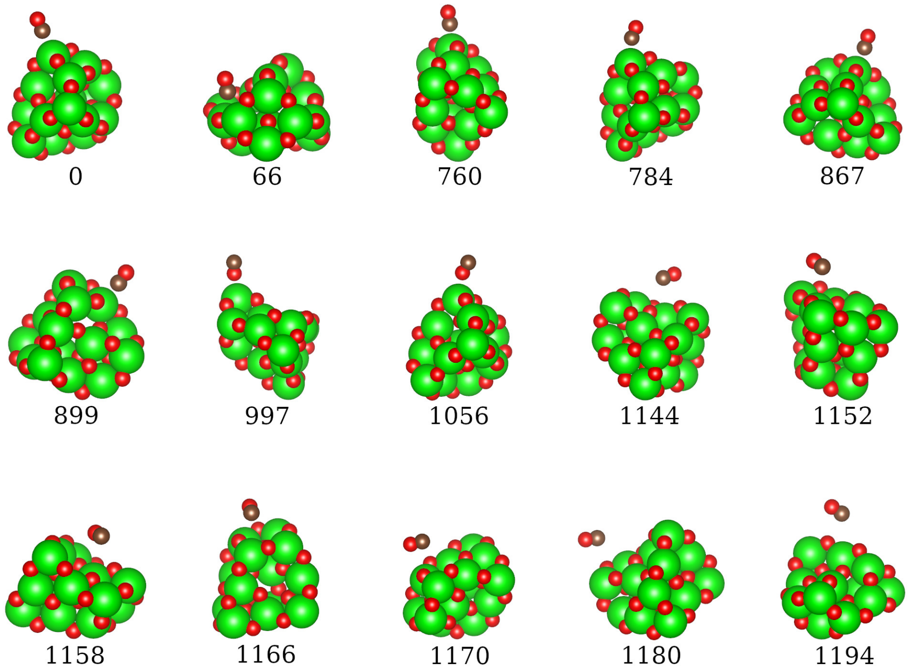

Figure S-5. Optimized $CO/Zr_{15}O_{30}$ structures. The relative total energy with respect to the lowest-energy configuration is displayed below each structure. All values are in meV.

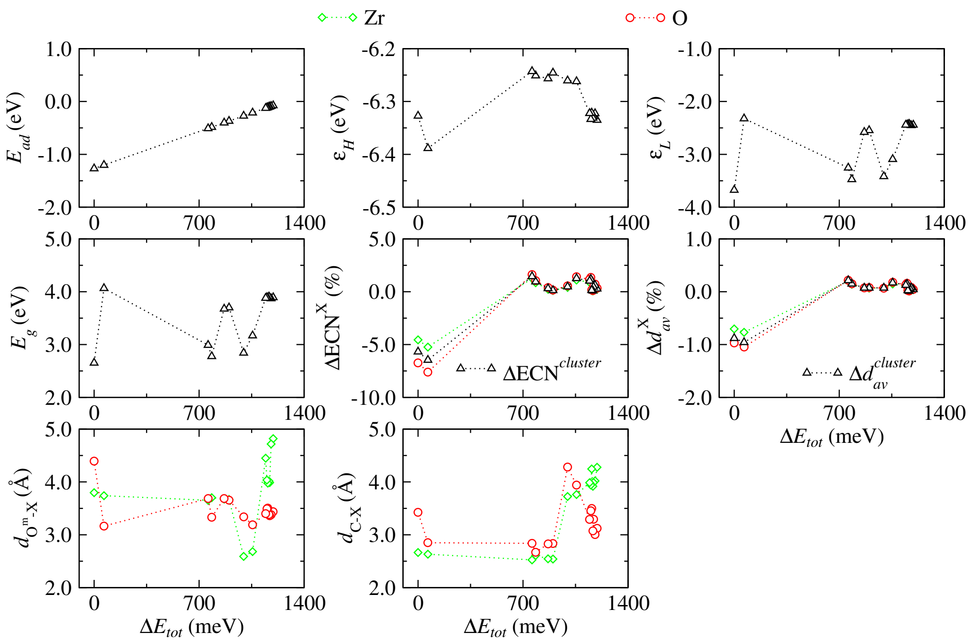

Figure S-6. Energetic, electronic, and structural properties for the $CO/Zr_{15}O_{30}$ systems. The description of each property is provided at the start of Section S-8.

S-8.4 CO_2 on $Ce_{15}O_{30}$ Nanoclusters

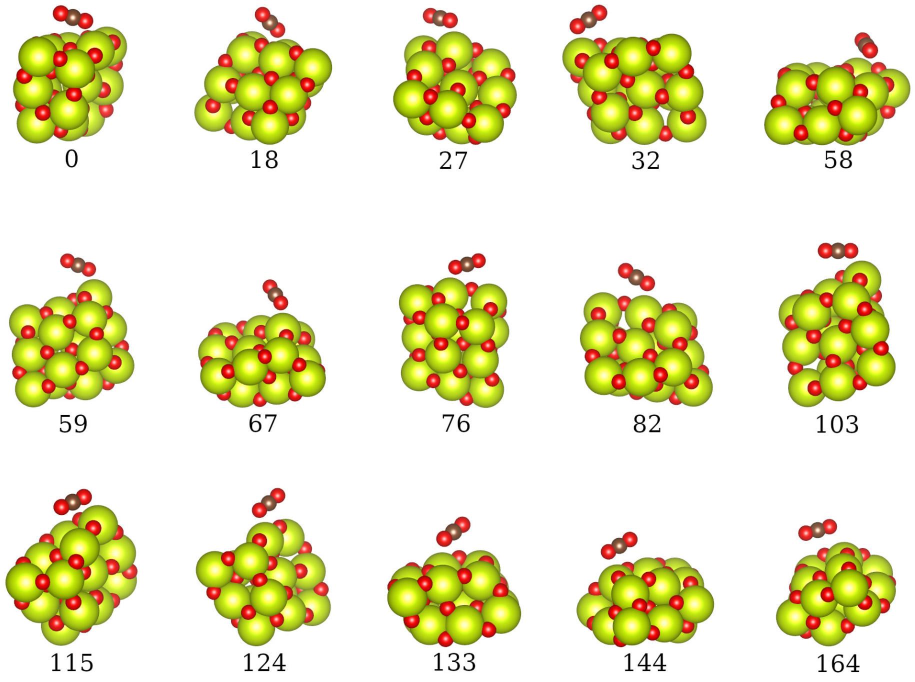

Figure S-7. Optimized $CO_2/Ce_{15}O_{30}$ structures. The relative total energy with respect to the lowest-energy configuration is displayed below each structure. All values are in meV.

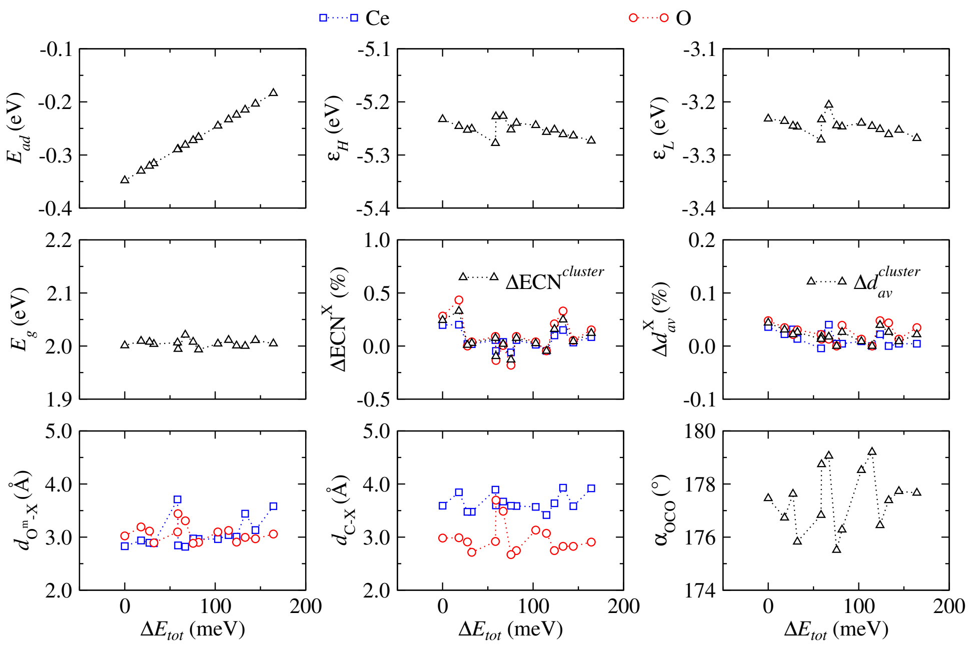

Figure S-8. Energetic, electronic, and structural properties for the $CO_2/Ce_{15}O_{30}$ systems. The description of each property is provided at the start of Section S-8.

S-8.5 CO_2 on $Ce_8Zr_7O_{30}$ Nanoclusters

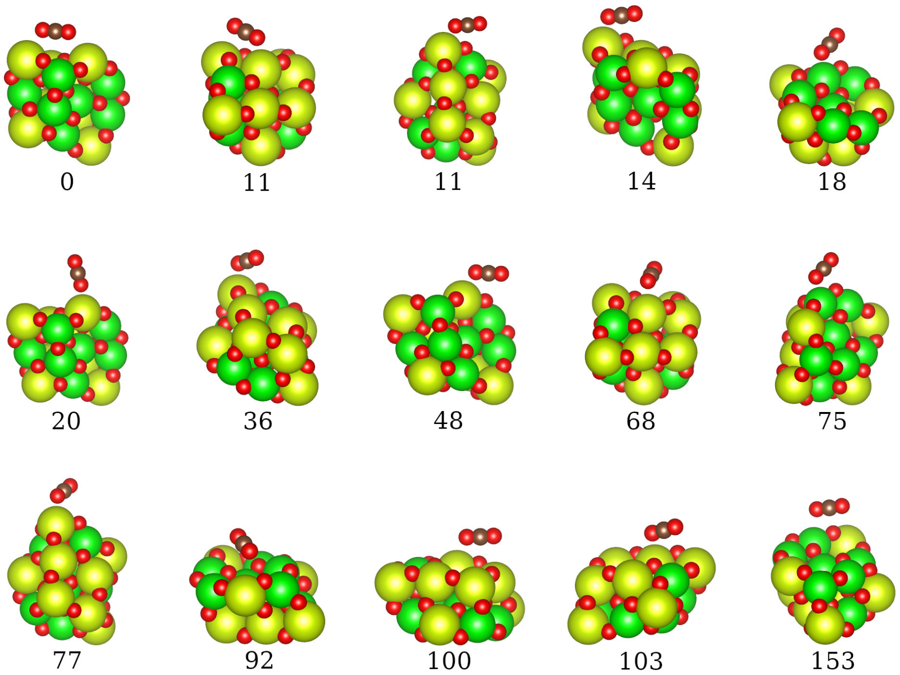

Figure S-9. Optimized $CO_2/Ce_8Zr_7O_{30}$ structures. The relative total energy with respect to the lowest-energy configuration is displayed below each structure. All values are in meV.

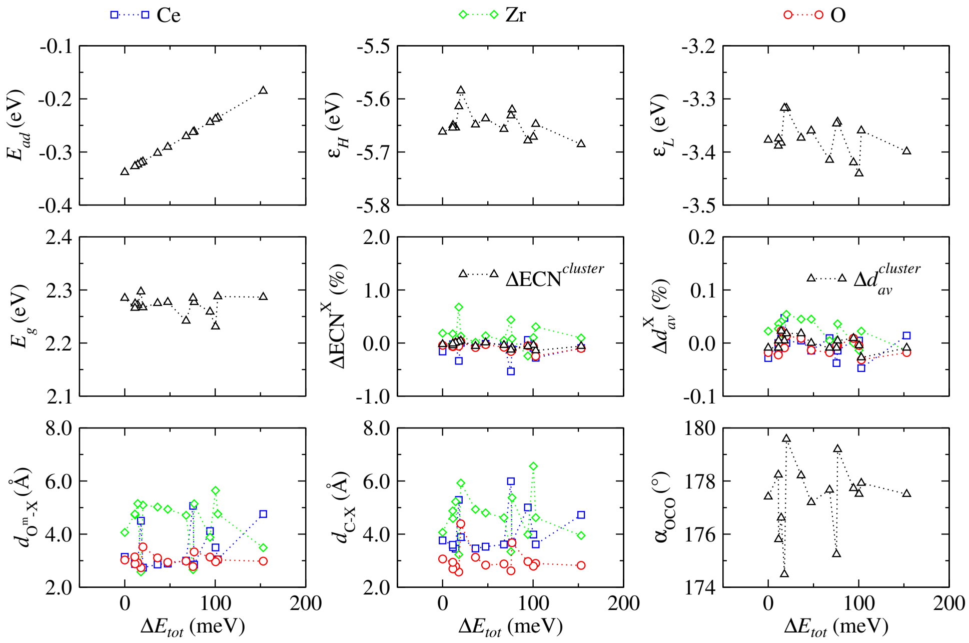

Figure S-10. Energetic, electronic, and structural properties for the $CO_2/Ce_8Zr_7O_{30}$ systems. The description of each property is provided at the start of Section S-8.

S-8.6 CO_2 on $Zr_{15}O_{30}$ Nanoclusters

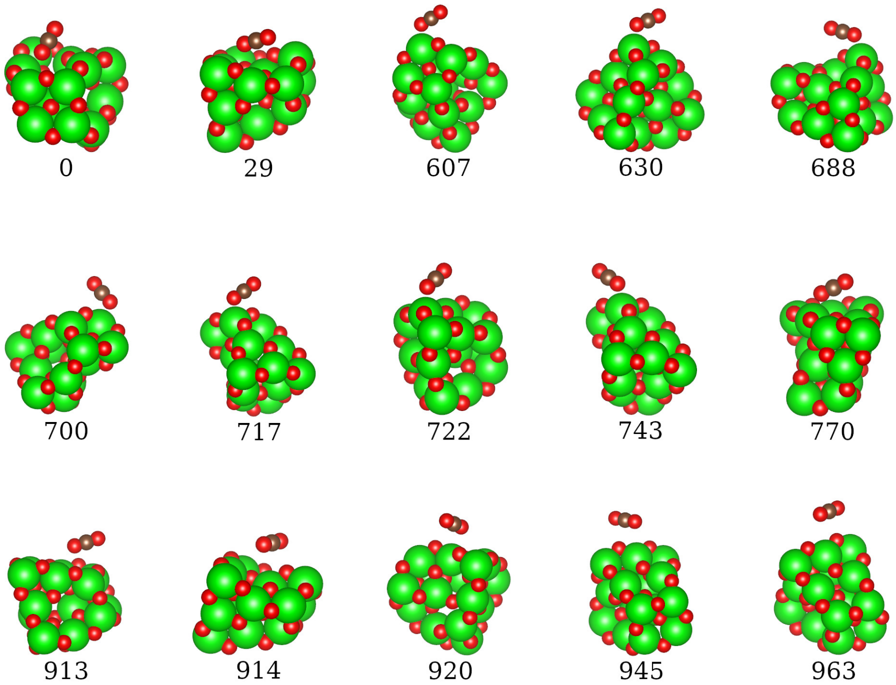

Figure S-11. Optimized $CO_2/Zr_{15}O_{30}$ structures. The relative total energy with respect to the lowest-energy configuration is displayed below each structure. All values are in meV.

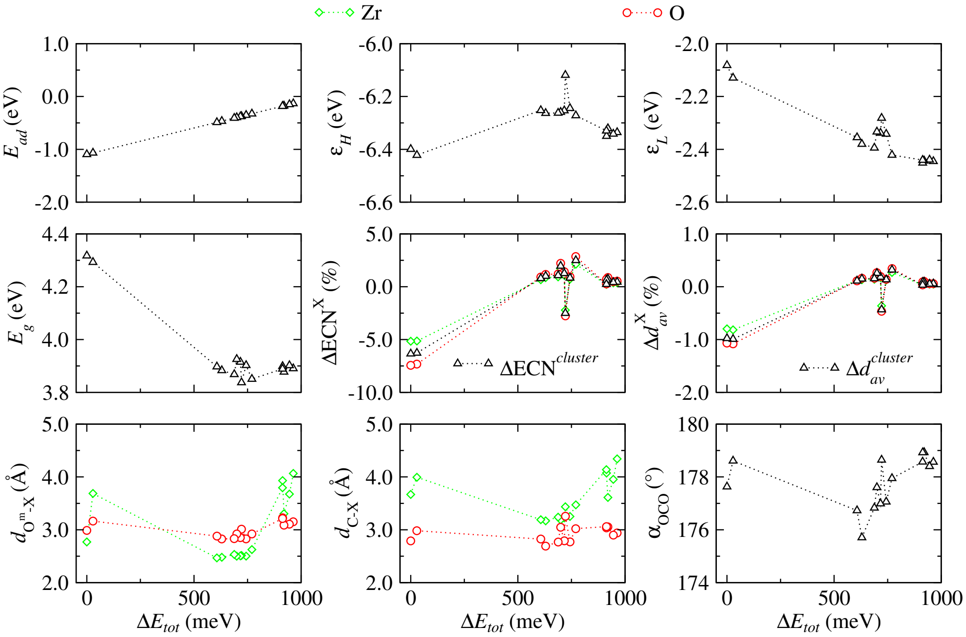

Figure S-12. Energetic, electronic, and structural properties for the $CO_2/Zr_{15}O_{30}$ systems. The description of each property is provided at the start of Section S-8.

S-8.7 CH_4 on $Ce_{15}O_{30}$ Nanoclusters

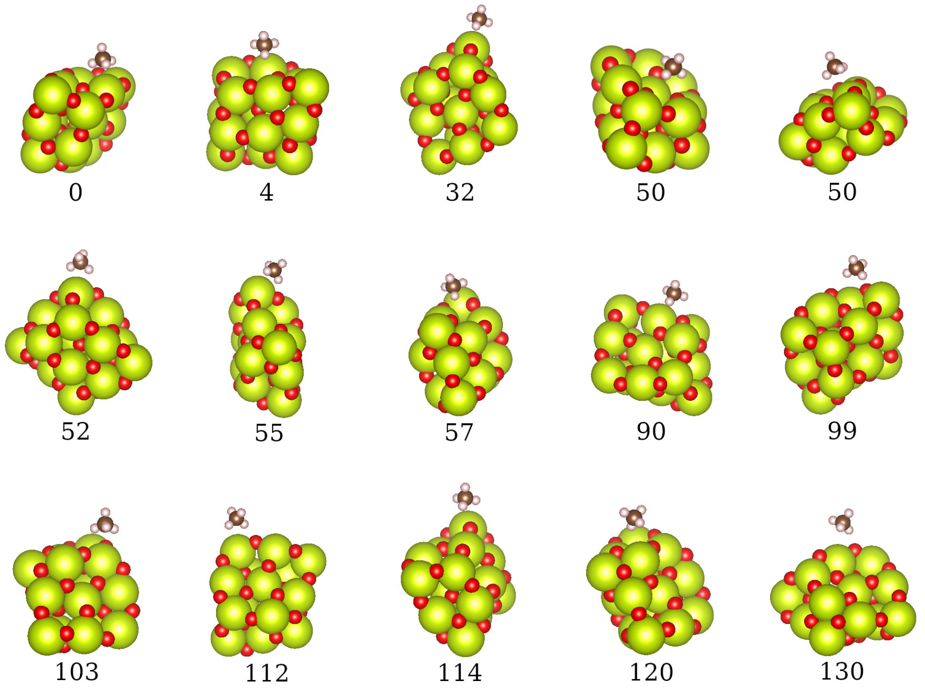

Figure S-13. Optimized $CH_4/Ce_{15}O_{30}$ structures. The relative total energy with respect to the lowest-energy configuration is displayed below each structure. All values are in meV.

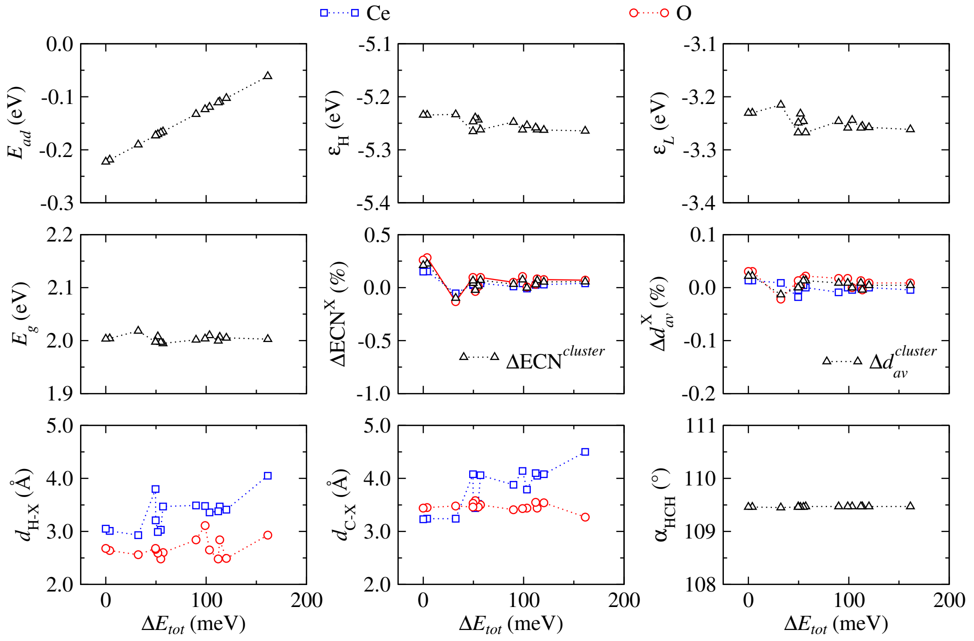

Figure S-14. Energetic, electronic, and structural properties for the $CH_4/Ce_{15}O_{30}$ systems. The description of each property is provided at the start of Section S-8.

S-8.8 CH_4 on $Ce_8Zr_7O_{30}$ Nanoclusters

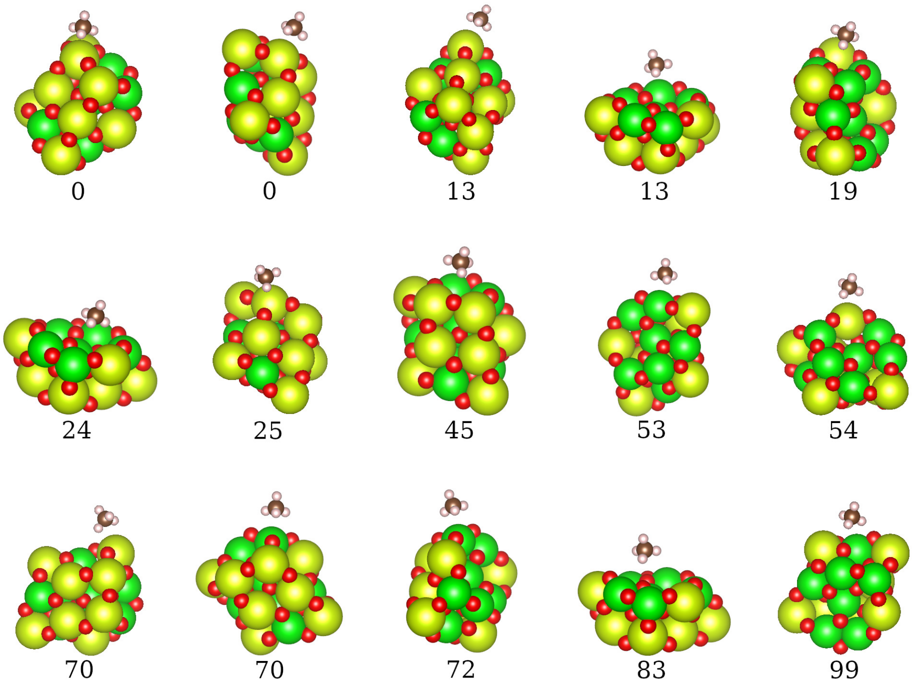

Figure S-15. Optimized $CH_4/Ce_8Zr_7O_{30}$ structures. The relative total energy with respect to the lowest-energy configuration is displayed below each structure. All values are in meV.

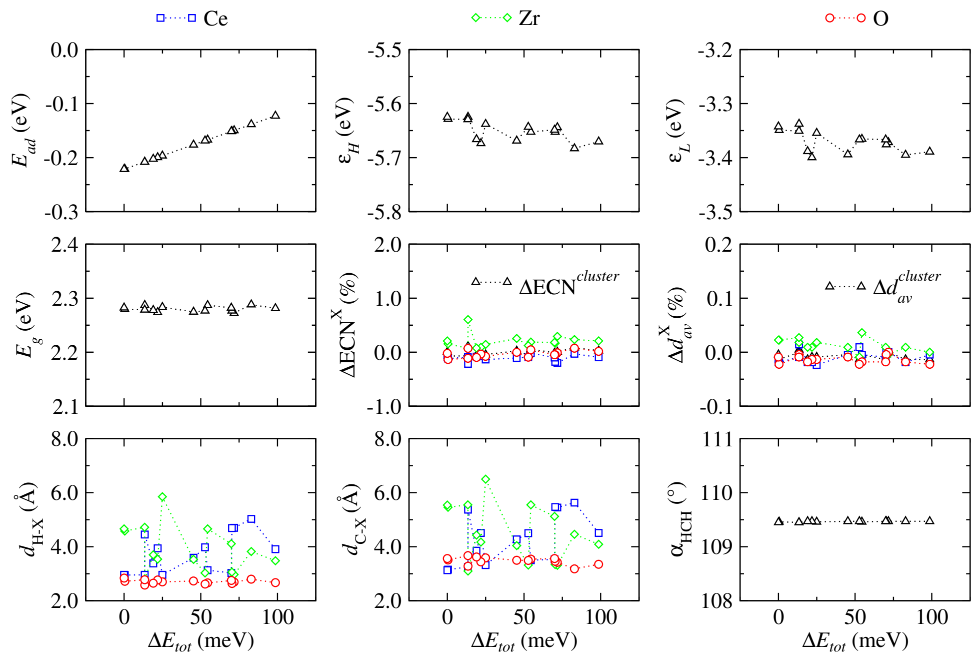

Figure S-16. Energetic, electronic, and structural properties for the $CH_4/Ce_8Zr_7O_{30}$ systems. The description of each property is provided at the start of Section S-8.

S-8.9 CH_4 on $Zr_{15}O_{30}$ Nanoclusters

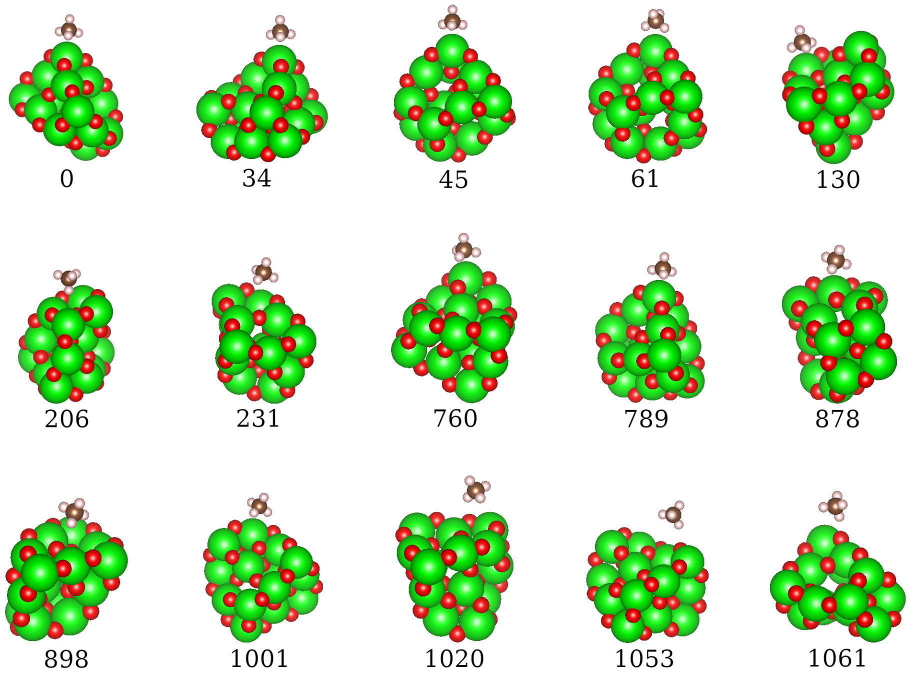

Figure S-17. Optimized $CH_4/Zr_{15}O_{30}$ structures. The relative total energy with respect to the lowest-energy configuration is displayed below each structure. All values are in meV.

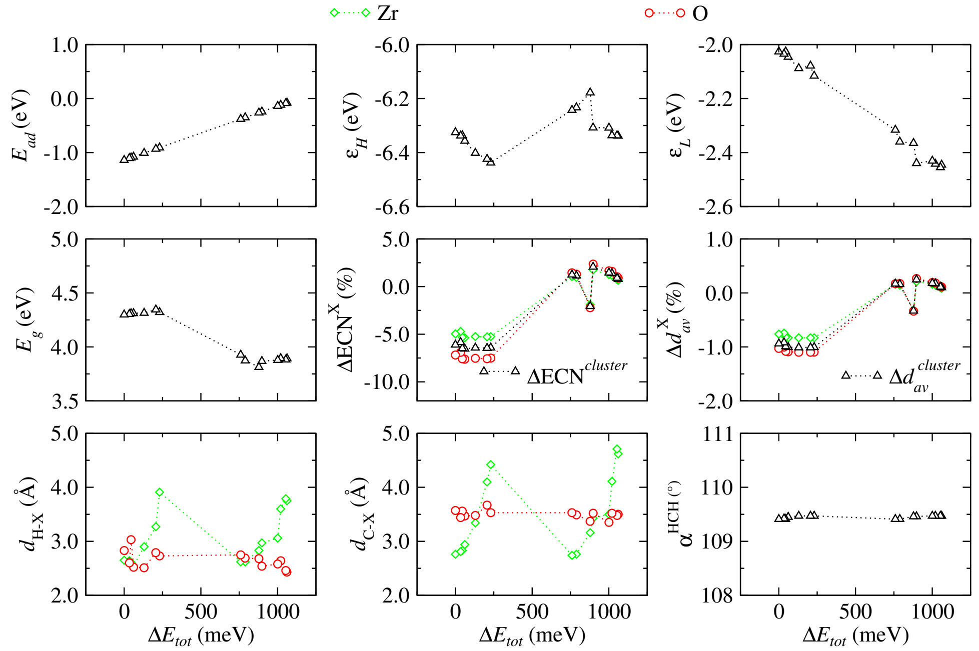

Figure S-18. Energetic, electronic, and structural properties for the $CH_4/Zr_{15}O_{30}$ systems. The description of each property is provided at the start of Section S-8.

S-8.10 NH_3 on $Ce_{15}O_{30}$ Nanoclusters

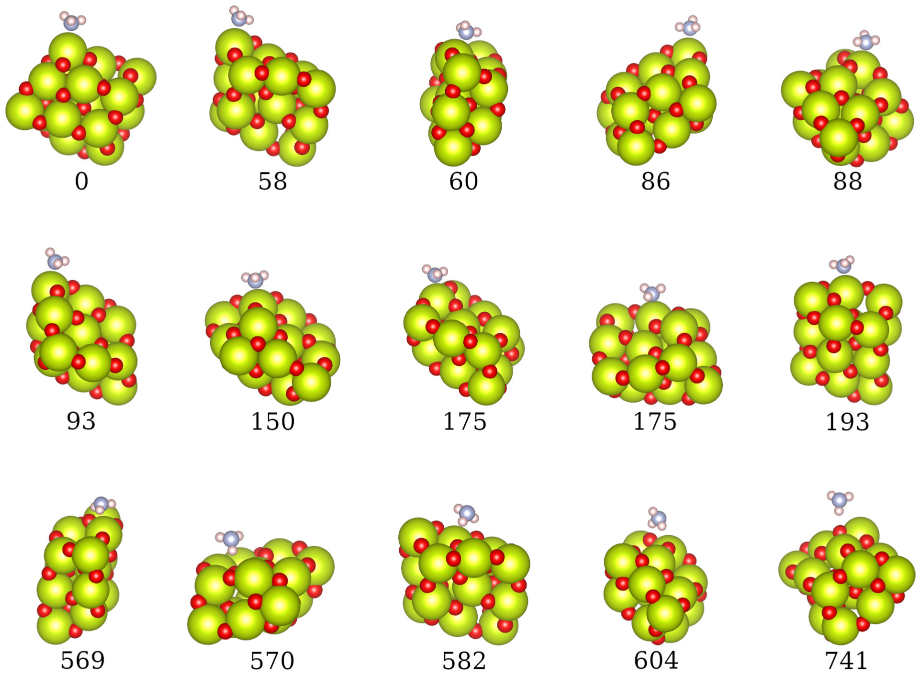

Figure S-19. Optimized $NH_3/Ce_{15}O_{30}$ structures. The relative total energy with respect to the lowest-energy configuration is displayed below each structure. All values are in meV.

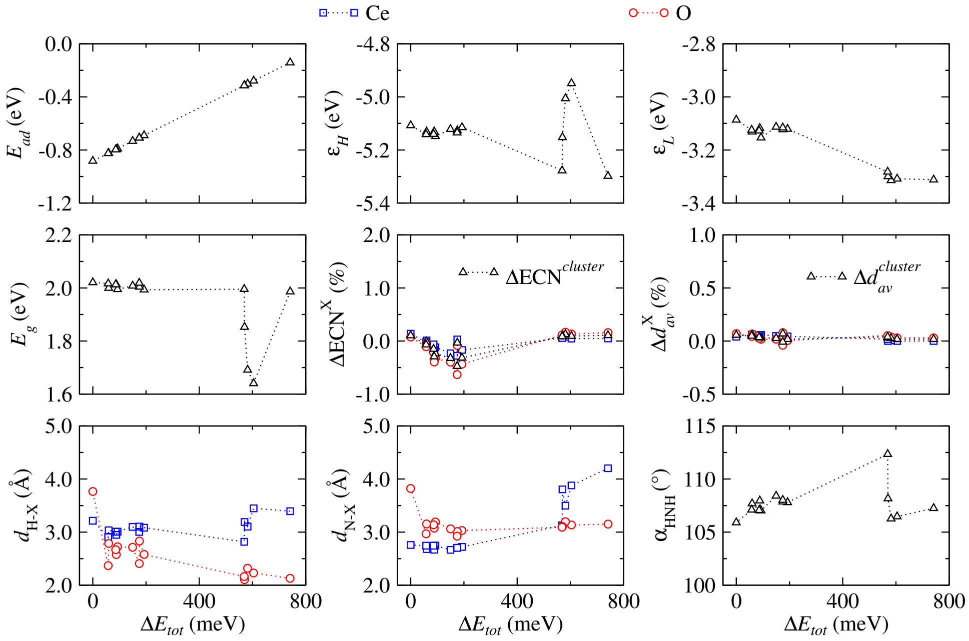

Figure S-20. Energetic, electronic, and structural properties for the $NH_3/Ce_{15}O_{30}$ systems. The description of each property is provided at the start of Section S-8.

S-8.11 NH_3 on $Ce_8Zr_7O_{30}$ Nanoclusters

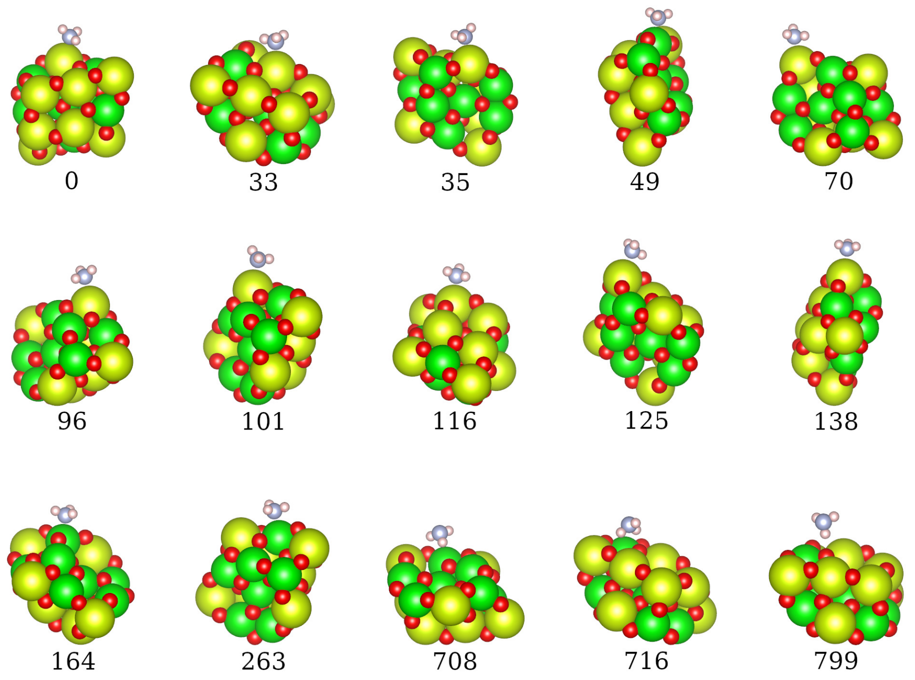

Figure S-21. Optimized $NH_3/Ce_8Zr_7O_{30}$ structures. The relative total energy with respect to the lowest-energy configuration is displayed below each structure. All values are in meV.

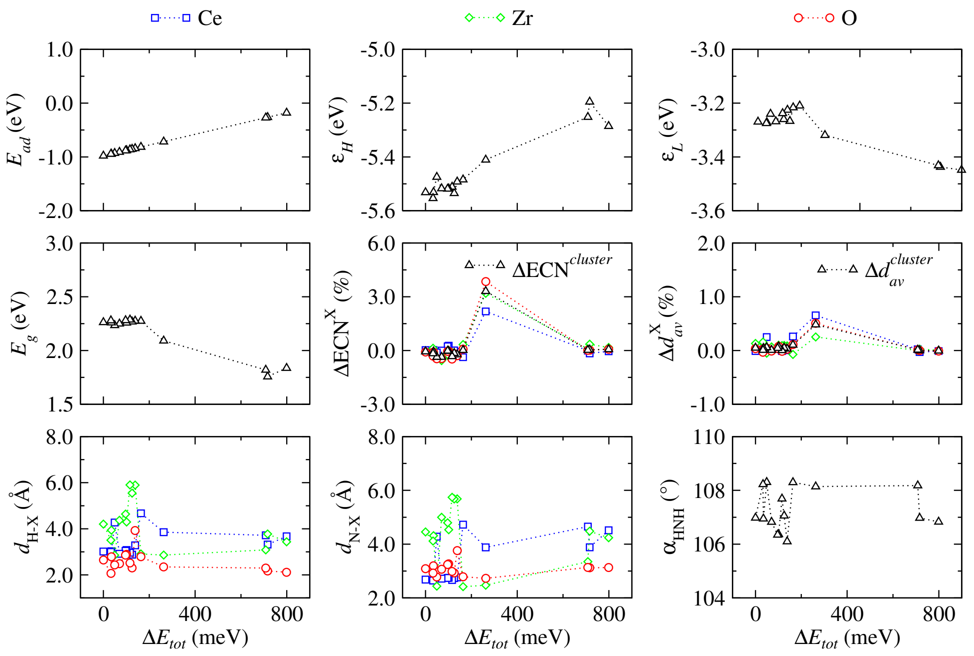

Figure S-22. Energetic, electronic, and structural properties for the $NH_3/Ce_8Zr_7O_{30}$ systems. The description of each property is provided at the start of Section S-8.

S-8.12 NH_3 on $Zr_{15}O_{30}$ Nanoclusters

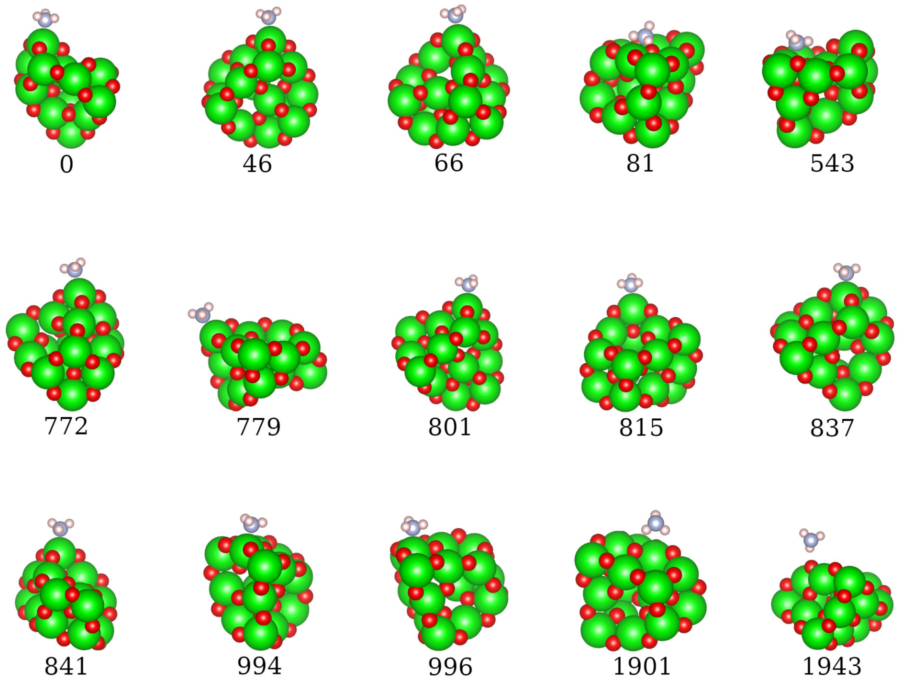

Figure S-23. Optimized $NH_3/Zr_{15}O_{30}$ structures. The relative total energy with respect to the lowest-energy configuration is displayed below each structure. All values are in meV.

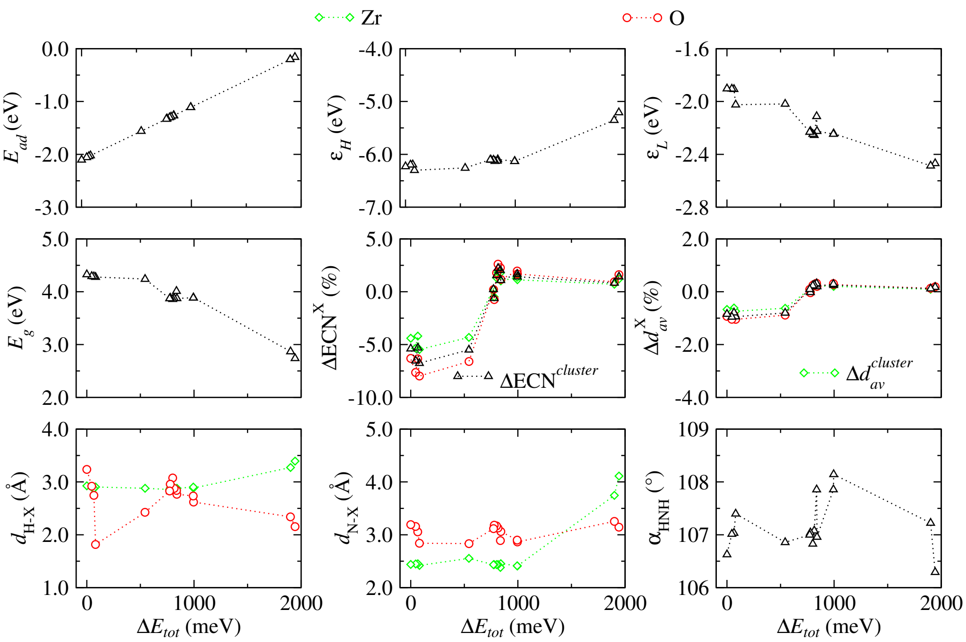

Figure S-24. Energetic, electronic, and structural properties for the $NH_3/Zr_{15}O_{30}$ systems. The description of each property is provided at the start of Section S-8.

S-8.13 H_2O on $Ce_{15}O_{30}$ Nanoclusters

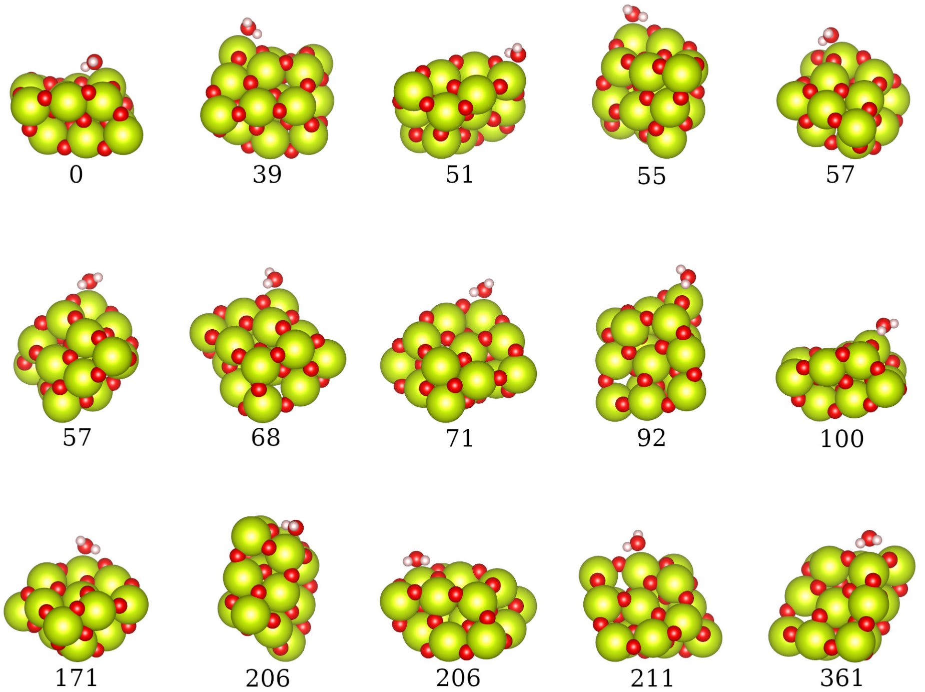

Figure S-25. Optimized $H_2O/Ce_{15}O_{30}$ structures. The relative total energy with respect to the lowest-energy configuration is displayed below each structure. All values are in meV.

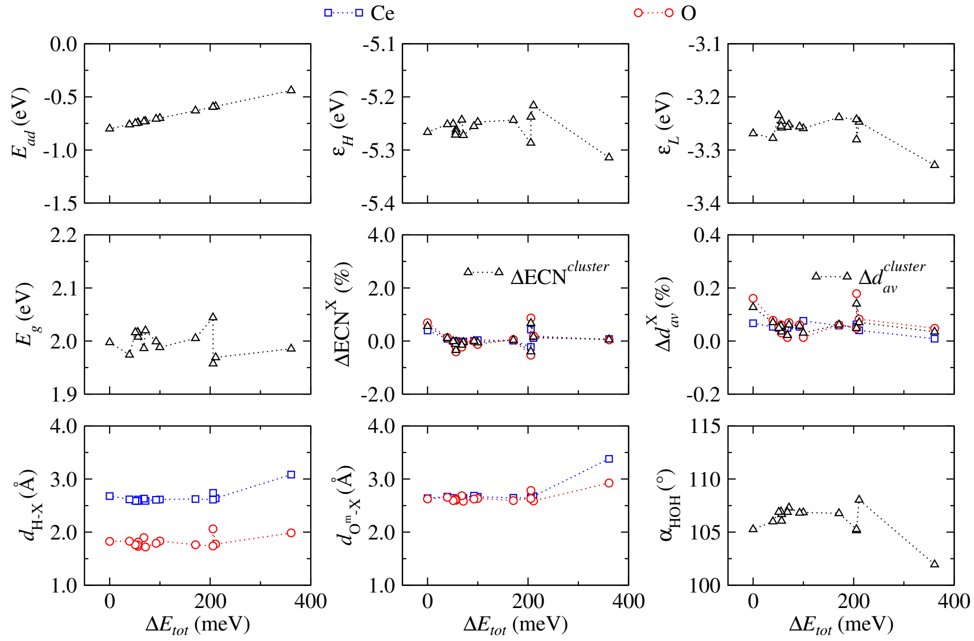

Figure S-26. Energetic, electronic, and structural properties for the $H_2O/Ce_{15}O_{30}$ systems. The description of each property is provided at the start of Section S-8.

S-8.14 H_2O on $Ce_8Zr_7O_{30}$ Nanoclusters

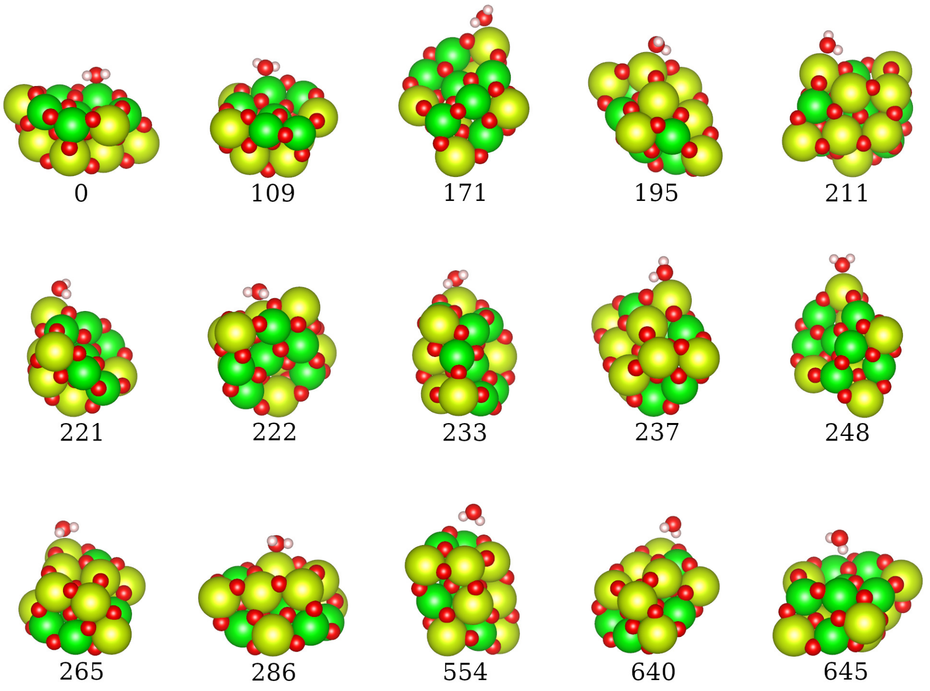

Figure S-27. Optimized $H_2O/Ce_8Zr_7O_{30}$ structures. The relative total energy with respect to the lowest-energy configuration is displayed below each structure. All values are in meV.

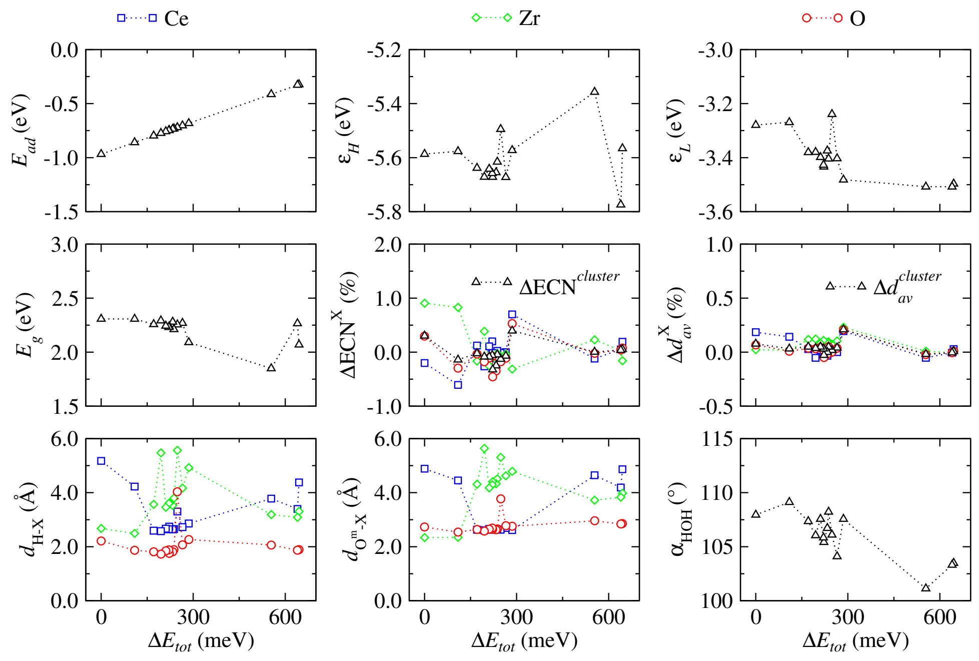

Figure S-28. Energetic, electronic, and structural properties for the $H_2O/Ce_8Zr_7O_{30}$ systems. The description of each property is provided at the start of Section S-8.

S-8.15 H_2O on $Zr_{15}O_{30}$ Nanoclusters

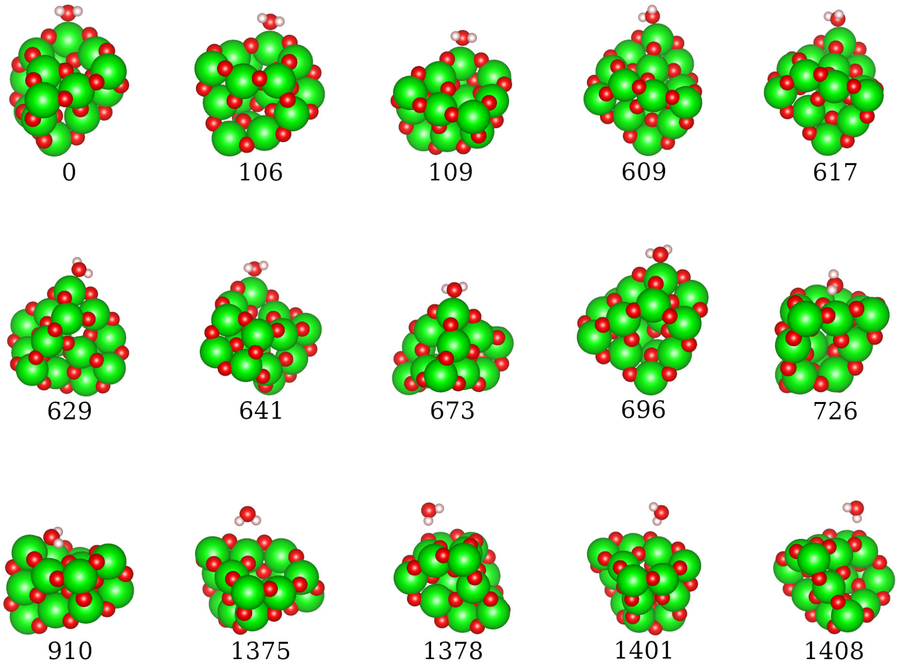

Figure S-29. Optimized $H_2O/Zr_{15}O_{30}$ structures. The relative total energy with respect to the lowest-energy configuration is displayed below each structure. All values are in meV.

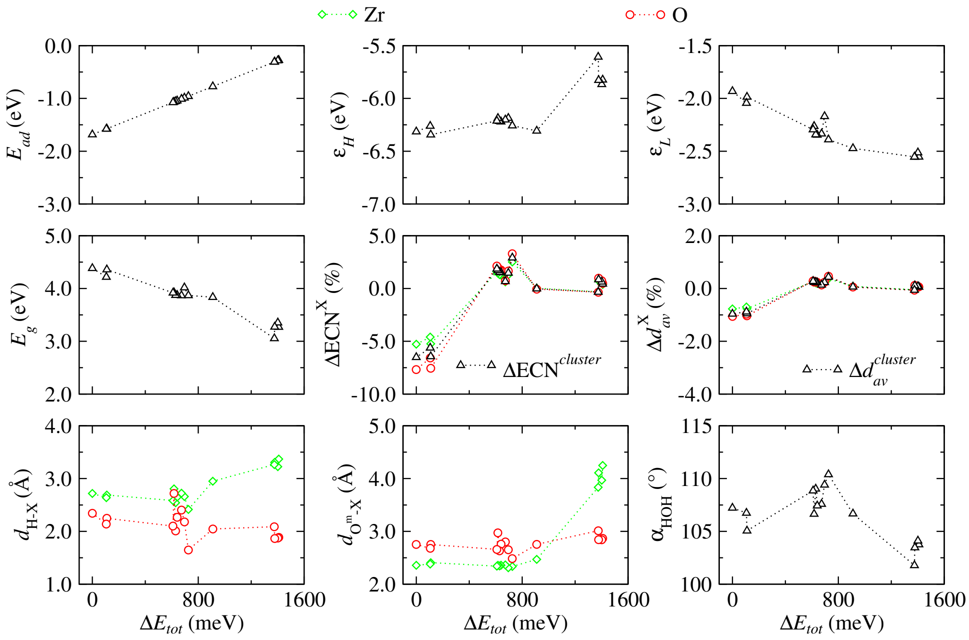

Figure S-30. Energetic, electronic, and structural properties for the $H_2O/Zr_{15}O_{30}$ systems. The description of each property is provided at the start of Section S-8.

S-8.16 SO_2 on $Ce_{15}O_{30}$ Nanoclusters

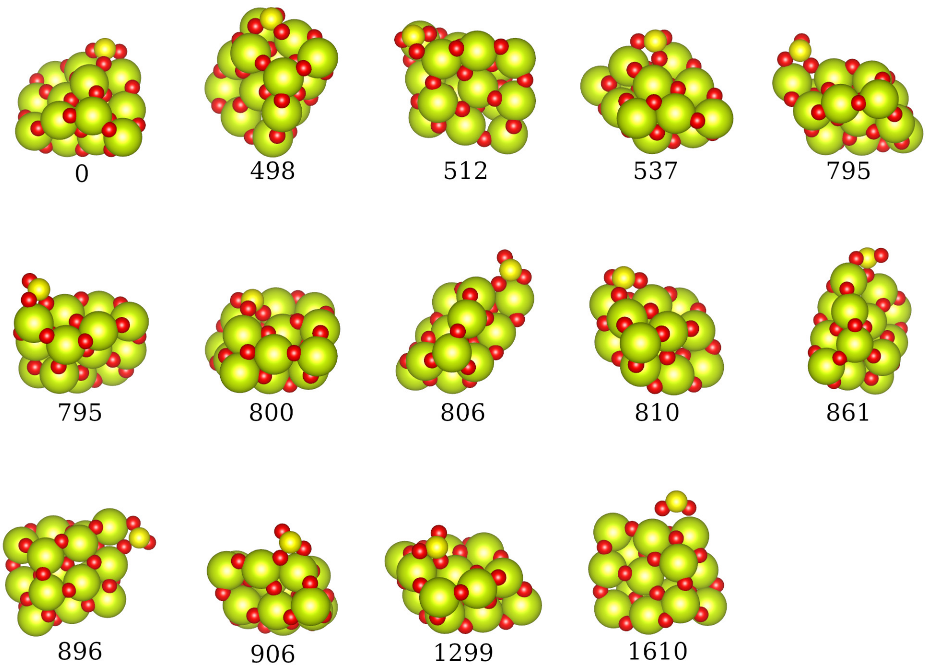

Figure S-31. Optimized $SO_2/Ce_{15}O_{30}$ structures. The relative total energy with respect to the lowest-energy configuration is displayed below each structure. All values are in meV.

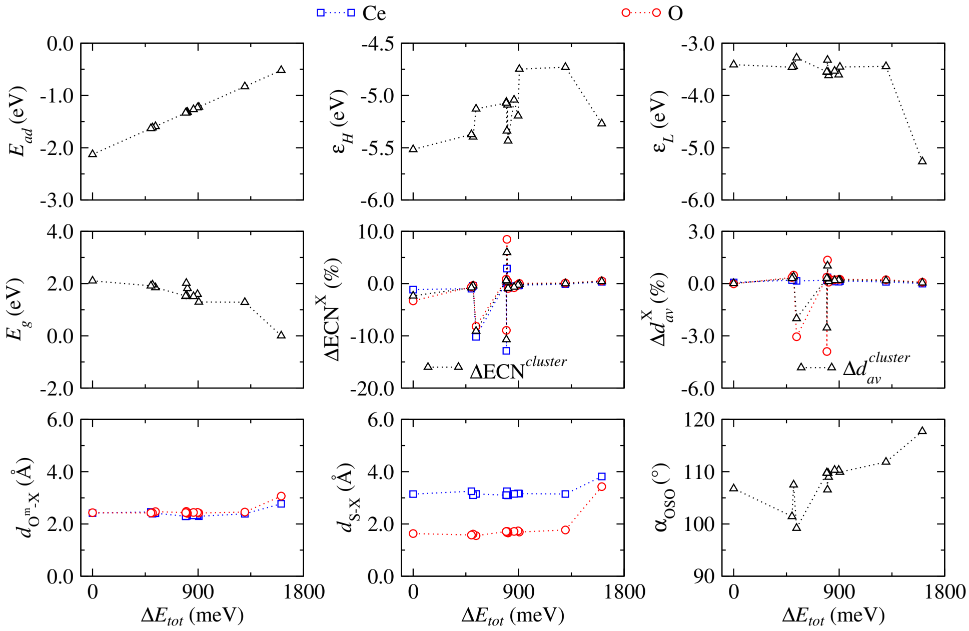

Figure S-32. Energetic, electronic, and structural properties for the $SO_2/Ce_{15}O_{30}$ systems. The description of each property is provided at the start of Section S-8.

S-8.17 SO_2 on $Ce_8Zr_7O_{30}$ Nanoclusters

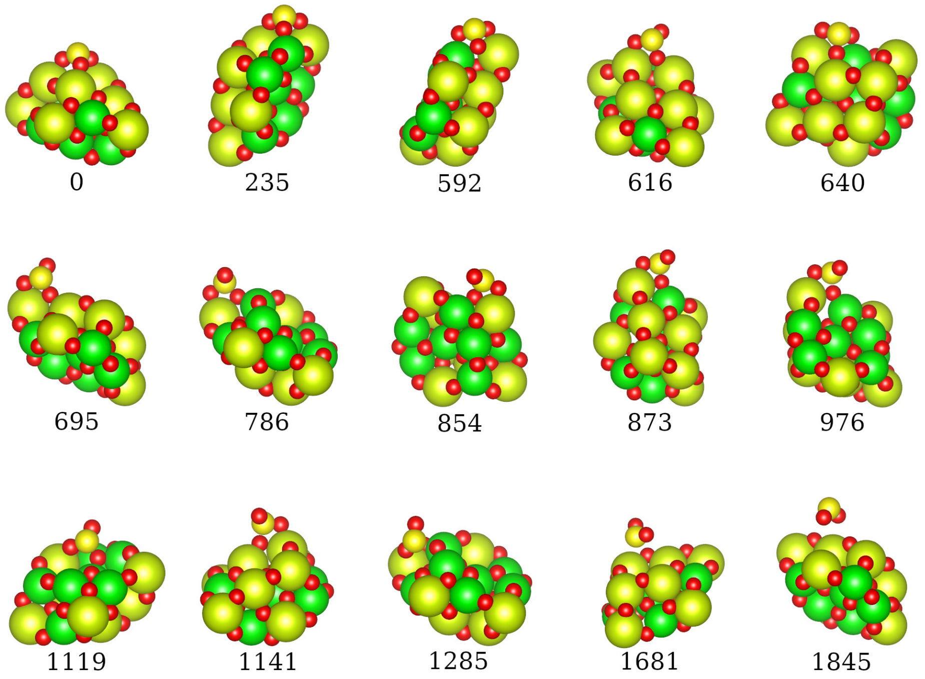

Figure S-33. Optimized $SO_2/Ce_8Zr_7O_{30}$ structures. The relative total energy with respect to the lowest-energy configuration is displayed below each structure. All values are in meV.

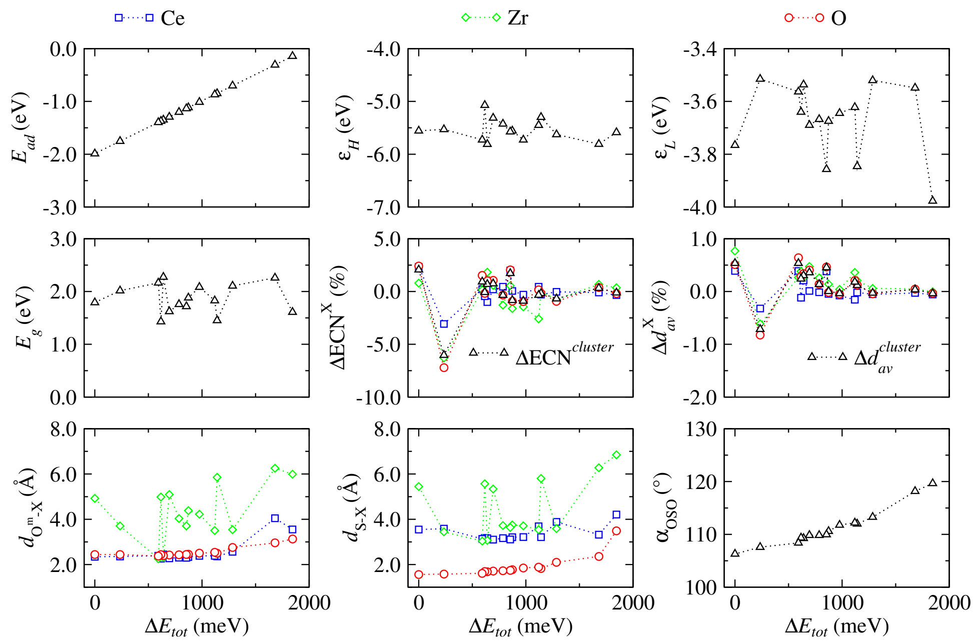

Figure S-34. Energetic, electronic, and structural properties for the $SO_2/Ce_8Zr_7O_{30}$ systems. The description of each property is provided at the start of Section S-8.

S-8.18 SO_2 on $Zr_{15}O_{30}$ Nanoclusters

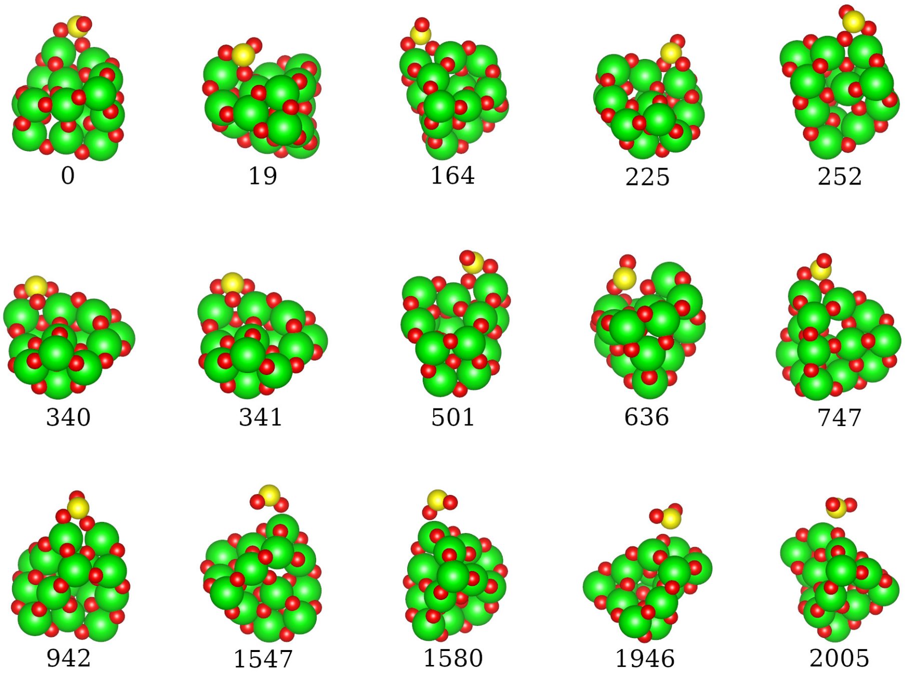

Figure S-35. Optimized $SO_2/Zr_{15}O_{30}$ structures. The relative total energy with respect to the lowest-energy configuration is displayed below each structure. All values are in meV.

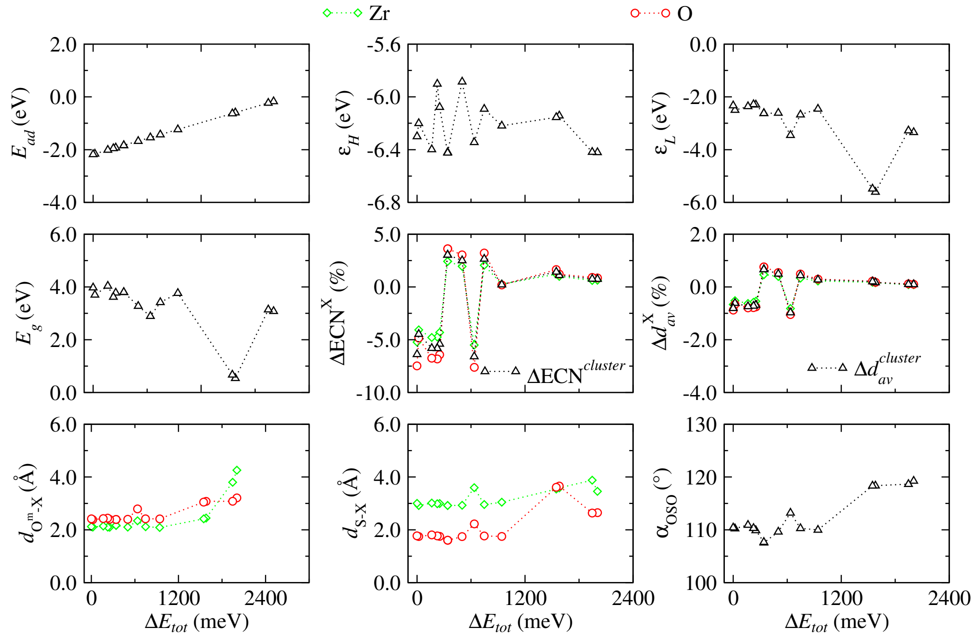

Figure S-36. Energetic, electronic, and structural properties for the $SO_2/Zr_{15}O_{30}$ systems. The description of each property is provided at the start of Section S-8.

Table S-8. Energetic data utilized to calculate adsorption and interaction energies. Total energy of adsorbed systems (E_{tot}), adsorption energy (E_{ad}), energy of molecules and substrates isolated in their frozen adsorbed geometries (E^{mol}_{frozen} and E^{sub}_{frozen}), and interaction energy (E_{in}). The E_{ad} was determined using the following energy values for the optimized gas-phase systems: −14.910 036 80 eV (CO), −23.174 530 63 eV (CO_2), −24.033 715 05 eV (CH_4), −19.549 295 58 eV (NH_3), −14.265 613 16 eV (H_2O), −17.011 432 26 eV (SO_2), −346.989 069 66 eV ($Ce_{15}O_{30}$), −390.673 372 69 eV ($Ce_8Zr_7O_{30}$) and −438.034 806 30 eV ($Zr_{15}O_{30}$).

Adsorption system	E_{tot} (eV)	E_{ad} (eV)	E^{mol}_{frozen} (eV)	E^{sub}_{frozen} (eV)	E_{in} (eV)
CO /$Ce_{15}O_{30}$	−362.240 371 03	−0.34	−14.909 681 09	−346.972 808 65	−0.36
CO /$Ce_8Zr_7O_{30}$	−406.054 679 31	−0.47	−14.908 911 91	−390.635 011 09	−0.51
CO /$Zr_{15}O_{30}$	−454.213 726 55	−1.27	−14.906 896 44	−438.807 582 46	−0.50
CO_2/$Ce_{15}O_{30}$	−370.511 558 37	−0.35	−23.164 597 90	−346.964 864 69	−0.38
CO_2/$Ce_8Zr_7O_{30}$	−414.186 106 39	−0.34	−23.169 741 65	−390.663 261 40	−0.35
CO_2/$Zr_{15}O_{30}$	−462.304 889 56	−1.10	−23.167 975 38	−438.785 367 72	−0.35
CH_4/$Ce_{15}O_{30}$	−371.245 476 69	−0.22	−24.028 920 77	−346.980 378 00	−0.24
CH_4/$Ce_8Zr_7O_{30}$	−414.928 461 54	−0.22	−24.027 229 59	−390.671 836 96	−0.23
CH_4/$Zr_{15}O_{30}$	−463.208 507 31	−1.14	−24.014 037 06	−438.813 216 53	−0.38
NH_3/$Ce_{15}O_{30}$	−367.421 436 52	−0.88	−19.548 906 99	−346.973 012 98	−0.90
NH_3/$Ce_8Zr_7O_{30}$	−411.201 335 57	−0.98	−19.546 929 06	−390.644 020 79	−1.01
NH_3/$Zr_{15}O_{30}$	−459.687 967 65	−2.10	−19.548 356 92	−438.760 902 69	−1.38
H_2O/$Ce_{15}O_{30}$	−362.054 788 42	−0.80	−14.247 346 13	−346.927 736 22	−0.88
H_2O/$Ce_8Zr_7O_{30}$	−405.906 860 22	−0.97	−14.251 937 37	−390.585 275 63	−1.07
H_2O/$Zr_{15}O_{30}$	−453.984 626 64	−1.68	−14.256 068 60	−438.743 203 37	−0.99
SO_2/$Ce_{15}O_{30}$	−366.128 235 75	−2.13	−16.472 295 28	−345.715 293 71	−3.94
SO_2/$Ce_8Zr_7O_{30}$	−409.673 303 67	−1.99	−16.303 521 43	−387.967 622 80	−5.40
SO_2/$Zr_{15}O_{30}$	−457.224 075 77	−2.18	−16.550 726 44	−438.080 276 26	−2.59

S-9 ADSORPTION MODES AND SITES PREFERENCE

Table S-9. Adsorption configurations closer to the centroids for CO/nanoclusters set, considering the number of atoms in the nanocluster (N_c) = 4, 6, 8 and the separation in k groups (g1 to gk) for clustering analysis.

N_c	4			6			8		
k	3	6	8	6	8	10	6	8	10
g1									
g2									
g3									
g4									
g5									
g6									
g7									
g8									
g9									
g10									

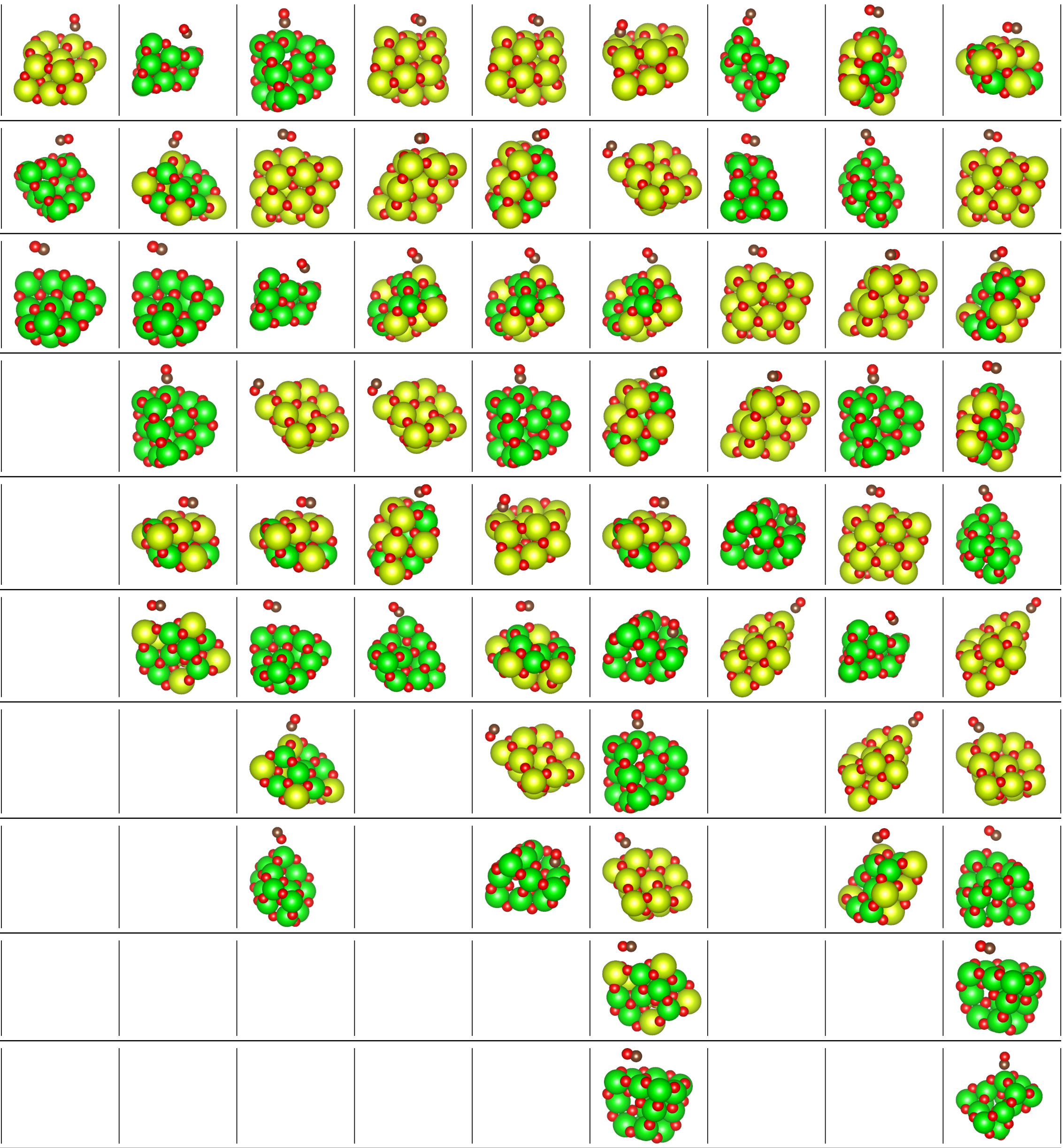

Table S-10. Adsorption configurations closer to the centroids for CO_2/nanoclusters set, considering the number of atoms in the nanocluster (N_c) = 4, 6, 8 and the separation in k groups (g1 to gk) for clustering analysis.

N_c	4			6			8		
k	5	8	11	4	9	14	7	9	12
g1	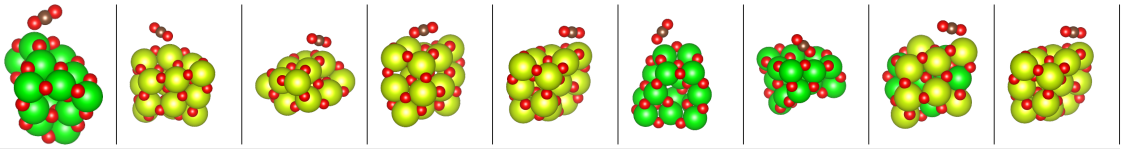								
g2	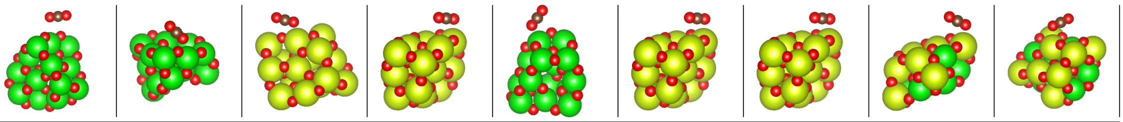								
g3	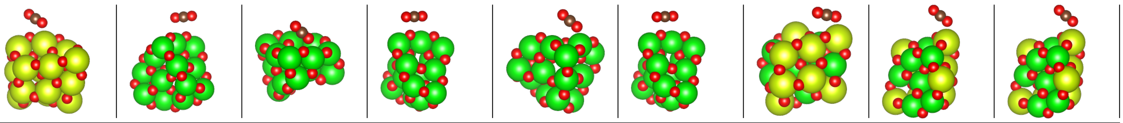								
g4	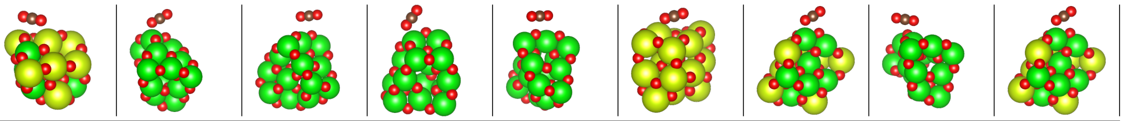								
g5	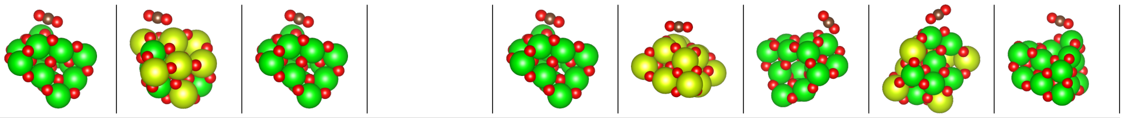								
g6		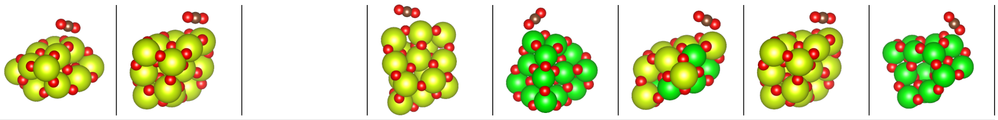							
g7		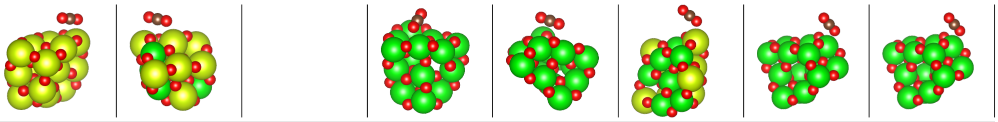							
g8		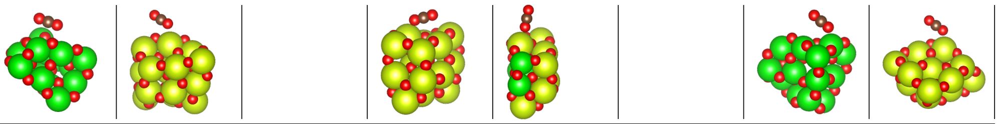							
g9			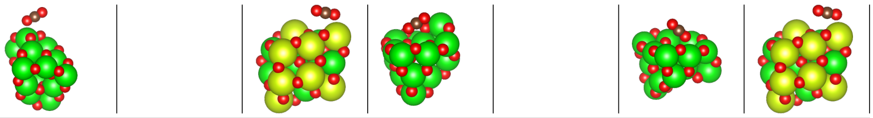						
g10			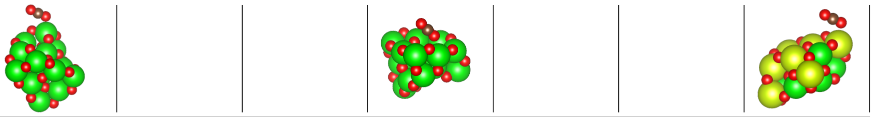						
g11			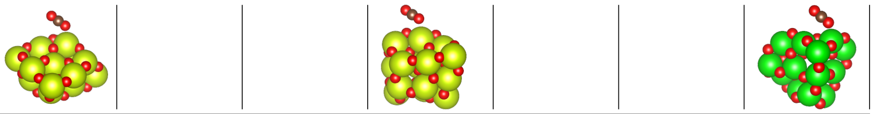						
g12						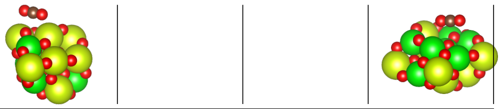			
g13						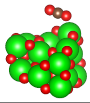			
g14						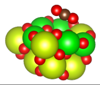			

Table S-11. Adsorption configurations closer to the centroids for CH_4/nanoclusters set, considering the number of atoms in the nanocluster (N_c) = 4, 6, 8 and the separation in *k* groups (g1 to g*k*) for clustering analysis.

N_c	4			6			8		
k	3	8	12	3	8	11	6	10	13
g1	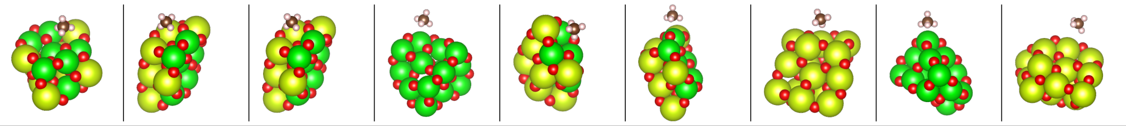								
g2	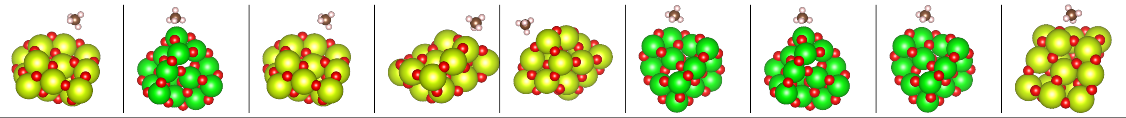								
g3	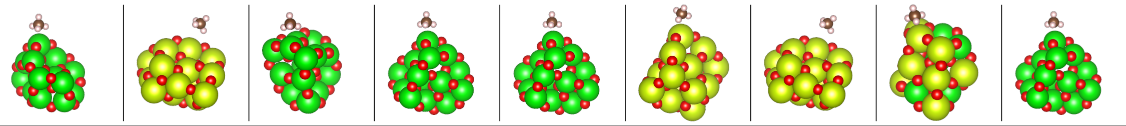								
g4	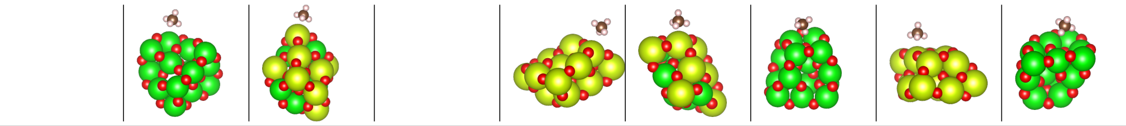								
g5	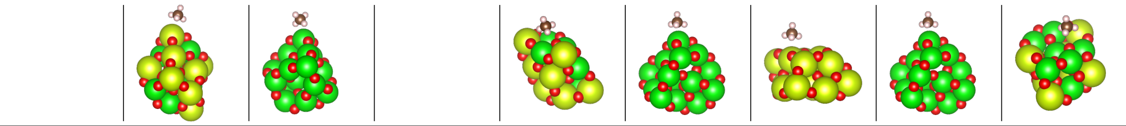								
g6	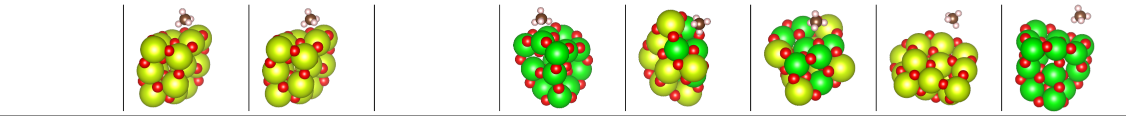								
g7	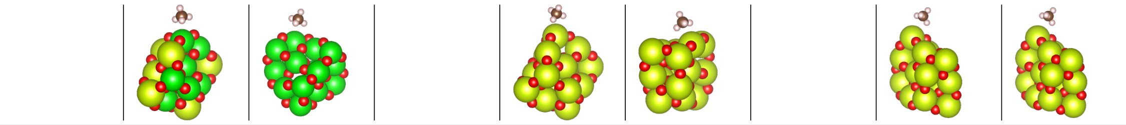								
g8	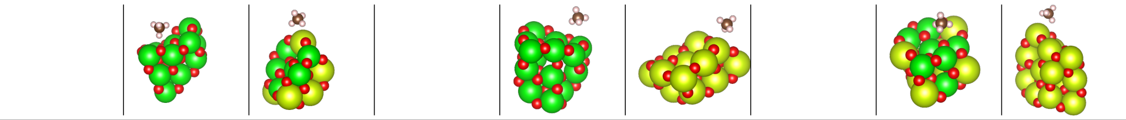								
g9	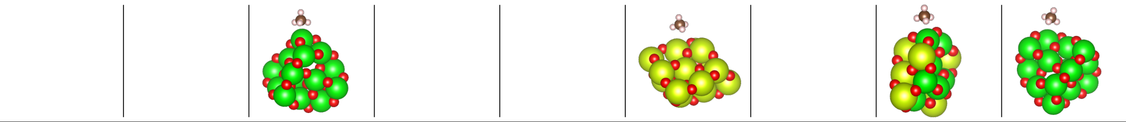								
g10	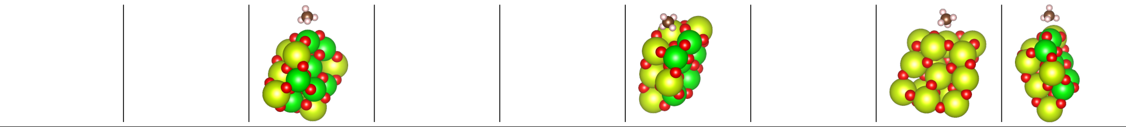								
g11	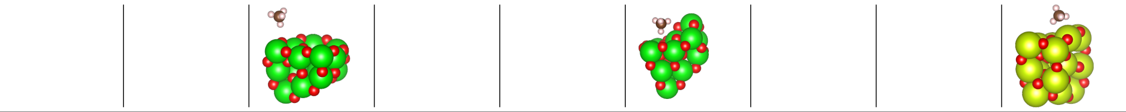								
g12	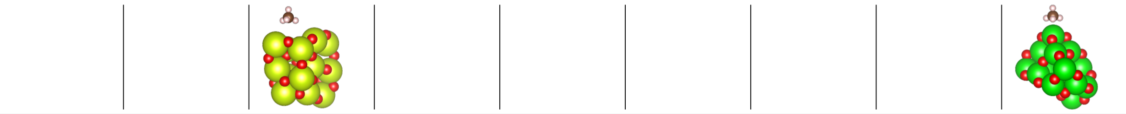								
g13	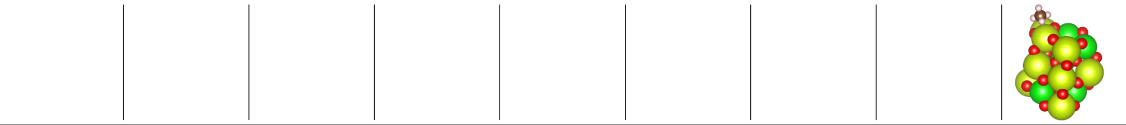								

Table S-12. Adsorption configurations closer to the centroids for NH_3/nanoclusters set, considering the number of atoms in the nanocluster (N_c) = 4, 6, 8 and the separation in k groups (g1 to gk) for clustering analysis.

N_c	4			6			8		
k	3	8	11	3	10	12	3	6	10
g1									
g2									
g3									
g4									
g5									
g6									
g7									
g8									
g9									
g10									
g11									
g12									

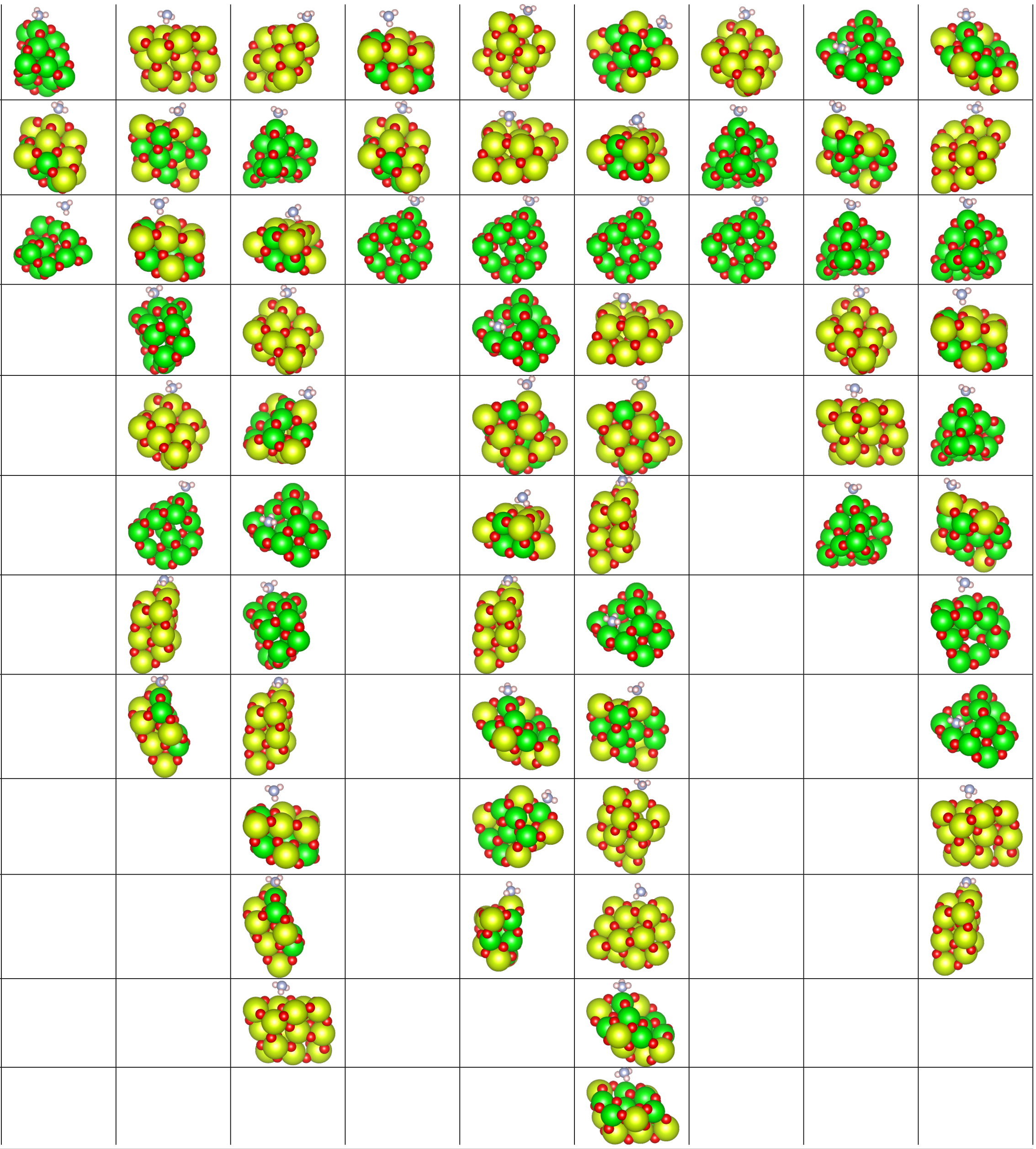

Table S-13. Adsorption configurations closer to the centroids for H_2O/nanoclusters set, considering the number of atoms in the nanocluster (N_c) = 4, 6, 8 and the separation in *k* groups (g1 to g*k*) for clustering analysis.

N_c	4			6			8		
k	3	6	9	6	9	14	5	9	14
g1									
g2									
g3									
g4									
g5									
g6									
g7									
g8									
g9									
g10									
g11									
g12									
g13									
g14									

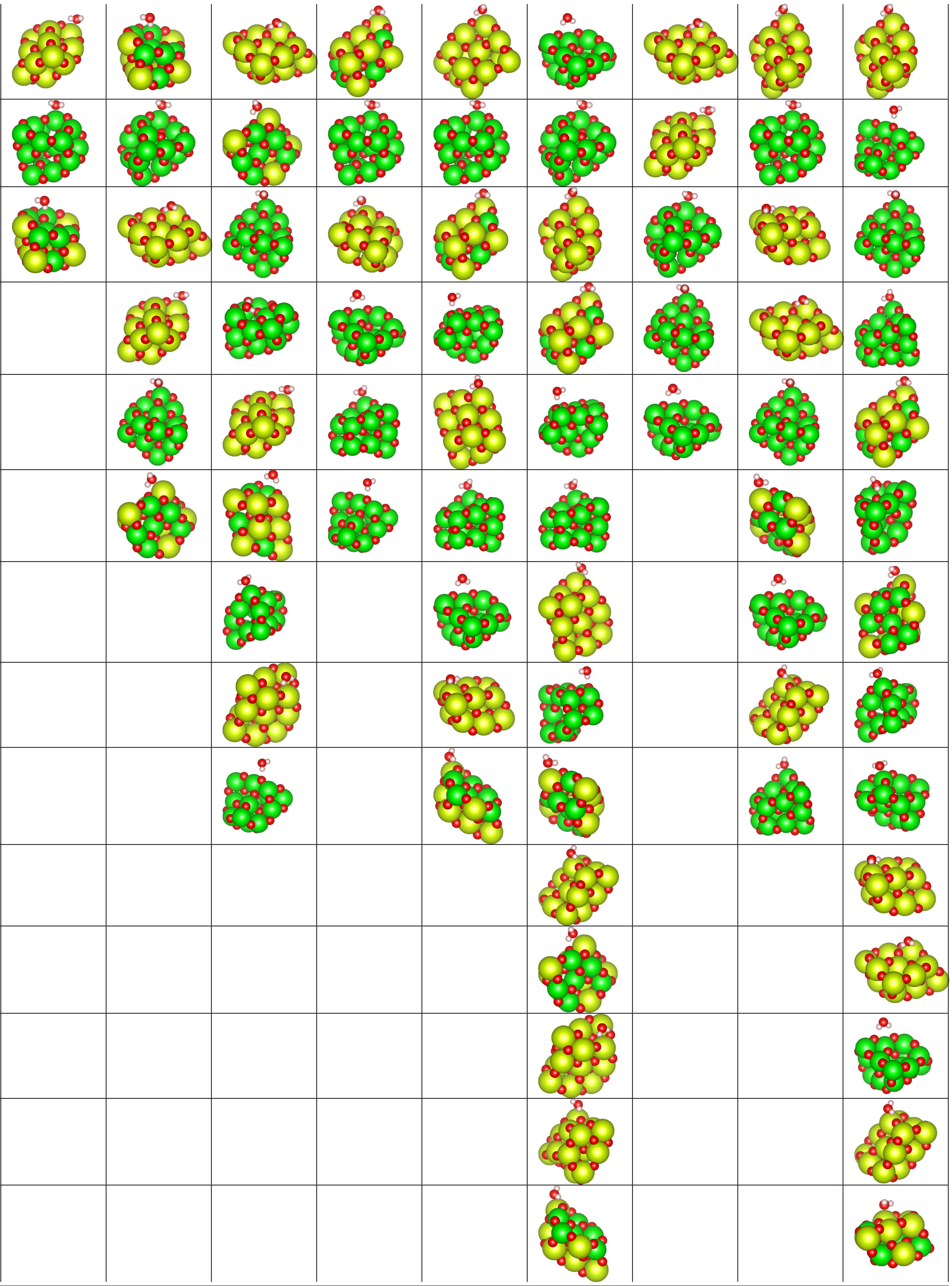

Table S-14. Adsorption configurations closer to the centroids for SO_2/nanoclusters set, considering the number of atoms in the nanocluster (N_c) = 4, 6, 8 and the separation in k groups (g1 to gk) for clustering analysis.

N_c	4			6			8		
k	3	7	9	5	7	9	3	6	10
g1	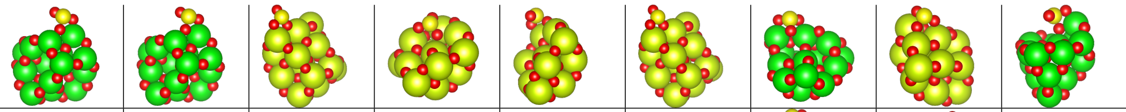								
g2	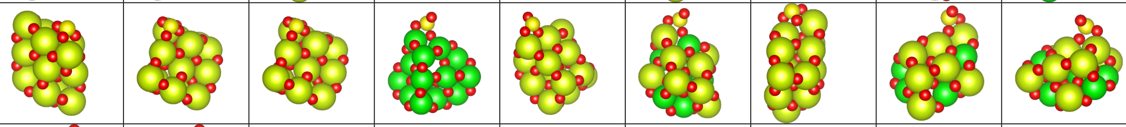								
g3	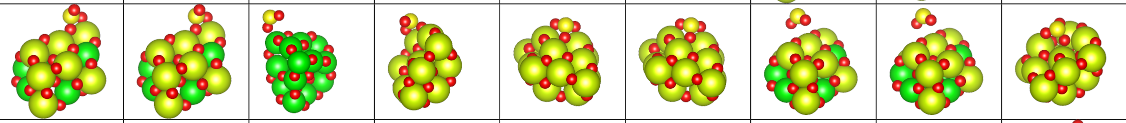								
g4	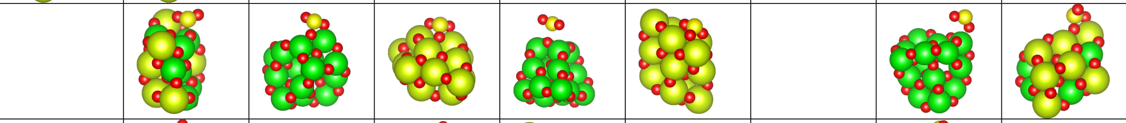								
g5	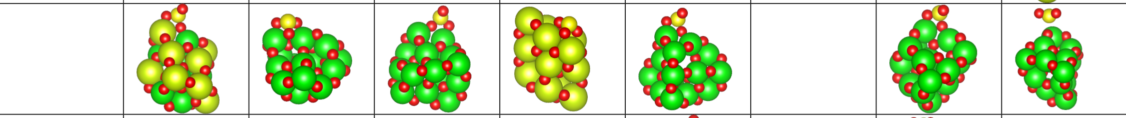								
g6	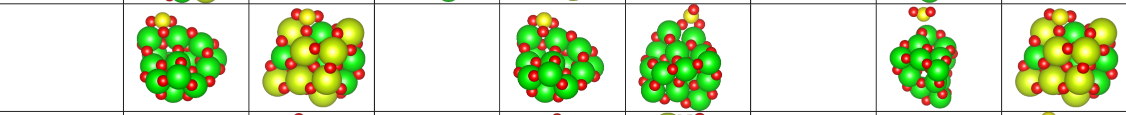								
g7	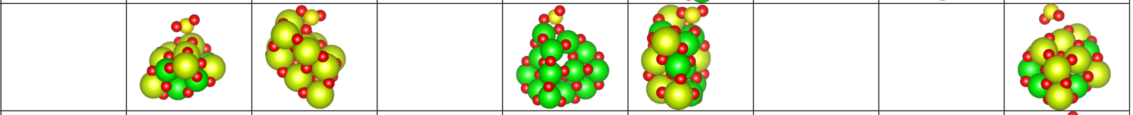								
g8	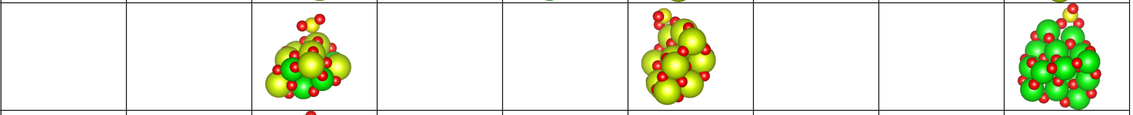								
g9	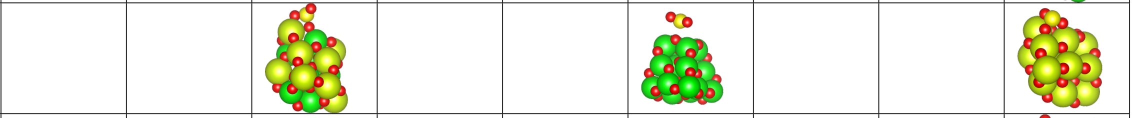								
g10	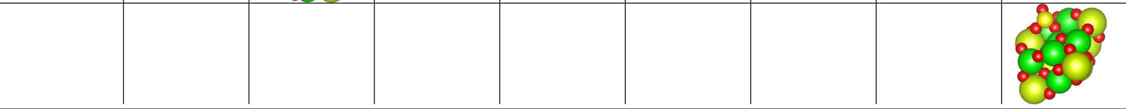								

References

1 Kerker, G. P. Efficient Iteration Scheme for Self-consistent Pseudopotential Calculations. *Phys. Rev. B* **1981**, *23*, 3082–3084, DOI: 10.1103/PhysRevB.23.3082.

2 Cha, S.-H. Comprehensive Survey on Distance/Similarity Measures Between Probability Density Functions. *City* **2007**, *1*, 1.

3 Jain, A. K. Data Clustering: 50 years Beyond K-means. *Pattern Recognit. Lett.* **2010**, *31*, 651–666, DOI: 10.1016/j.patrec.2009.09.011.

4 Batista, K. E. A.; Soares, M. D.; Quiles, M. G.; Piotrowski, M. J.; Da Silva, J. L. F. Energy Decomposition to Access the Stability Changes Induced by CO Adsorption on Transition-Metal 13-Atom Clusters. *J. Chem. Inf. Model.* **2021**, *61*, 2294–2301, DOI: 10.1021/acs.jcim.1c00097, PMID: 33939914.

5 MacQueen, J. B. Some Methods for Classification and Analysis of MultiVariate Observations. Proc. of the fifth Berkeley Symposium on Mathematical Statistics and Probability. 1967; pp 281–297.

6 Rupp, M.; Tkatchenko, A.; Müller, K.-R.; von Lilienfeld, O. A. Fast and Accurate Modeling of Molecular Atomization Energies with Machine Learning. *Phys. Rev. Lett.* **2012**, *108*, 058301, DOI: 10.1103/PhysRevLett.108.058301.

7 Rousseeuw, P. J. Silhouettes: A Graphical Aid to the Interpretation and Validation of Cluster Analysis. *J. Comput. Appl. Math.* **1987**, *20*, 53–65, DOI: 10.1016/0377-0427(87)90125-7.

8 Abdi, H.; Williams, L. J. Principal Component Analysis. *WIREs Comput. Stat.* **2010**, *2*, 433–459, DOI: 10.1002/wics.101.

9 van der Maaten, L.; Hinton, G. Visualizing Data using t-SNE. *J. Mach. Learn. Res.* **2008**, *9*, 2579–2605.

10 Da Silva, J. L. F. Effective Coordination Concept Applied for Phase Change $(GeTe)_m(Sb_2Te_3)_n$ Compounds. *J. Appl. Phys.* **2011**, *109*, 023502, DOI: 10.1063/1.3533422.

11 Haynes, W. M. *CRC Handbook of Chemistry and Physics, 94th Edition*; Taylor & Francis Limited, 2013.

12 Hush, N. S.; Williams, M. L. Carbon Monoxide Bond Length, Force Constant and Infrared Intensity Variations in Strong Electric Fields: Valence-shell Calculations, With Applications to Properties of Adsorbed and Complexed CO. *J. Mol. Spectrosc.* **1974**, *50*, 349–368, DOI: 10.1016/0022-2852(74)90241-0.

13 Harmony, M. D.; Laurie, V. W.; Kuczkowski, R. L.; Schwendeman, R. H.; Ramsay, D. A.; Lovas, F. J.; Lafferty, W. J.; Maki, A. G. Molecular Structures of Gas-Phase Polyatomic Molecules Determined by Spectroscopic Methods. *J. Phys. Chem. Ref. Data* **1979**, *8*, 619–721, DOI: 10.1063/1.555605.

14 Chantry, P. J. Dissociative Attachment in Carbon Dioxide. *J. Chem. Phys.* **1972**, *57*, 3180–3186, DOI: 10.1063/1.1678736.
